# The Nodal Structure of π‑Orbitals Is Mapped in the Interaction Energy of π‑Stacked Acene Dimers

**DOI:** 10.1021/jacs.6c00921

**Published:** 2026-04-09

**Authors:** Michael Thelen, Johannes F. Henrichsmeyer, Andrea Buchwald, Reinhold F. Fink

**Affiliations:** Institute of Physical and Theoretical Chemistry, 9188 Universität Tübingen, Auf der Morgenstelle 18, Tübingen 72076, Germany

## Abstract

We show and explain that repulsive contributions to intermolecular interactions have a decisive influence on the energetic landscape of prototypical π-stacked systems. Remarkably, this results from the wave nature of the respective π-orbitals, which is reflected in the exchange repulsion energy, *E*_xr_. This is rationalized by partitioning *E*_xr_ into orbital–orbital contributions. The latter were recently proposed as the Molecular Orbital-Pair Contributions to the Exchange repulsion (MOPCE) approach, which allows to distinguish between σ–σ-, π–σ-, and π–π-contributions to *E*_xr_. For parallel displaced acene dimers with constant inter planar distance the π–π-contributions are shown to cause oscillations in the interaction energy as a function of monomer displacements. The MOPCE partitioning also allows to relate these features to the squared overlap of the π-orbitals, which can be reproduced using a simple particle-in-a-box model. Our approach rationalizes the interaction energies of benzene, naphthalene, anthracene, tetracene, and pentacene dimers obtained with Symmetry Adapted Perturbation Theory (SAPT). The presented results shed light on the nature of the exchange repulsion energy as a quantum mechanical property by showing that it can be represented in terms of orbital-pair contributions that are intuitively accessible by considering the nodal structure of orbitals.

## Graphical abstract

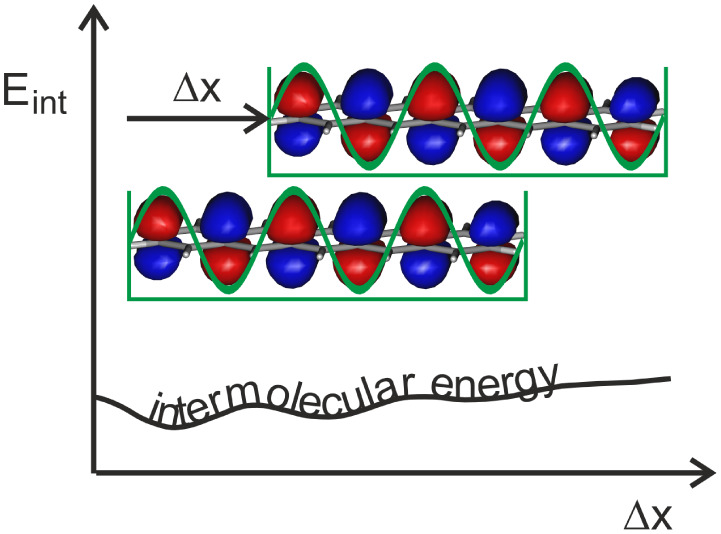

## Introduction

π-Interactions have received significant attention due to their widespread occurrence and importance in biology, chemistry, pharmacy and material science.^1–7^ Their influence on the structure of proteins is well established^1,8,9^ and it has been shown recently^10^ that stacked structures with parallel orientations of neighboring π-systems occur most frequently. Benzene and acene dimers show prototypical π-interactions of aromatic compounds and have been intensely studied for this reason but also as some of them are of interest as optoelectronic organic materials with high charge mobility.^11–22^ The particularly well studied^23–27^ benzene dimer (Ben_2_) has two lowest energy structures with almost identical binding energy: A tilted T-shape structure, in which one of the benzene molecules stands almost perpendicular to the other one, and a parallel displaced π-stacked arrangement, which can be obtained by shifting one of the molecules of a cofacial arrangement within its molecular plane.^23,28–30^ In acene crystals, the next neighbor molecules generally arrange in herringbone structures with an edge-to-face orientation and minimized π–π overlap.^11,31^ However, extended π-systems or derivatives with lengthy or bulky substituents frequently show parallel displaced arrangements.^11,32^ Such structures were observed for halogenated tetracenes,^33–35^ rubrene^36,37^ and some of its derivatives,^38–40^ fluorine and alkyl/alkoxy-functionalized tetracenes,^41^ methylthiolated acenes,^35,42,43^ methyltellurated anthracene,^42^ 1,4-pentacenequinone,^44^ pentacenes substituted with bulky alkynyl substituents,^45–50^ certain aryl-functionalized pentacenes^51^ and hexathiapentacene.^52^ A few pentacene derivatives with crossed shape orientation have also been reported.^49,51,53,54^ Accordingly, substitution of π-systems and different morphologies can give rise to molecular crystals with substantially different molecular arrangements. However, to the best of our and other authors^32,55^ knowledge, cofacial orientations have never been found in acene crystals. In accord with these experimental results, a multitude of theoretical investigations revealed that parallel displaced, X- and cross-shaped structures are the energetically most favorable structures of acene dimers.^56–63^

Thus, it seems to be a characteristic feature of neutral, closed-shell π-stacked systems that they strongly avoid eclipsed orientations for like molecules, in which two neighboring π-systems are located in a cofacial structure.^64^ This is commonly explained with repulsive electrostatic interactions due to the quadrupole moments of the π-systems often referred to as the “Hunter-Sanders model” according to the seminal work of these authors from 1990.^74^ However, as very recently pointed out by Wheeler^75^ and by Schramm et al.,^76^ one should differentiate between the original approach of Hunter and Sanders^74^ and a simplified model proposed in 2001 by Hunter et al.^77^ The original Hunter-Sanders model describes intermolecular interactions with exp-6-1 potentials^78^ containing exponential-repulsion, *r* ^–6^-dispersion and *r* ^–1^-Coulombic atom centered terms, as well as quadrupoles which are represented by point charges. The model of Hunter et al.^77^ associates quadrupole moments with total π-systems, making it easier to estimate relative energies of intermolecular arrangements. However, Wheeler^75^ found that predictions of this model are significantly less reliable than those of the Hunter-Sanders potential which, in turn, is at the best only qualitatively correct but often fails completely. We note that the rules for the preferred arrangement of π-systems proposed in the articles by Hunter and Sanders^74^ and by Hunter et al.^77^ are also referred to as the “Hunter-Sanders model”. Especially, the more pictorial of the aforementioned models appeal due to their comprehensibility in the common conceptual framework of molecular interactions and made their way in several textbooks.^79,80^ However, all models based on multipolar electrostatics were frequently shown to provide quantitatively and qualitatively incorrect predictions for π-stacked aggregates.^10,75,76,81–91^

Using Symmetry Adapted Perturbation Theory (SAPT)^92,93^ Carter-Fenk, Herbert and coworkers pointed out that, due to the charge penetration effect,^82–84,91,94–96^ the quadrupole approximation inadequately describes electrostatic interactions, *E*_el_, of closely packed π-stacked systems. Despite being correct at sufficiently large intermolecular separations,^2,3^ multipole expansions break down at shorter distances.^94^ The latter is the case for the quadrupole approximation in the typical π-stacking regime where *E*_el_ is attractive even for cofacial arrangements.^10,76,87–90^ This is in stark contrast to the concepts of Hunter and Sanders^74^ as well as Hunter et al.,^77^ which are based on the idea that this arrangement is electrostatically repulsive. Carter-Fenk and Herbert^87,90^ investigated the interaction energy, *E*_int_, of π-stacked aggregates with fixed inter planar distance as a function of slip-stacked motions of one of the monomers along its molecular plane. In this case, *E*_int_ turned out to be strongly correlated with the van-der-Waals energy, *E*_vdW_, which is the sum of the dispersion energy, *E*_dsp_, and the Pauli repulsion energy that is designated as exchange repulsion energy, *E*_xr_, in the following. The importance of the dispersion and the exchange repulsion energies was rationalized by the fact that for nonpolar π-systems they represent the most attractive and the most repulsive contribution to the interaction energy, respectively. The electrostatic energy was smaller in absolute size and shows no particular features as a function of the parallel displaced arrangement, which is generally observed for similar systems.^57,61,67,76,97^ However, larger changes of the electrostatic energy resembling the intermolecular potential were observed when the inter planar distance was optimized in the course of a slip-stacked motion.^4,97,98^ The resulting effects on *E*_el_ might be explained by a near cancellation of dispersion and exchange repulsion for π-stacked minimum structures.^76,88,97^ In this context, Schramm et al.^76^ argue that *E*_el_ determines the optimum inter planar distance, whereas *E*_vdW_ is responsible for the preference of a slip-stacked arrangement.

In order to gain insight in these interactions, we shall focus in the following on the interaction energy of π-stacked systems with constant inter planar distance. For this case, *E*_int_ is generally similar to the summed dispersion and exchange repulsion energies (see above).^10,76,87–90^ As the dispersion is generally smooth, this indicates that the exchange repulsion has a significant influence on the structure of the intermolecular potential energy surface, as observed e.g., by Ryno et al.^91^ and by Sutton et al.^55^ for acene dimers. Starting from a cofacial π-stacked dimer, they found that a slip-stacked motion of one acene along its long axis gives rise to pronounced oscillations in the exchange repulsion energy. These features were associated with the number of fused rings^91^ and the nodal structure of the highest occupied molecular orbital.^55^

The latter argument is related to the proposal that the exchange repulsion energy is proportional to the squared overlap of the molecular orbitals.^99–103^ Similarly, Bayse and co­workers^98,104–106^ introduced the concept of a “stack bond order”, which identifies favorable stacked arrangements by the difference in the number of occupied bonding and antibonding π-type dimer molecular orbitals. Zhao and Zhang^107^ also highlight the importance of orbital-orbital interactions and they propose a weak intermolecular bond being formed by π- and π*-orbitals. Other works approximated the exchange repulsion with the overlap integral of the monomers’ electron densities.^102,108,109^ Unfortunately, these models are of semiempirical character and, due to a lack of reliable parameters, not generally applicable. So, for a proper understanding of π-stacking, there is a clear lack of a rigorous approach to the exchange repulsion energy that provides qualitative insights and quantitatively correct results.

In this paper, we present a physically grounded, readily understandable, and intuitive explanation for the structures and energetics of π-stacked systems. We consider π-stacked aggregates of benzene (Ben_2_), naphthalene (Nap_2_), anthracene (Ant_2_), tetracene (Tet_2_), and pentacene (Pen_2_) dimers where a constant inter planar distance is chosen for simplicity. We employ SAPT^92,93,110,111^ to demonstrate that *E*_xr_ is decisive for the structure of the interaction energy as a function of the slip-stacked arrangement. The respective features are explained with our recent Molecular Orbital-Pair Contributions to the Exchange repulsion (MOPCE) approach.^86,112^ It partitions *E*_xr_ in terms of orbital-pairs providing the intuitive picture that in a dimer system each occupied orbital on one monomer “repels” each occupied orbital of the other one. We simplify the analysis of the MOPCE terms by grouping them into chemically intuitive σ–σ-, π–σ- and π–π-contributions. The last of which are found to be predominant for the shape of the exchange repulsion energy. We motivate and show that the π–π-MOPCE terms are essentially proportional to the squared overlap integrals of the orbital-pairs. Corroborated by a simple particle-in-a-box model, we will demonstrate that the MOPCE approach can be used to relate the oscillations of the intermolecular potential energy surfaces to the (oscillating) nodal structure of the occupied π-orbitals. This leads to the conclusion that intermolecular potential energy surfaces of π-stacked systems reflect the undulating characteristics of wave functions which are underlying the quantum-mechanical nature of matter.

The article is organized as follows: After describing the theoretical background, we present and discuss SAPT potential energy surfaces of π-stacked acene dimers as a function of slip-stacked displacements. The relevance of *E*_xr_ for these interaction energies is then highlighted and we shall demonstrate that the MOPCE approach properly reproduces this behavior and allows to associate important features of *E*_xr_ to the interaction of electrons in π-orbitals. We shall then focus on the pentacene dimer to establish a detailed understanding and a simple particle-in-a-box model for these π–π-interactions. Finally, we briefly indicate how these findings are related to the other dimers as well as other π-aggregates and present our conclusions.

## Theory

### The Considered Slip-Stacked Arrangements

We consider dimers of benzene (Ben_2_), naphthalene (Nap_2_), anthracene (Ant_2_), tetracene (Tet_2_), and pentacene (Pen_2_). All monomer geometries were optimized at the RI-MP2/aug-cc-pVTZ level of theory using the Turbomole 7.8.1 ^113^ package. *D*_2*h*_ symmetry was invoked for the monomer geometries except for benzene (*D*_6*h*_) so that all atoms are located in the molecular plane (Cartesian coordinates are included in the electronic Supporting Information (ESI) in Tables S1–S5). The dimer structures were created by fixing one of the monomers in the *xy*-plane such that the *x*- and *y*-axes correspond to the long and short axes of the acenes, respectively (see Figure 1). The other monomer (the mobile monomer) was obtained by shifting the fixed monomer by Δ*x*, Δ*y*, and Δ*z* in the respective directions. Hereby, Δ*z* was set to 3.4 Å, a typical inter planar distance of π-stacked aggregates.^32,114–117^ The *x*-shift and *y*-shift values Δ*x* and Δ*y*, respectively, were scanned in intervals of 0.1 Å. Since negative Δ*x*- and/or Δ*y*-shift values lead to symmetry-equivalent structures to the positive ones, only these positive segments are shown in the following.

**Figure 1:**
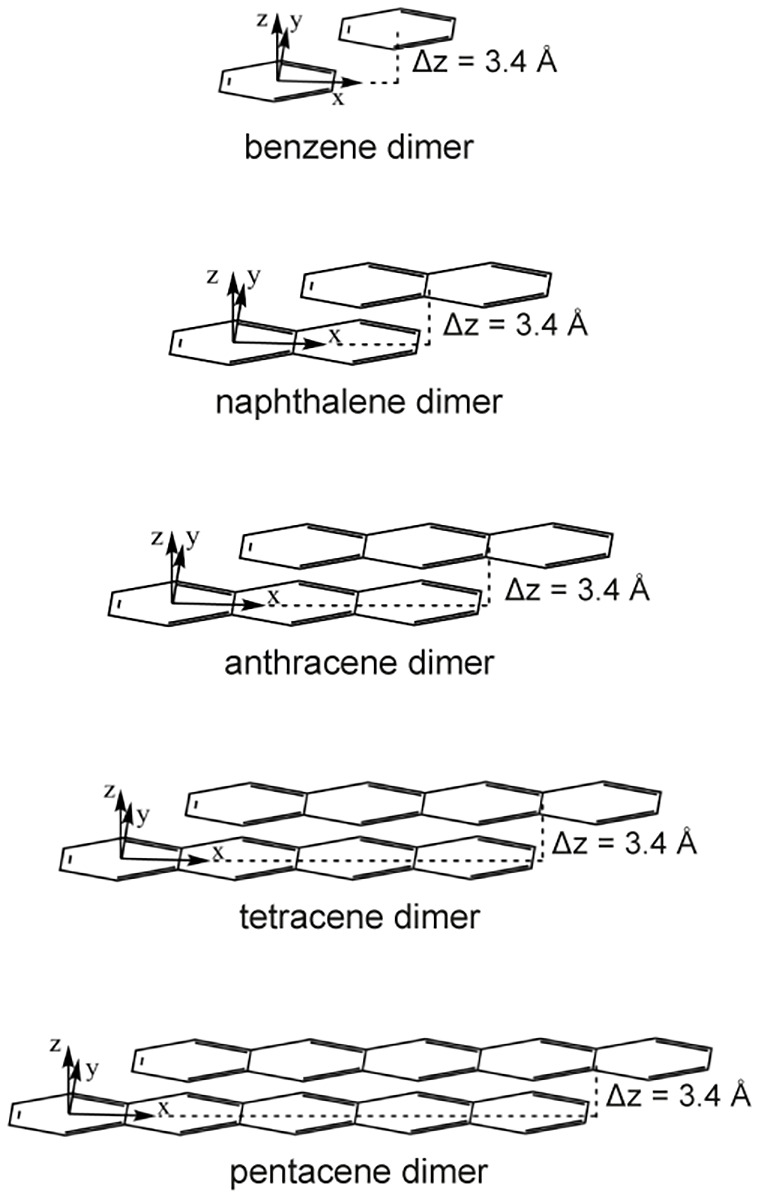
Dimer geometries of parallel displaced acenes with a fixed intermolecular separation of *z* = 3.4 Å and the corresponding coordinate axes.

### Molecular Orbital-Pair Contributions to the Exchange Repulsion (MOPCE)

The central topic of this work is to provide an interpretational basis for the form of the exchange repulsion energy, one of the contributions to the interaction energy all of which are obtained here with the Symmetry Adapted Perturbation Theory (SAPT) approach.^92,118^ Although there are many high level versions of SAPT,^93,110,119,120^ SAPT0 is used here as it is computationally less demanding and sufficiently accurate for the purpose. Its exchange contribution will be referred to as *E*_exch_. In order to understand the exchange repulsion, our recently developed Molecular Orbital-Pair Contributions to the Exchange repulsion (MOPCE) approach^86,112^ is applied. It can be derived in a Heitler-London approach from the energy expectation value of a single Slater determinant containing the occupied molecular orbitals of the isolated molecules and yields essentially the same exchange or Pauli repulsion energy as the *S* ^2^ approximation of SAPT0 ^92,112,121,122^ or the most common energy decomposition analysis methods.^123–125^ Due to different approximations for higher order differential overlap terms as well as the density fitting and counterpoise correction employed in the used SAPT code, the MOPCE exchange repulsion differs slightly from the SAPT exchange *E*_exch_ and is therefore denoted as *E*_xr_. The particularity of the MOPCE method is that it allows the energy to be divided into molecular orbital-pair contributions.

While the detailed derivation of the MOPCE model can be found in Ref 112, a short summary of its most important equations is given in the following. *E*_xr_ can be written as a sum of MOPCE terms

1 $$E_{\mathrm{xr}}=\underset{ab}{\sum }E_{\mathrm{xr}}\left(a,b\right)$$

Here *a* or *a*′ and *b* or *b*′ represent occupied Hartree–Fock spatial orbitals belonging to the isolated systems A and B, respectively. A MOPCE term is given by^112^

2 $$E_{\mathrm{xr}}\left(a,b\right)=-2\left(ab|ba\right)+2S_{ab}\left\{-2\left(a|\mathrm{F̂}|b\right)+\underset{a^{\prime }}{\sum }\left(a|\mathrm{F̂}|a^{\prime }\right)S_{a^{\prime }b}+\underset{b^{\prime }}{\sum }S_{ab^{\prime }}\left(b^{\prime }|\mathrm{F̂}|b\right)+\underset{a^{\prime }b^{\prime }}{\sum }\left[4\left(ab|a^{\prime }b^{\prime }\right)-\left(ab^{\prime }|a^{\prime }b\right)-\left(aa^{\prime }|bb^{\prime }\right)\right]S_{a^{\prime }b^{\prime }}\right\}$$

­(*ab*|*a*′*b*′) is a two electron integral in charge density (Mulliken) notation

3 $$\left(ab|a^{\prime }b^{\prime }\right)=\int \int \psi_{a}^{*}\left({\mathrm{r⃗}}_{1}\right)\psi_{b}\left({\mathrm{r⃗}}_{1}\right)\frac{1}{r_{12}}\psi_{a^{\prime }}^{*}\left({\mathrm{r⃗}}_{2}\right)\psi_{b^{\prime }}\left({\mathrm{r⃗}}_{2}\right)d{\mathrm{r⃗}}_{1}d{\mathrm{r⃗}}_{2}$$

while *F̂* = *T̂* + *V̂*_*A*_ + 2*Ĵ*_*A*_ – *K̂*_*A*_ + *V̂*_*B*_ + 2*Ĵ*_*B*_ – *K̂*_*B*_ is a Fock operator of the dimer system containing the kinetic energy operator *T̂*, the electron-nuclei attraction, *V̂*_*X*_, Coulomb, *Ĵ*_*X*_, and exchange operators, *K̂*_*X*_, of the systems *X* = *A* and *B*, respectively. Note, that *a* or *b* represent occupied (rather than virtual) orbitals and that an index *a* in a sum is meant to run over all occupied orbitals of system *A*. As also shown recently,^112^ the MOPCE terms in eq 2 can be rewritten to

4 $$E_{\mathrm{xr}}\left(a,b\right)=E_{\mathrm{xi}}\left(a,b\right)+E_{\mathrm{xr}2}\left(a,b\right)+E_{\mathrm{xr}3}\left(a,b\right)+E_{\mathrm{xr}4}\left(a,b\right)+E_{\mathrm{xrb}}\left(a,b\right)$$

where

5 $$E_{\mathrm{xi}}\left(a,b\right)=-2\left(ab|ba\right)$$

is the exchange integral contribution. For canonical monomer orbitals, the two-index term which gives rise to the dominant repulsive contribution to *E*_xr_ can be written as

6 $$E_{\mathrm{xr}2}\left(a,b\right)=-2\left(\epsilon_{a}+\epsilon_{b}\right)S_{ab}^{2}+2S_{ab}T_{ab}$$

where the orbital energy ϵ_*a*_ is the eigenvalue of orbital *a* to the Fock operator of monomer A which is defined as *F̂*_*A*_ = *T̂* + *V̂*_*A*_ + 2*Ĵ*_*A*_ – *K̂*_*A*_. An alternative expression of the two-index MOPCE term is

7 $$E_{\mathrm{xr}2}\left(a,b\right)=-2S_{ab}\left(b|{\mathrm{V̂}}_{A}|a\right)-2S_{ba}\left(a|{\mathrm{V̂}}_{B}|b\right)$$

where e.g., ${\mathrm{V̂}}_{A}={\mathrm{V̂}}_{A}+2{Ĵ}_{A}-{\mathrm{K̂}}_{A}$ is the potential energy operator of an electron in system A. Both representations are useful to rationalize the MOPCE terms as a function of the slip-stack motions: eq 6 suggests a similarity of *E*_xr_(*a*,*b*) with the squared overlap matrix element $S_{ab}^{2}$ , which is actually observed below, while eq 7 provides insight into the physical nature of the exchange repulsion energy. The latter can be interpreted as a consequence of the Pauli principle which causes that two electrons with the same spin are not allowed to be at the same place. The ket-part of the first term in eq 7, *V̂*_*A*_ + 2*Ĵ*_*A*_ – *K̂*_*A*_|*a*), is the orbital *a* multiplied with its effective potential energy in the system A within the Hartree–Fock representation. Thus, the first term in eq 7 can be interpreted as the amount of the potential energy of orbital *a* that is not available to system A due to the Pauli principle. This means, the electron of orbital *a* must not be located at a position that is already occupied by a same-spin electron from orbital *b*.^112^ The second term is therefore the potential energy contribution of the electrons in orbital *b*, which must not be counted, since it is Pauli forbidden to occupy the corresponding position due to the electrons in orbital *a*.

The three-index term can be written as

8 $$E_{\mathrm{xr}3}\left(a,b\right)=2S_{ab}\left[\underset{a^{\prime }}{\sum }\left(a|{\mathrm{F̂}}_{B}-\mathrm{T̂}|a^{\prime }\right)S_{a^{\prime }b}+\underset{b^{\prime }}{\sum }\left(b|{\mathrm{F̂}}_{A}-\mathrm{T̂}|b^{\prime }\right)S_{b^{\prime }a}\right]$$

It is generally small, as is also the basis set error term

9 $$E_{\mathrm{xrb}}\left(a,b\right)=2S_{ab}\left[-\left(a|F_{A}|b\right)+\underset{a^{\prime }}{\sum }\left(a|F_{A}|a^{\prime }\right)S_{a^{\prime }b}-\left(b|F_{B}|a\right)+\underset{b^{\prime }}{\sum }\left(b|F_{B}|b^{\prime }\right)S_{b^{\prime }a}\right]$$

The latter, however, vanishes for complete basis sets or if a counterpoise correction^126^ basis is applied.^112,127^ The four-index term

10 $$E_{\mathrm{xr}4}\left(a,b\right)=2S_{ab}\underset{a^{\prime }b^{\prime }}{\sum }\left[4\left(ab|a^{\prime }b^{\prime }\right)-\left(ab^{\prime }|a^{\prime }b\right)-\left(aa^{\prime }|bb^{\prime }\right)\right]S_{a^{\prime }b^{\prime }}$$

generally turns out to be negative.

As in eq 1, the sum over all orbitals of the *E*_xr2_(*a*,*b*) terms provides the two-index term of the exchange repulsion energy

11 $$E_{\mathrm{xr}2}=\underset{a,b}{\sum }E_{\mathrm{xr}2}\left(a,b\right)$$

The same holds for the three-index, *E*_xr3_, and four-index, *E*_xr4_, contributions as well as the exchange integral term, *E*_xi_. In our recent publication^112^ these quantities were found to be roughly proportional to each other and to the total exchange repulsion energy which is an important argument for the idea that the three- and four-index terms can be combined into orbital-pair contributions.^112^

Equation 2 reveals, that any orbital-pair contribution consists of two parts, the exchange integral contribution, *E*_xi_(*a*,*b*) and the repulsive remainder,

12 $$E_{\mathrm{rep}}\left(a,b\right)=E_{\mathrm{xr}}\left(a,b\right)-E_{\mathrm{xi}}\left(a,b\right)$$

It can be associated with the (Pauli) repulsion that is due to the electron pairs occupying orbitals *a* and *b*. It is given by the term with the curly brackets in eq 2 and thus vanishes if the overlap integral $S_{ab}^{2}$ is zero. In this case, *E*_xr_(*a*,*b*) equals −2­(*ab*|*ba*), which must be negative as the respective exchange integral (*ab*|*ba*) is always positive.^128^

Empirical considerations of the exchange repulsion energy concluded that it should be roughly proportional to the square of the overlap between interacting atomic orbitals divided by a power of the distance, *R*, between the next neighbor atoms *E*_xr_ ≈ *S* ^2^/*R* ^*n*^.^103,129,130^ For the slip-stacked arrangements with constant inter planar distance considered here, the distance of a given atom on one molecule to its next neighbor atom on the other molecule is essentially constant. As we shall show below, the MOPCE term *E*_xr_(*a*,*b*) is in good approximation proportional to the squared overlap matrix elements of the molecular orbitals which is thus designated as $S^{2}\left(a,b\right)\equiv S_{ab}^{2}$ .

### Particle-in-a-Box Model

The overlap matrix elements *S*(*a*,*b*) are strongly oscillating functions of the *x*- and *y*-shifts if the orbitals themselves oscillate as a function of the *x*- and *y*-coordinates. This behavior can be described with a very simple model using particle-in-a-box wave functions indicated in Figure 2. If we consider the *x*-shift, we approximate the π-orbitals by one-dimensional wave functions of the particle-in-a-box model where the box length, *L*, corresponds to the extension of the π-system in *x*-direction. The box of system A ranges from 0 to *L* and within this range the *n*th wave function is given by ψ_*A*,*n*_(*x*) = (2/*L*)^1/2^ sin­(*π n x*/*L*), while it is zero for *x* ≥ *L* and *x* ≤ 0. In the interval between Δ*x* and *L* + Δ*x* the wave functions of system B are ψ_*B*,*n*_ = (2/*L*)^1/2^ sin­(*π n* (*x* - Δ*x*)/*L*) and zero elsewhere. This allows to approximate the overlap integral between the MOs of two π-systems as

13 $$S_{B}\left(n,m\right)=\int_{\Delta x}^{L}\psi_{A,n}\left(x\right)\psi_{B,m}\left(x\right)dx$$

14 $$=\{\begin{array}{ll} \frac{2}{\pi }\frac{{\left(-1\right)}^{n+m}n\sin\left(\pi ma\right)-m\sin\left(\pi na\right)}{n^{2}-m^{2}} & \mathrm{if}\;n\neq m\\ \left(1-a\right)\cos\left(\pi na\right)+\frac{\sin\left(\pi na\right)}{n\pi } & \mathrm{if}\;n=m, \end{array}$$

where *a* = Δ*x*/*L*. The same formalism works in a straightforward manner for the Δ*y*-motion that is also discussed below and can easily be extended to other slip-stacked arrangements.

**Figure 2:**
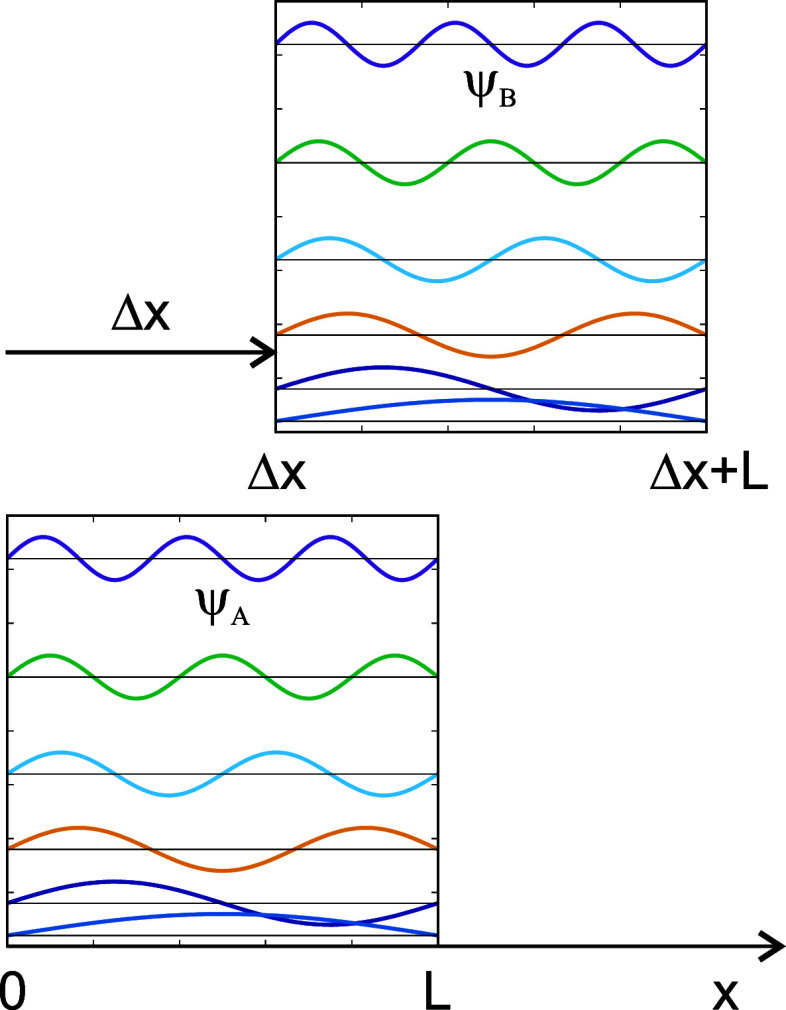
Particle-in-a-box model for the orbital overlap. Δ*x* indicates the shift between the boxes.

## Computational Details

Symmetry Adapted Perturbation Theory (SAPT) calculations were conducted for each dimer geometry at the SAPT0/jun-cc-pVDZ^131^ level of theory using the Psi4 1.7 program package^132^ to obtain potential energy surfaces (PES) for the interaction energy *E*_int_ as well as the electrostatic, *E*_el_, induction, *E*_ind_, dispersion, *E*_dsp_, and the exchange, *E*_exch_, energy contribution according to the energy partitioning

15 $$E_{\mathrm{int}}=E_{\mathrm{el}}+E_{\mathrm{ind}}+E_{\mathrm{dsp}}+E_{\mathrm{exch}}$$

For SAPT0, these terms are defined as follows:^92,118^ $E_{\mathrm{el}}=E_{\mathrm{elst}}^{\left(10\right)}$ , $E_{\mathrm{ind}}=E_{\mathrm{ind},\mathrm{resp}}^{\left(20\right)}+E_{\mathrm{exch‐ind},\mathrm{resp}}^{\left(20\right)}+\delta E_{\mathrm{ind},\mathrm{resp}}^{\left(\mathrm{HF}\right)}$ , $E_{\mathrm{dsp}}=E_{\mathrm{disp}}^{\left(20\right)}+E_{\mathrm{exch‐disp}}^{\left(20\right)}$ , and $E_{\mathrm{exch}}=E_{\mathrm{exch}}^{\left(10\right)}$ . To check the reliability of these results, some characteristic points were recalculated at the levels of theory that were proposed by Parker et al.^93^ as gold (SAPT2+(3)­δMP2/aug-cc-pVTZ), silver (SAPT2+/aug-cc-pVDZ), and bronze (sSAPT0/jun-cc-pVDZ) standard of SAPT. The results shown in Tables S6–S8 in the ESI underline that the SAPT0/jun-cc-pVDZ results provide a realistic estimate of the qualitative features of the potential energy curves.

MOPCE energies were obtained with the jun-cc-pVDZ basis set with the Hartree–Fock orbitals of the individual monomers using ″wavels″, a program package developed in our group.^86,112,133^ Potential energy surfaces were visualized and analyzed using matlab R2023a^134^ as well as pandas 1.1.5 ^135,136^ numpy 1.17.3 ^137^ and matplotlib 3.3.4.^138,139^ Some figures were generated with gnuplot 5.4.^140^ and CorelDraw 25.2.1.313.^141^ Molecular orbital representations were generated with MOLDEN 5.9.^142^

Dimer geometries of particular interest are denoted as follows: The dimer geometrical structures with Δ*x* = Δ*y* = 0.0 Å are the cofacial arrangements, also called eclipsed or sandwich structures. Minima of the interaction energy PES along one axis (Δ*x* or Δ*y*) are designated as *x*-stacked and *y*-stacked minima. True minima on the two-dimensional surface where the mobile monomer is shifted in *x*- and *y*-direction are referred to as slip-stacked minima.

## Results and Discussion

### SAPT Results for the Intermolecular Potentials

Contour plots of the SAPT interaction and exchange energies as a function of the *x*- and *y*-shifts are shown in Figure 3. SAPT0/jun-cc-pVDZ energies for several characteristic points on these surfaces are collected in Table 1. As already shown previously,^93^ interaction energies of SAPT0/jun-cc-pVDZ and the gold standard of SAPT deviate by about 10%. However, in this work we rather focus on relative energies which are in good agreement between all levels of theory. Since silver or gold standard of SAPT is computationally very demanding for the larger acene dimers, only the SAPT0/jun-cc-pVDZ results are considered in the following. All systems show a distinct maximum of the exchange repulsion energy at the cofacial orientation, which is in line with the observation, that acenes avoid this orientation in crystals. Dimers of the larger acenes exhibit a global minimum of the interaction energy *E*_int_ in a slip-stacked orientation with about Δ*x* = 1.3 Å and Δ*y* = 1.0 Å, while two symmetry equivalent global minima with an interaction energy of −12.01 kJ mol^–1^ exist for Ben_2_. One along the *y*-axis at Δ*y* = 1.81 Å and the second one at Δ*x* = 1.57 Å and Δ*y* = 0.90 Å. These minima are very shallow, as the interaction energy at the global minima is only 0.35 kJ mol^–1^ lower than at the saddle points in between. The latter are located at Δ*x* = 1.74 Å, Δ*y* = 0.0 Å and Δ*x* = 0.87 Å, Δ*y* = 1.50 Å. Due to this shape of the Ben_2_ potential, it would probably be more appropriate to speak of a minimum valley in this case.

**Figure 3:**
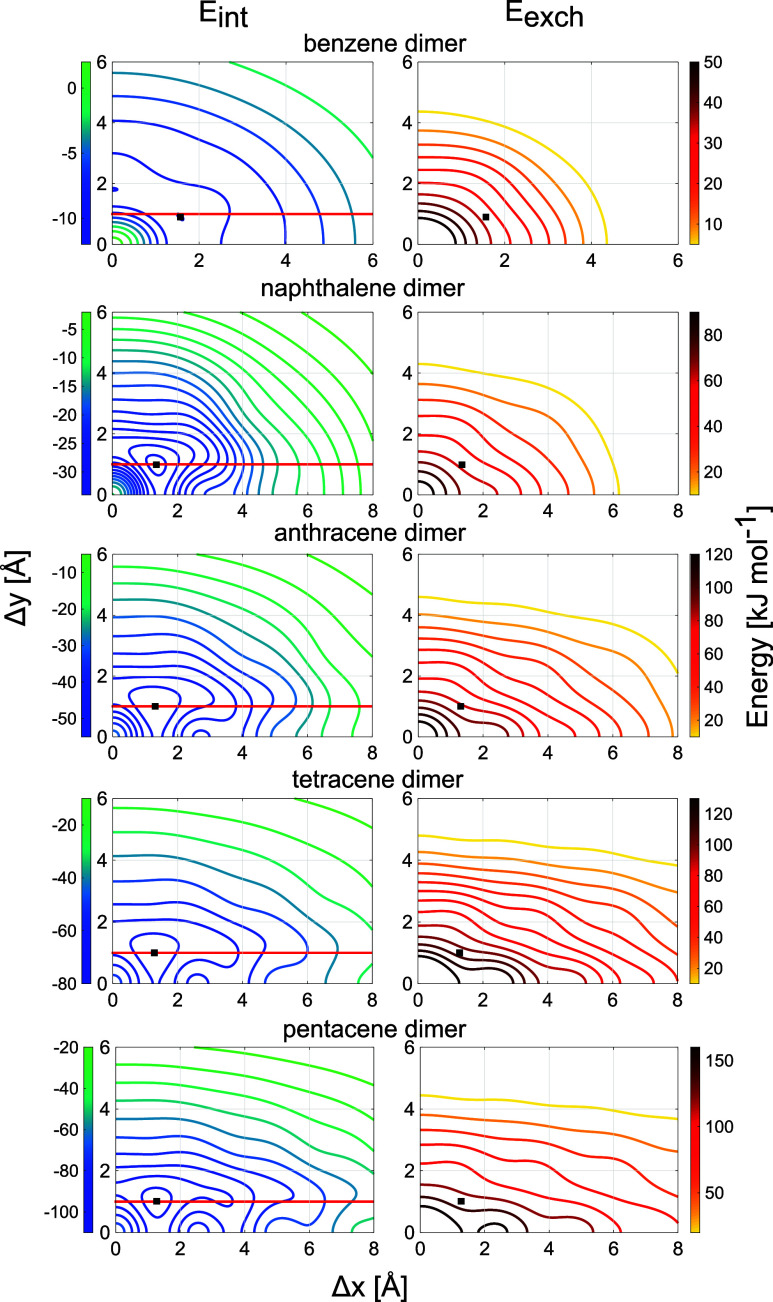
Contour plots of the interaction energy *E*_int_ (left) and the exchange energy *E*_exch_ (right) at the SAPT0/jun-cc-pVDZ level as a function of the slip-stacked arrangement (Δ*x*, Δ*y*) for the investigated acene dimer systems. Positions of the absolute minium of the interaction energy are indicated with black squares.

**Table 1:** Interaction Energy and its Energy Contributions at the Cofacial Arrangements, the *X*- and *Y*-Stacked Minima, as Well as the Slip-Stacked Minima of the Acene Dimers for a Fixed Intermolecular Distance of Δ*z* = 3.4 Å^a^

System	Δ*x* [Å]	Δ*y* [Å]	*E*_int_	*E*_el_	*E*_ind_	*E*_dsp_	*E*_exch_	Position on PES
Ben_2_	0.00	0.00	2.84	–14.86	–2.41	–42.09	62.21	cofacial
	1.57	0.90	–12.01	–10.11	–3.51	–31.10	32.71	slip-stacked
	1.74	0.00	–11.78	–10.49	–3.77	–31.81	34.29	*x*-stacked
	0.00	1.81	–12.01	–10.11	–3.51	–31.10	32.71	*y*-stacked
Nap_2_	0.00	0.00	–11.97	–25.07	–2.99	–79.82	95.91	cofacial
	1.35	0.99	–34.65	–19.53	–6.02	–66.72	57.62	slip-stacked
	1.46	0.00	–32.42	–21.53	–6.46	–71.45	67.02	*x*-stacked
	0.00	1.53	–31.22	–18.56	–5.12	–65.13	57.58	*y*-stacked
Ant_2_	0.00	0.00	–28.36	–35.44	–3.56	–119.80	130.43	cofacial
	1.31	1.00	–59.97	–28.78	–8.71	–104.12	81.63	slip-stacked
	1.37	0.00	–55.68	–32.06	–9.36	–112.53	98.27	*x*-stacked
	0.00	1.46	–53.29	–27.02	–6.43	–100.55	80.71	*y*-stacked
Tet_2_	0.00	0.00	–46.07	–45.89	–4.13	–161.18	165.13	cofacial
	1.28	1.00	–86.78	–38.08	–11.64	–142.96	105.90	slip-stacked
	1.33	0.00	–80.40	–42.40	–12.38	–154.64	129.02	*x*-stacked
	0.00	1.42	–76.83	–35.60	–7.72	–137.26	103.74	*y*-stacked
Pen_2_	0.00	0.00	–64.71	–56.44	–4.69	–203.57	200.00	cofacial
	1.27	1.01	–114.56	–47.30	–14.77	–182.22	129.72	slip-stacked
	1.31	0.00	–106.02	–52.71	–15.50	–197.48	159.68	*x*-stacked
	0.00	1.40	–101.40	–44.21	–9.06	–174.58	126.45	*y*-stacked

a The SAPT0/jun-cc-pVDZ energies are given in kJ mol^–1^.

For the other acenes, the *x*- and *y*-stacked minima represent saddle points of the interaction energy *E*_int_. The Δ*x*-values of the *x*-stacked minima decrease with increasing system size. It amounts to about Δ*x* = 1.7 Å for Ben_2_, 1.4 Å for Nap_2_ and Ant_2_, as well as 1.3 Å for Tet_2_ and Pen_2_ (see Table 1). The interaction energies at these structures are only slightly higher than at the global minimum by 2.2 kJ mol^–1^ for Nap_2_, 4.3 kJ mol^–1^ for Ant_2_, 6.4 kJ mol^–1^ for Tet_2_, and 8.5 kJ mol^–1^ for Pen_2_. The cofacial orientation is, respectively, 23 kJ mol^–1^, 32 kJ mol^–1^, 41 kJ mol^–1^, and 50kJ mol^–1^ less favorable than the slip-stacked minimum.

Grimme^143^ observed nonlinear increase of *E*_dsp_ with the size of aromatic hydrocarbons compared to linear increase in saturated hydrocarbons and concluded that the dispersion interaction is decisive for the strength of π–π-interactions. He further ascribes the parallel displacement in dimers of aromatic hydrocarbons to a reduction of electrostatic and Pauli repulsion. Other authors^57,88^ compared similar systems, but they stress the balance between dispersion and exchange repulsion, which leaves the smaller electrostatic and induction contributions for rationalizing differences in stability. Carter-Fenk, Herbert and coworkers^10,76,87–90^ view the van-der-Waals interactions, i.e., the sum of dispersion and exchange repulsion, as driving force of the slip-stacking phenomenon. In contrast, other studies^4,74,77,97^ associate this role to the electrostatic interaction, but their results are either based on potential energy curves at optimized inter planar distances or on methods neglecting charge penetration.

Our results confirm that *E*_dsp_ and *E*_el_ are the largest attractive contributions. However, as already noted by Carter-Fenk and Herbert,^87^ they favor the cofacial arrangement, while the slip-stacked minimum structure is clearly due to *E*_exch_. For Pen_2_, for example, this can be seen from the changes of the interaction energy contributions between the slip-stacked minimum and the cofacial orientation: They amount to 9kJ mol^–1^ for *E*_el_, −10kJ mol^–1^ for *E*_ind_, 21 kJ mol^–1^ for *E*_dsp_ and −70 kJ mol^–1^ for *E*_exch_. The interaction energy lowering of −50 kJ mol^–1^ is, thus, mostly due to decrease of *E*_exch_. The same trend is observed for the other acene dimer systems. Thus, the preference of the attractive contributions for the cofacial orientation is overcompensated by the particularly high repulsion in this arrangement. We conclude that the dispersion takes a prominent role in π-interactions, but cannot explain the characteristic avoidance of cofacial arrangements of the acene dimers.

For the investigated acene dimers, the induction contributions, *E*_ind_, are more negative for the slip-stacked minima than for the cofacial ones but for the *x*-stacked minima, they are even lower (see Table 1 and Figure S3 in the ESI). However, the magnitude of the induction is relatively low (less than 12% of the dispersion contribution) and, therefore, cannot explain the minima found on the PES. The exchange repulsion is of the same order of magnitude as the dispersion and has a distinctly anisotropic structure. The latter can be deduced from the corresponding contour plots of *E*_exch_ in the right column of Figure 3 but is more apparent from the surface plots in Figure S5 in the ESI and from the cuts through the energy surfaces along the *x*- and *y*-axes presented in Figure 4. As we shall show, this anisotropic structure has crucial influence on the interaction energy. The corresponding contour plots of *E*_int_ are given in the left column of Figure 3 (see Figures S1–S5 in the ESI for 2D plots of *E*_int_ and its energy contributions for all systems).

**Figure 4:**
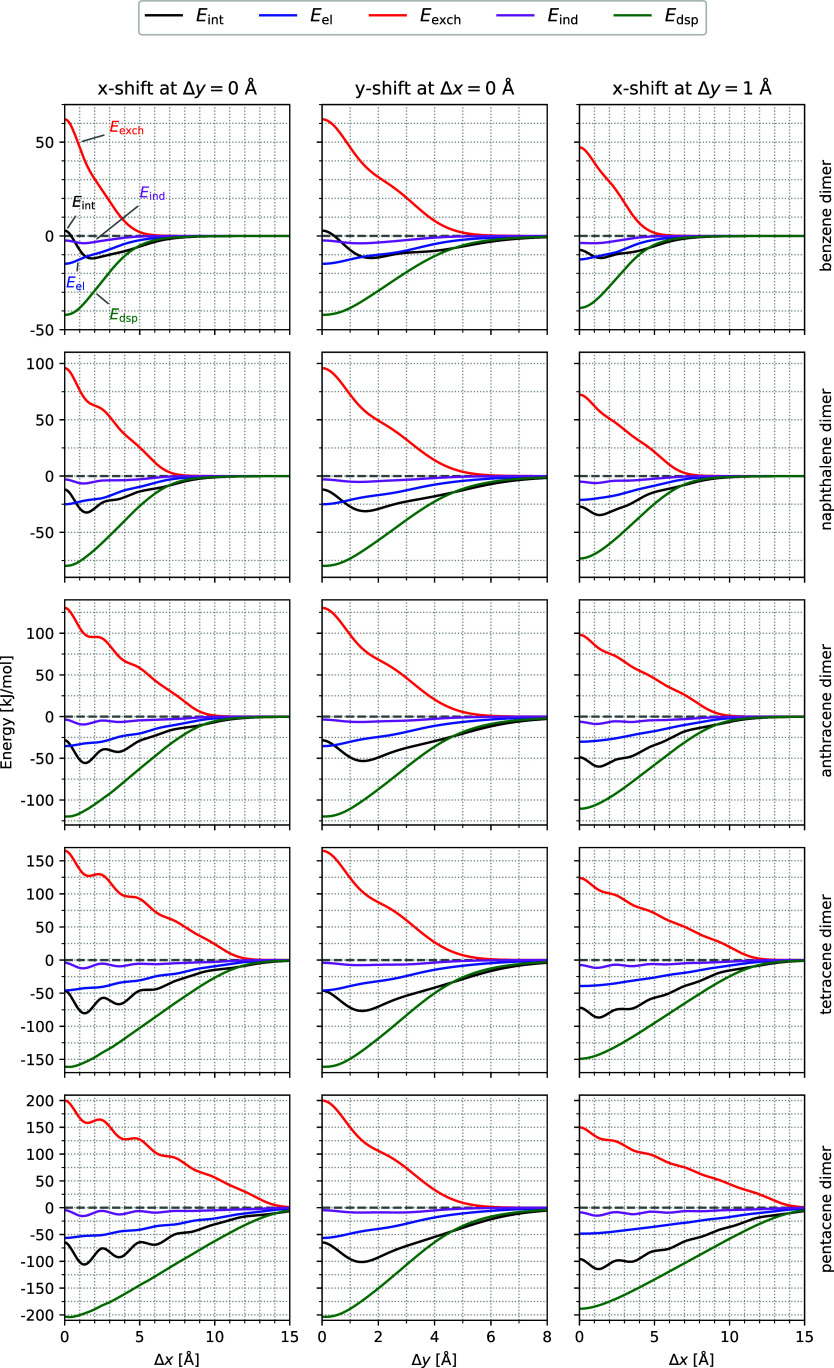
Potential energy curves of the SAPT0/jun-cc-pVDZ interaction energy *E*_int_ and its energy contributions for (from above to below) Ben_2_, Nap_2_, Ant_2_, Tet_2_, and Pen_2_ as a function of Δ*x* for Δ*y* = 0 Å (left), Δ*y* for Δ*x* = 0 (middle), and Δ*x* for Δ*y* = 1 Å (right). The inter planar distance is fixed to Δ*z* = 3.4 Å.

For a better visualization and understanding of the potential energy surfaces characteristics, three cuts through the PES are investigated. Two of them are starting from the cofacial orientation along the *x*- and *y*-axes (we refer to these motions as Δ*x* and Δ*y*) and the third one runs along the *x*-axis and essentially through the slip-stacked minimum (designated as Δ*x* with Δ*y* = 1 Å). It is indicated in Figure 3 with red lines. The interaction energy *E*_int_ and its SAPT energy contributions are plotted along these cuts in Figure 4.

The cuts along the *x*-axis (left column in Figure 4) are in line with previous results by Ryno et al.^91^ *E*_int_ and *E*_exch_ show an oscillating behavior, which is most obvious for Pen_2_. There, three local minima are found for *E*_int_ at Δ*x* = 1.31 Å, 3.63 Å and 5.96 Å. For *E*_exch_ we find two minima at Δ*x* = 1.52 Å and 4.06 Å and a shoulder at about 6 Å. Due to the attractive energy contributions, the minima of the interaction energy along the *x*-axis are found at smaller Δ*x*-values than the minima of *E*_exch_. We observe that with increasing number of rings of the acenes, the shoulders of *E*_exch_ become more pronounced and more numerous. The same holds for the minima in the interaction energy *E*_int_. These features are rather pronounced for small Δ*x*-values and fade away for large Δ*x*.

The middle column of Figure 4 shows that for all acenes, a shift along the *y*-axis gives rise to a single minimum of the interaction energy at about Δ*y* = 1.5 Å. Here again, the dispersion is the most attractive contribution to the interaction energy. Starting from the cofacial arrangement (Δ*y* = 0 Å), it increases gently with increasing Δ*y*. *E*_el_ is about four times smaller and comparably smooth while *E*_ind_ is almost negligible. The minimum in the interaction energy is due to the sharp decrease of *E*_exch_ from Δ*y* = 0 to about 1.5 Å. Thus, the existence and location of the minimum is again associated with features in *E*_exch_.

A shift along Δ*x* for Δ*y* = 1 Å passes through the slip-stacked minima of the acene dimers (see the red line in the left column of Figure 3). The results, presented in the right column of Figure 4, reveal reduced oscillations of *E*_exch_ which cause minima in *E*_int_ that are less pronounced but otherwise comparable to the Δ*y* = 0 Å *x*-shift. Interestingly, a careful comparison of the *E*_int_ curves shows that the oscillations for large Δ*x*-values can be still observed for Δ*y* = 1 Å while such structures are weaker for Δ*y* = 0 Å. This is better visible in Figures S6 and S7 in the ESI, where these energies and their first and second derivatives are presented as a function of Δ*x*. The striking similarity between the first and even more the second derivatives of the corresponding energy curves underline that the oscillations in *E*_int_ are caused by the structure in the exchange repulsion energy. These figures show that the *x*-shift for Δ*y* = 0 Å shows strong oscillations for small Δ*x* values which fade out almost completely for Δ*x* greater than about 9 Å, while for *x*-shift with Δ*y* = 1 Å the oscillations start weaker at small Δ*x*-values, but decay more slowly, so that for Δ*x* = 12 Å a fluctuation can still be identified.

Summing up, the SAPT results suggest that the exchange repulsion is essential to understand the form of the PES. In particular, we observe strong oscillations of *E*_exch_ for a shift along the *x*-axis with Δ*y* = 0 Å, which appear to be similar but weakened for Δ*y* = 1 Å, while a shift along the *y*-axis gives rise to a single shoulder in *E*_exch_. In the following, we shall show that these patterns can be rationalized with the MOPCE terms introduced above.

### π–π, π–σ, and σ–σ Contributions to *E*_xr_

So far we considered *E*_exch_, the exchange repulsion energy contribution as obtained from SAPT0. In the following, we shall consider *E*_xr_, the corresponding property from the MOPCE approach which provides important insight into the nature of the exchange repulsion. We shall show this below for cuts through the *E*_xr_ surface along the *x*- and *y*-axes as well as for an *x*-shift with Δ*y* = 1 Å as presented in Figure 4.

Figure 5 shows *E*_xr_ for these cuts which reproduces all features discussed above for the SAPT0 exchange energy.

**Figure 5:**
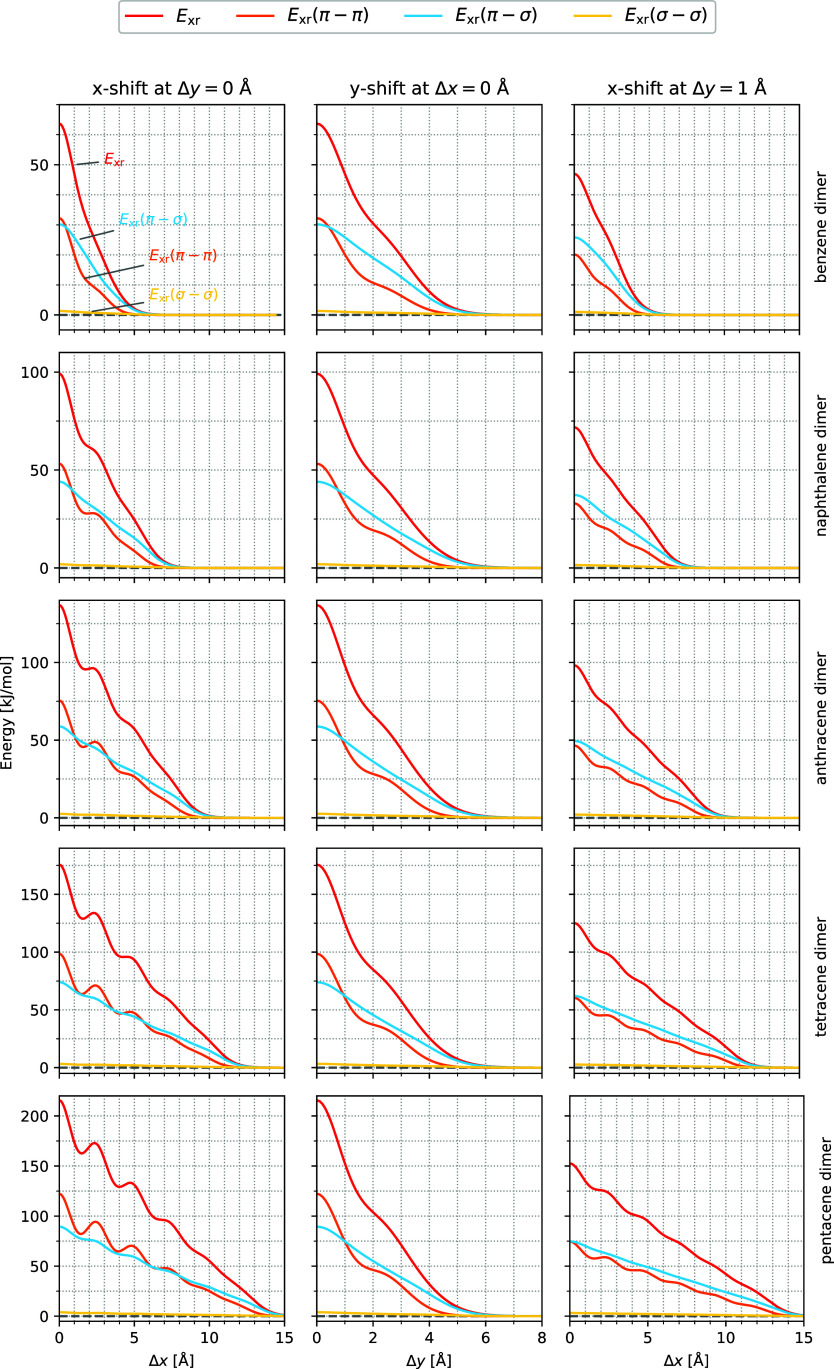
The total exchange repulsion energy *E*_xr_ and contributions due to π- and σ-orbitals *E*_xr_(π–π), *E*_xr_(π–σ) and *E*_xr_(σ–σ) are shown for Ben_2_, Nap_2_, Ant_2_, Tet_2_, and Pen_2_ (from above to below) as a function of Δ*x* with Δ*y* = 0 Å (left), Δ*y* for Δ*x* = 0 (middle) and Δ*x* for Δ*y* = 1 Å (right).

The oscillations of *E*_xr_ can be explained by the orbital-pair contributions *E*_xr_(*a*,*b*) introduced in eq 2. However, as the number of these contributions increases rapidly with the system size, the analysis of each individual contribution is unfeasible even for the smallest system, Ben_2_, which has 441 orbital-pair contributions to the exchange repulsion. 5329 orbital-pair contributions can be identified for Pen_2_. Thus, we define classes of orbital-pair contributions in an obvious way by distinguishing between π- and σ-type monomer orbitals. This leads to π–π, π–σ, and σ–σ contributions

16 $$E_{\mathrm{xr}}\left(\pi -\pi \right)=\underset{a\in \pi ,b\in \pi }{\sum }E_{\mathrm{xr}}\left(a,b\right)$$

17 $$E_{\mathrm{xr}}\left(\pi -\sigma \right)=\underset{a\in \pi ,b\in \sigma }{\sum }E_{\mathrm{xr}}\left(a,b\right)+\underset{a\in \sigma ,b\in \pi }{\sum }E_{\mathrm{xr}}\left(a,b\right)$$

18 $$E_{\mathrm{xr}}\left(\sigma -\sigma \right)=\underset{a\in \sigma ,b\in \sigma }{\sum }E_{\mathrm{xr}}\left(a,b\right)$$

Here *E*_xr_(π–σ) represents the sum of the MOPCE contributions *E*_xr_(*a*,*b*) where *a* is a π-orbital and *b* of σ-type or vice versa. These terms are shown in Figure 5 for the displacements along the *x*- and *y*-axes as well as along Δ*x* for Δ*y* = 1 Å for the considered acene dimers.

Note that for Pen_2_, *E*_xr_(σ–σ) consists of 3844 orbital-pair contributions, *E*_xr_(π–σ) of 1364, and *E*_xr_(π–π) of 121. Thus, although containing the largest number of contributions, *E*_xr_(σ–σ) accounts for only 2% of the exchange repulsion in the cofacial orientation. Similar ratios are found for the other structures. Due to their low values, the *E*_xr_(σ–σ) contributions are insignificant for *E*_xr_, and can be neglected. For the curves in Figure 5, *E*_xr_(π–π) is slightly larger than *E*_xr_(π–σ) in the cofacial arrangement (Δ*x* = 0 Å), and generally a bit smaller for the *x*- and *y*-stacked and slip-stacked minima. As there are about ten times as many orbital-pair contributions between π- and σ-orbitals than between π-orbitals, the former are generally much smaller than the latter. We note that *E*_xr_(π–σ) has a maximum for the cofacial arrangement and decreases smoothly with increasing displacement, showing no particular features. In contrast, *E*_xr_(π–π) stands out with pronounced oscillations which give rise to the dominant features of the total exchange repulsion *E*_xr_ curve. Thus, *E*_xr_(π–π) appears to be central for the structure of *E*_xr_.

It is worth to note that exchange repulsion in π-stacked systems is with about equal amounts due to π–π and π–σ interactions, while *E*_xr_(σ–σ) is almost negligible. As π-orbitals vanish in the molecular planes, they extend further above and below the planes than σ-orbitals. This rationalizes the dominance of *E*_xr_(π–π) and *E*_xr_(π–σ) for the exchange repulsion.

### π–π Contributions to *E*_xr_ of the Pentacene Dimer

A closer look at the orbital-pair contributions between π-orbitals allows to rationalize the observed *E*_xr_ structure. In the following we consider first the *x*-shift for the Pen_2_ system which shows the most striking structures in *E*_xr_. The behavior of the other systems can be understood on that basis.

Figure 6 shows the occupied π-orbitals of the pentacene monomer. Six of these orbitals are symmetric (S) with respect to the σ_*xz*_ mirror plane and five of them are antisymmetric (A). The orbitals are designated in the following as 1S, 2S, etc. (1A, 2A, etc.) in increasing order of their energy (see Figure 6). The Mulliken symbols, orbital energies, and the *n*S/A designations of the orbitals are collected in Table 2. As can be seen from Figure 6, the *n*S and *n*A orbitals have *n* – 1 nodal surfaces along the *x*-axis.

**Figure 6:**
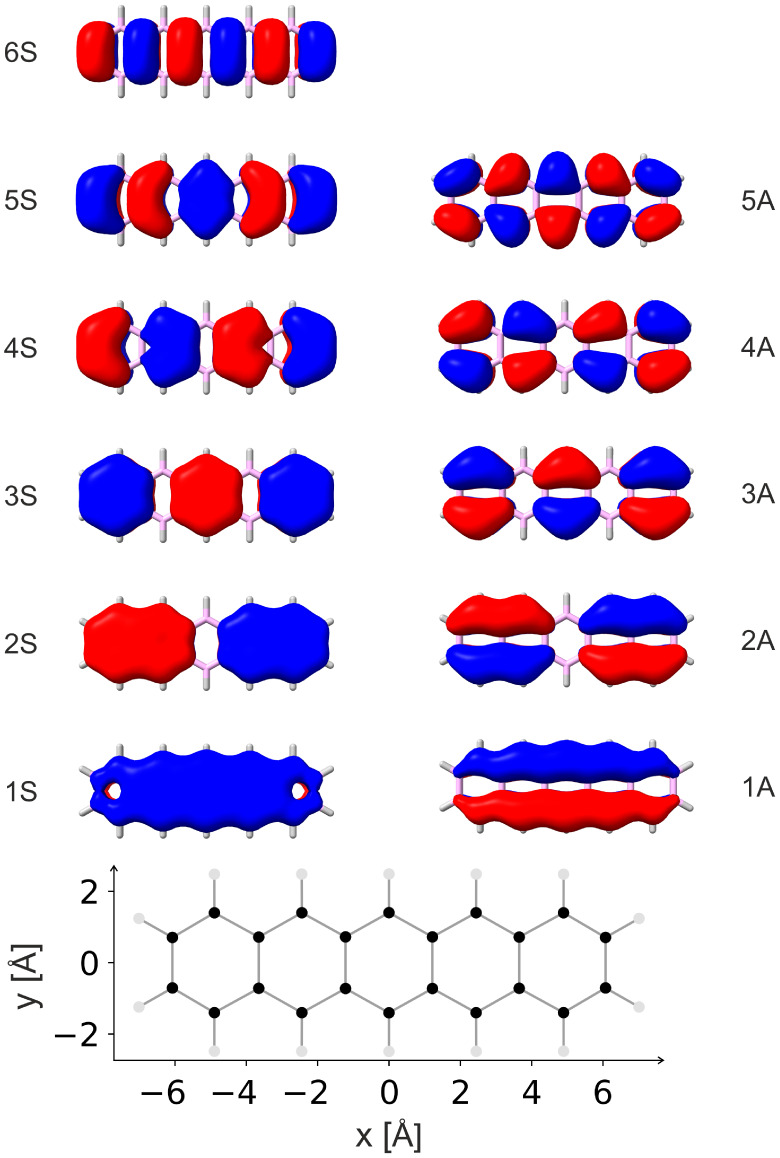
The occupied π-orbitals of pentacene (upper part) and a ball and stick model indicating the size of this molecule (lower part).

**Table 2:** Numbers of the Occupied Hartree-Fock *π*-Orbitals of Pentacene, Their Designation as Symmetric (*n*S) or Antisymmetric (*n*A) with Respect to the *σ_xz_* Plane, the Mulliken Symbols, and Their Orbital Energies, *ϵ*

No.	*n*S/A	Mulliken symbol	ϵ [E_h_]
73	5A	3b_3g_	–0.2185
72	4A	2a_u_	–0.2922
71	6S	3b_2g_	–0.3036
70	3A	2b_3g_	–0.3525
69	5S	3b_1u_	–0.3724
68	2A	1a_u_	–0.3973
67	1A	1b_3g_	–0.4264
66	4S	2b_2g_	–0.4403
62	3S	2b_1u_	–0.4952
58	2S	1b_2g_	–0.5358
57	1S	1b_1u_	–0.5606

#### X-Shift

To understand the MOPCE terms, we consider the *E*_xr_(6S,6S) term and the related overlap integral *S*(6S,6S) which will turn out to be crucial for the shape of the intermolecular energy. As depicted in Figure 7B, *E*_xr_(6S,6S) is essentially proportional to the square of the overlap integral *S* ^2^(6S,6S). The form of the latter can be deduced most easily from the (nonsquared) overlap integral as a function of Δ*x*, which is presented in Figure 7A together with orbital representations at the extremal points. In the cofacial arrangement, the lobes of the orbitals are located exactly above each other. As the positive lobes of one pentacene molecule point to the negative ones of the other one and vice versa, we obtain a negative overlap with a relatively large absolute value of −0.08. At Δ*x* = 2.51 Å the overlap integral reaches its largest positive value. Here, the outer lobes of the two pentacene molecules do not overlap, while there are five lobes of one molecule overlapping with five of the other molecules. These lobes have the same sign resulting in positive overlap. Since the overlap is a smooth function of the *x*-shift, it has a zero between these extrema, which is found at Δ*x* = 1.40 Å. Furthermore, zeros are located at 3.80 Å, 6.32 Å, 8.90 Å, and 11.67 Å, minima at 4.98 Å and 10.05 Å as well as two additional maxima at 7.48 Å and 12.88 Å. We note, that the form of the overlap matrix element in Figure 7A is an obvious consequence of the oscillations of the 6S wave functions in *x*-direction.

**Figure 7:**
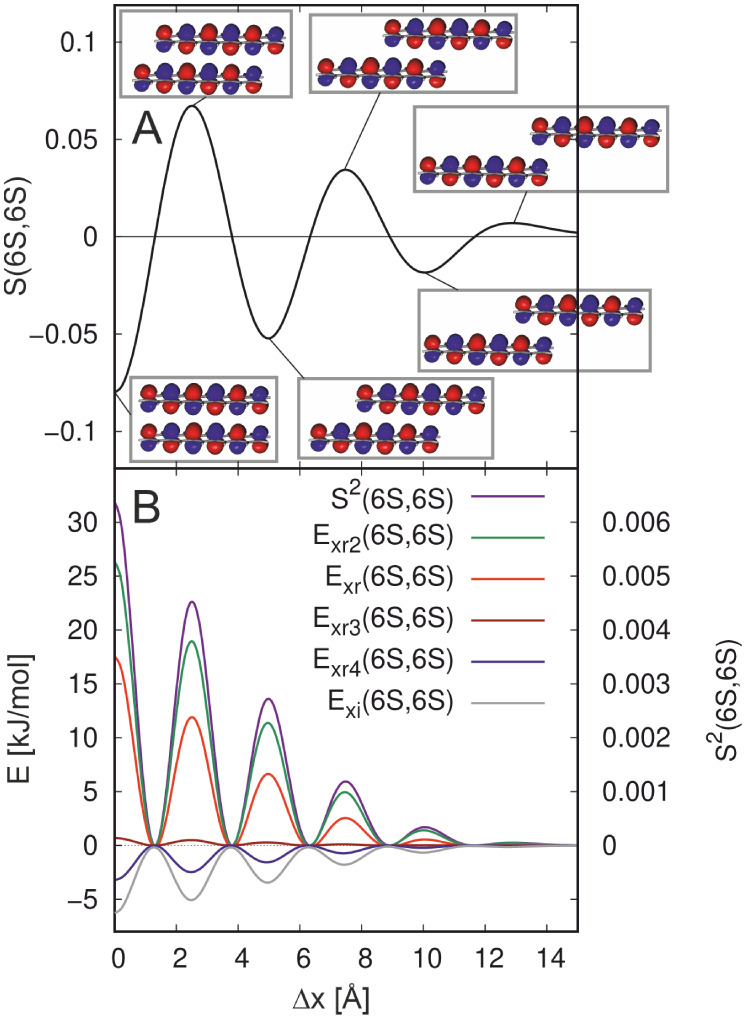
Contributions to the exchange repulsion energy and orbital overlap matrix elements for the *x*-shift of the pentacene dimer. Panel A shows the overlap of two 6S π-orbitals. The orbitals and their arrangement at the positions of the maxima and minima of *S*(6S,6S) are also indicated. In panel B, the MOPCE term *E*_xr_(6S,6S) and its contributions are shown. Their values refer to the left axis. The squared overlap of these orbitals, *S* ^2^(6S,6S) is also shown in this panel.

The MOPCE contribution *E*_xr_(6S,6S) approaches maximal values if the absolute value of *S*(6S,6S) is large, while it becomes minimal when this overlap integral is zero. Figure 7B shows that there are five minima and six maxima of *E*_xr_(6S,6S) at the above-mentioned *x*-shift values where *S*(6S,6S) has zero and extremal values, respectively. As discussed before, the orbital contributions to the exchange repulsion energy adopt slightly negative values if the absolute value of the orbital overlap approaches or reaches zero. In this case, *E*_xr2_, *E*_xr3_, and *E*_xr4_ become zero, while the negative exchange integral contribution, *E*_xi_, does not. These contributions to the exchange repulsion energy and the squared overlap matrix element of the 6S orbitals are shown in Figure 7B. While *E*_xr2_(6S,6S) is in excellent approximation proportional to *S* ^2^(6S,6S), this is still very reasonable but decreasingly well the case for *E*_xr3_(6S,6S), *E*_xr4_(6S,6S), and *E*_xi_(6S,6S). We note that *E*_xr3_(*a*,*b*) and *E*_xr4_(*a*,*b*) also behave rather similar to *S* ^2^(*a*,*b*) and *E*_xr_(*a*,*b*). As *E*_xr3_ and *E*_xr4_ contain, respectively, three and four orbital indices, orbital-pair contributions (with two orbital indices) are not uniquely defined. However, as a function of intermolecular structure, *E*_xr3_ and *E*_xr4_ behave very similar to *E*_xr2_ and *E*_xi_. The latter are unambiguously associated with two orbitals. This motivates the still empirical definition of orbital-pairs, given in eq 1 (see also ref 112).

The similarity between *S* ^2^(6S,6S) and *E*_xi_(6S,6S), apparent from Figure 7B, can be rationalized with the Mulliken approximation.^144,145^ In line with its definition as a negative exchange integral *E*_xi_(6S,6S) is always negative [see eq 5]. It reaches values between −0.2 kJ mol^–1^ and −0.05 kJ mol^–1^ at the Δ*x*-values where *S*(6S,6S) has its zero points. This leads to the somewhat nonintuitive result that orbital-pair contributions to the exchange repulsion, *E*_xr_(*a*,*b*), can take on negative values. However, if two stable ground state molecules approach each other, there are always overlapping molecular orbitals whose repulsion overweights the weak attraction of nonoverlapping MOs. Thus, *E*_xr_, the sum over all orbital-pair contributions, is, as expected, a positive quantity.

After this detailed analysis of *E*_xr_(6S,6S), we consider the other contributions of symmetric π-orbitals to the exchange repulsion energy, some of which are shown in Figure 8A and B. All contributions to *E*_xr_(*a*,*b*) are in good approximation proportional to the square of the orbital overlaps, *S* ^2^(*a*,*b*) (see Figure 8C and D). Figure 8A provides the terms *E*_xr_(*n*S,*n*S) with like orbitals, which are referred to as diagonal MOPCE terms. They are particularly large in the cofacial arrangement due to high overlaps of like π-orbitals at this position. While we observe slight differences between these contributions and the respective squared overlap matrix elements in Figure 8C, the similarities predominate. The most notable deviations between Figure 8A and C are the modifications of the *E*_xr_(1*S*,1*S*) curve between 1 and 4 Å and the relatively low values of *E*_xr_(*a*,*b*) at Δ*x* > 10 Å. Otherwise, the squared overlap reproduces the orbital-pair contributions rather accurately.

**Figure 8:**
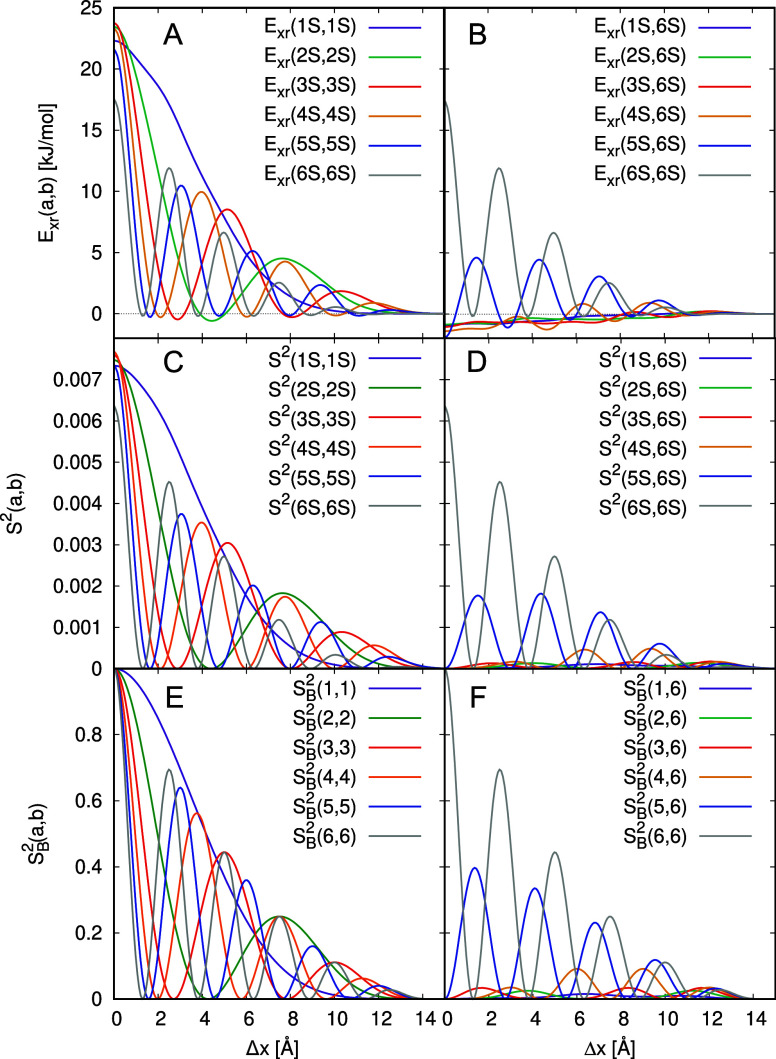
Diagonal (left) and some off-diagonal (right) contributions to *E*_xr_(*a*,*b*) (panels A and B), the squared orbital overlaps of the pentacene dimer system (panels C and D) and the squared overlaps of a particle-in-a-box model with *L* = 15 Å (panels E and F). In panels A-D only contributions of π-orbitals that are symmetric to the σ*_xz_* plane are shown and designated as *n*S for the *n*-th orbital of that symmetry.

Figure 8B and D demonstrate that the similarity between the MOPCE terms and the squared overlap matrix elements holds also for off-diagonal contributions *E*_xr_(*n*S,6S) and *S* ^2^(*n*S,6S). As shown in Figures S8–S11 in the ESI, the same is also the case for all other combinations of symmetric orbitals. Antisymmetric (A) orbitals with respect to the σ_*xz*_ plane give rise to almost identical orbital contributions for the *x*-shift as the symmetric ones with the same number of nodes in that direction. Thus, they are not presented here but collected in the ESI (Figures S12–S14).

Notably, Figure 8A and C show that the diagonal contributions *E*_xr_(*n*S,*n*S) and *S* ^2^(*n*S,*n*S) are almost independent of the orbital number *n* at the cofacial orientation (Δ*x* = 0 Å). This is not expected as the respective orbital energies differ substantially (between −0.22E_h_ and −0.56E_h_) and the long-range decay of the orbitals is determined by the orbital energies. We conclude that the overlap of the π-orbitals at the chosen inter planar distance of Δ*z* = 3.4 Å is not due to the asymptotic form of the molecular orbitals, but rather to the shape of carbon 2p-atomic orbitals in general.

As shown in Figure 8E and F, the diagonal and off-diagonal squared overlap integrals, $S_{B}^{2}\left(n,m\right)$ , of the particle-in-a-box model are rather similar to the *E*_xr_(*n*S, *m*S) MOPCE terms and even more to the *S* ^2^(*n*S, *m*S) integrals (see also Figures S9–S11 in the ESI for all $S_{B}^{2}\left(n,m\right)$ contributions). Thus, the form of the orbital-pair contributions as a function of Δ*x* can be deduced from the simple wave functions of the particle-in-a-box model. This is an important finding, as it underlines that the nodal structure of the wave functions as a function of the planar displacement in *x*-direction allows to rationalize *E*_xr_.

Panels B, D, and F in Figure 8 show that a very similar behavior is observed for *E*_xr_(*n*S, *m*S), *S* ^2^(*n*S, *m*S), and the squared overlap of the particle-in-a-box model, $S_{B}^{2}\left(n,m\right)$ for *m* = 5 and all possible *n* values. Here, the number of nodal planes also determines how often the squared overlaps become zero for the shift motion and thus also how many oscillations in *E*_xr_(*a*,*b*) can be observed. However, for the cofacial orientation, the orbital-pair contributions to the exchange repulsion energy are consistently negative if *n* ≠ *m*. Thus, the particularly large diagonal part of *E*_xr_(π–π) in the cofacial orientation is partially compensated by off-diagonal orbital-pair contributions.

In order to understand *E*_xr_(π–π) as a function of the *x*-shift, it makes sense to split it into the contributions of all symmetric π-orbitals of one monomer with the symmetric ones of the other one

19 $$E_{\mathrm{xr}}\left(\pi S-\pi S\right)=\overset{6}{\underset{n,m=1}{\sum }}E_{\mathrm{xr}}\left(nS,mS\right)$$

the antisymmetric-antisymmetric analog

20 $$E_{\mathrm{xr}}\left(\pi A-\pi A\right)=\overset{5}{\underset{n,m=1}{\sum }}E_{\mathrm{xr}}\left(nA,mA\right)$$

and a mixed contribution

21 $$E_{\mathrm{xr}}\left(\pi S-\pi A\right)=\overset{6}{\underset{n=1}{\sum }}\overset{5}{\underset{m=1}{\sum }}\left[E_{\mathrm{xr}}\left(nS,mA\right)+E_{\mathrm{xr}}\left(mA,nS\right)\right]$$

where one of the π-orbitals is symmetric and the other is antisymmetric. The resulting contributions to the exchange repulsion energy are presented in Figure 9, as well as the corresponding summed squared overlap matrix elements as e.g., $S^{2}\left(\pi \mathrm{S‐}\pi S\right)=\overset{6}{\underset{n,m=1}{\sum }}S^{2}\left(nS,mS\right)$ etc.

**Figure 9:**
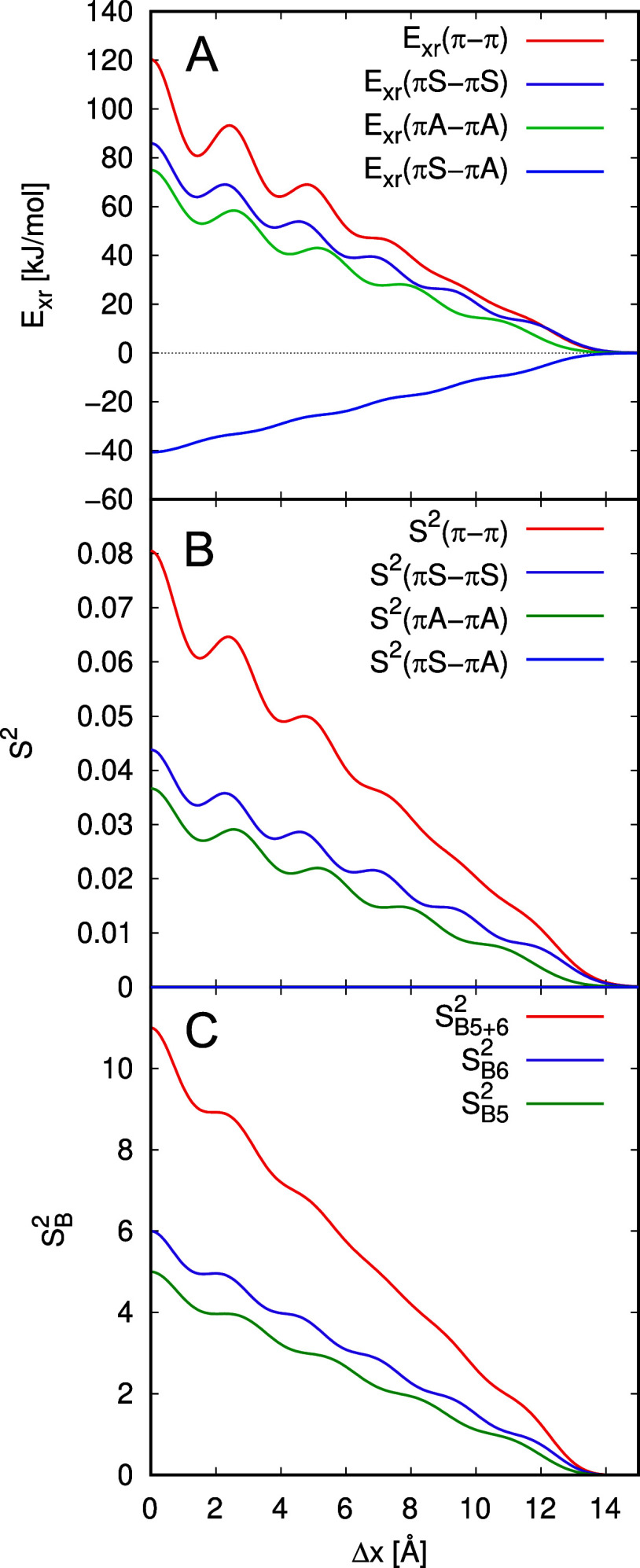
Partitioning into symmetrical and antisymmetrical π-orbital components of *E*_xr_(π–π) for the pentacene dimer (panel A), the summed squared overlap integrals *S* ^2^(π–π) (panel B), and the particle-in-a-box model for the latter integrals $S_{B5+6}^{2}$ (panel C). The contributions of symmetric (S) and antisymmetric (A) π-orbitals with respect to the σ*_xz_* mirror plane are described in the text.

For the particle-in-a-box approximation, the sum

22 $$S_{BN}^{2}=\overset{N}{\underset{n,m=1}{\sum }}S_{B}^{2}\left(n,m\right)$$

corresponds with *N* = 6 and 5 to, respectively, the SS and AA contributions of *E*_xr_(π–π) and *S* ^2^(π–π), while $S_{B5+6}^{2}=S_{B5}^{2}+S_{B6}^{2}$ is the counterpart to *E*_xr_(π–π) and *S* ^2^(π–π). The length *L* of the box was set to 15 Å which is approximately the distance between the outermost hydrogen atoms of the pentacene molecule along the *x*-coordinate. For this shift, it is expected that the overlap of the π-orbitals drops to zero. With this choice, *S* ^2^(π–π) and $S_{B5+6}^{2}$ approach zero for *x*-shift values at about 14 Å in a very similar way.

As shown in Figure 9, *E*_xr_(π–π), *S* ^2^(π–π) and $S_{B}^{2}\left(\pi -\pi \right)$ , and even more their symmetric and antisymmetric contributions are remarkably similar. The corresponding quantities *E*_xr_(πS−πS), *S* ^2^(πS−πS) and $S_{B6}^{2}$ for the symmetric [*E*_xr_(πA−πA), *S* ^2^(πA−πA) and $S_{B5}^{2}$ for the antisymmetric] orbital contributions show an oscillating decay with six [five] shoulders or minima that can be designated as “low points” in the decaying oscillation. Furthermore, there are six [five] high points. The high and low points are located at rather similar positions. However, for $S_{B6}^{2}\left[S_{B5}^{2}\right]$ , the oscillations are weak where all “low points” are actually shoulders, while at least three minima are observed for *E*_xr_(πS–σS) and *S* ^2^(πS−πS) [*E*_xr_(πA−πA) and *S* ^2^(πA−πA)]. A major difference between the π–π contributions to the exchange repulsion and the summed squared overlap is that at Δ*x* = 0 Å the mixed contribution *E*_xr_(πS−πA) is significantly negative (−15.4 kJ mol^–1^) and increases essentially linearly from there reaching zero at Δ*x* ≈ 13 Å. In contrast, all *S* ^2^(πS−πA) contributions are consistently zero as the overlap integrals vanish due to symmetry. The reason for the negative values of *E*_xr_(πS−πA) is the exchange integral contribution in eq 5. This has the important consequence that the oscillations in *E*_xr_(π–π) are more pronounced than those in *S* ^2^(π–π).

The true orbitals also show larger oscillations of the squared overlap matrix elements than the particle-in-a-box model. We associate this difference to the fact that the low points of the *S* ^2^(πS−πS) [*S* ^2^(πA−πA)] curves are located at Δ*x*-values of about 0.5, 1.5, 2.5 (i.e., half integer) multiples of the extension of a benzene ring in *x*-direction. At these positions, the overlap of a π-orbital on one pentacene with another π-orbital on the other one is reduced due to the zigzag shape of the C–C bonds along the *x*-axis. The oscillations of the squared orbital overlap curves as a function of Δ*x* are about twice as large as those of the particle-in-a-box model. Thus, the amplitude of the oscillations seem to be caused to about equal amounts by the wave functions’ amplitudes and the zigzag shape of the carbon chain along the *x*-axis.

#### Y-Shift

Figure 10 presents MOPCE contributions for the *y*-shift of the pentacene dimer as well as the corresponding squared overlap matrix elements of this system and the particle-in-a-box model. Panel A displays some of the diagonal contributions (all other contributions are collected in the ESI in Figures S16-S18). The diagonal contributions of the symmetric orbitals are very similar to each other. The absolute maximum is at Δ*y* = 0 Å with a value of 17–24 kJ mol^–1^ depending on the orbital. The curves decay to zero with increasing Δ*y* in a bell shaped manner which reaches values less than 0.1 kJ mol^–1^ at about 5 Å. Notably, the 6S orbital contributes the least repulsive diagonal contribution to *E*_xr_(π–π) in the cofacial orientation (Δ*x* = Δ*y* = 0 Å).

**Figure 10:**
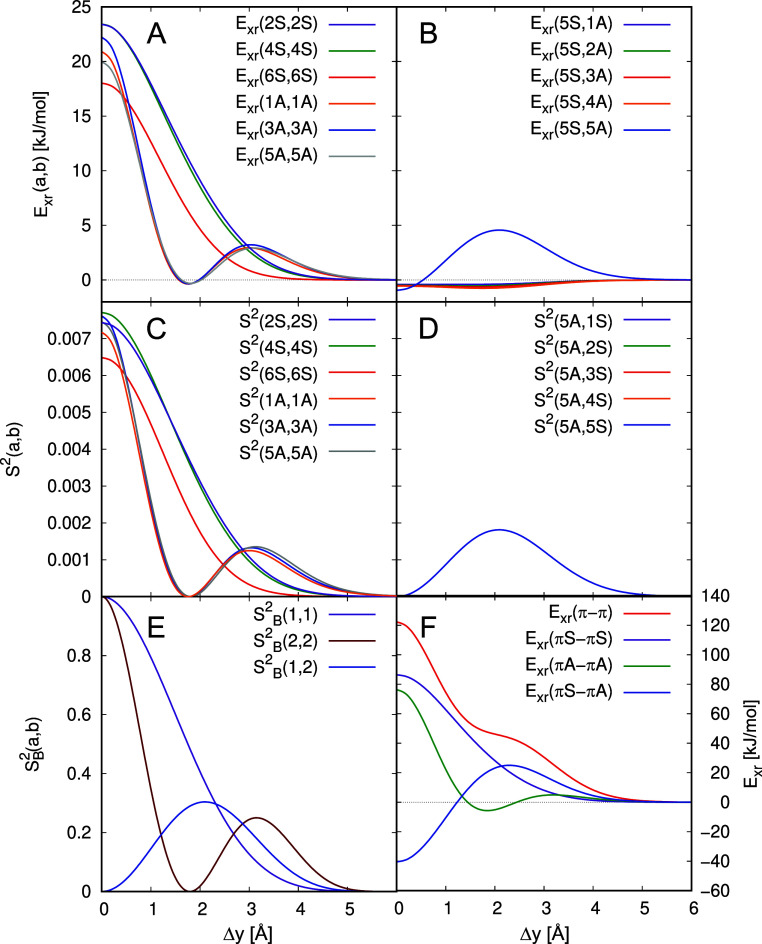
Some diagonal (panel A) and off-diagonal (panel B) contributions to *E*_xr_(*a*,*b*) as a function of Δ*y*, as well as the squared orbital overlaps of the pentacene dimer system (panels C and D) and the squared overlaps of a particle-in-a-box model with *L* = 6.3 Å (panel E). π-orbitals that are symmetric (antisymmetric) to the σ*_xz_* plane are designated as *n*S (*n*A) for the *n*-th orbital of that symmetry. Panel F shows the contributions to the exchange repulsion energy summed over either all π-orbitals or the possible symmetric and/or antisymmetric combinations of them as a function of Δ*y*.

The magnitudes of the diagonal contributions of the antisymmetric π-orbitals presented in Figure 10A are again hardly dependent on the orbital energies. The maximum of the *E*_xr_(*n*A,*n*A) curves is also found at Δ*y* = 0 Å and amounts to 20–22 kJ mol^–1^. All contributions steeply drop to a minimum with slightly negative values of about −0.35 kJ mol^–1^ at about Δ*y* = 1.8 Å, increase again to a second maximum at Δ*y* ≈ 3 Å with *E*_xr_(*n*A,*n*A) ≈ 3 kJ mol^–1^ and drop off with increasing Δ*y*, reaching values below 0.1 kJ mol^–1^ at about Δ*y* = 5.5 Å. The off-diagonal MOPCE contributions are consistently small, except for the *E*_xr_(*n*S,*n*A) terms. As shown in Figure 10B, the respective *n* = 5 element has its absolute minimum at Δ*y* = 0 Å with −0.4 kJ mol^–1^ and increases to 5.7 kJ mol^–1^ at Δ*y* = 2.1 Å. From there it decays as the diagonal contributions *E*_xr_(*n*A,*n*A). As before, the form of these curves is very well reproduced by the squared orbital overlap matrix elements, which are shown in Figure 10C and D. The most notable difference is again that the MOPCE contributions have slightly negative values at their minima, while *S* ^2^(*a*,*b*) shows minima at very similar positions but with a value of zero. As shown in Figure 10E, the shape of the MOPCE contributions is also nicely reproduced by the particle-in-a-box model. According to this model, $S_{B}^{2}\left(1,1\right)$ and $S_{B}^{2}\left(2,2\right)$ approximate the contributions of two like orbitals having, respectively, no and one nodal plane in *y*-direction. Their shape agrees very well with the *E*_xr_(*n*S,*n*S) and *E*_xr_(*n*A,*n*A) contributions, respectively, in Figure 10A and $S_{B}^{2}\left(1,2\right)$ is very similar to *E*_xr_(5S,5A) and *S* ^2^(5S,5A) in Figure 10B.

Summing the MOPCE contributions over all π-orbitals or over all pairs of symmetric and antisymmetric orbitals gives rise to the contributions to *E*_xr_ shown in Figure 10F. *E*_xr_(π–π) is again decisive for the position of the minimum of the interaction energy along the *y*-shift due to its shoulder at about Δ*y* = 1.5 Å, which is caused by the MOPCE contributions of like antisymmetric orbitals *E*_xr_(*n*A,*n*A) shown in Figure 10A. The latter can be rationalized with the squared orbital overlap and even with the much simpler particle-in-a-box model. The oscillations of *E*_xr_ map the nodal structure of the underlying electronic wave functions. By adding the much smoother attractive interactions shown in Figure 4, these oscillations turn into the pronounced minima of the *E*_int_ curve.

#### X-Shift for Δ*y* = 1 Å

So far we have focused our considerations on the analysis of the *x*- and *y*-shift motions starting from the cofacial arrangement. However, this does not lead to the global minimum of the Pen_2_-system which is found at Δ*x* = 1.27 Å and Δ*y* = 1.01 Å. As the minimum structure is of particular relevance, we consider MOPCE terms for Δ*y* = 1 Å which are shown as a function of Δ*x* in Figure 11. The shape of the MOPCE terms in panels A-D of the figure are rather similar to those in Figure 8 where Δ*y* = 0 Å. The maximum values of the *E*_xr_(*n*S,*n*S) diagonal orbital-pair contributions at the cofacial orientation amount to about 25 kJ/mol and decrease to about 17 kJ/mol at Δ*x* = 0 Å, Δ*y* = 1 Å. A much more significant decrease by a factor of about 3–4 can be observed for the *E*_xr_(*n*A,*n*A) terms in Figure 11C and D (see the respective curves in Figures S8, S12, S13 and S14 in the ESI). This is obviously associated with the node of the antisymmetric orbitals in the σ_*xz*_ plane and leads to the interesting observation that the exchange repulsion interaction of all antisymmetric orbitals decreases substantially. In Figure 11E we observe six shoulders for the exchange repulsion contributions of *E*_xr_(π–π) which can be traced back to the *E*_xr_(*n*S,*m*S) contributions. In contrast, the local maximum at about 12 Å is not visible for the *E*_xr_(π–π) with Δ*y* = 0 Å in Figure 9A due to the phase shift of the *E*_xr_(πS−πS) and the *E*_xr_(πA−πA) contributions.

**Figure 11:**
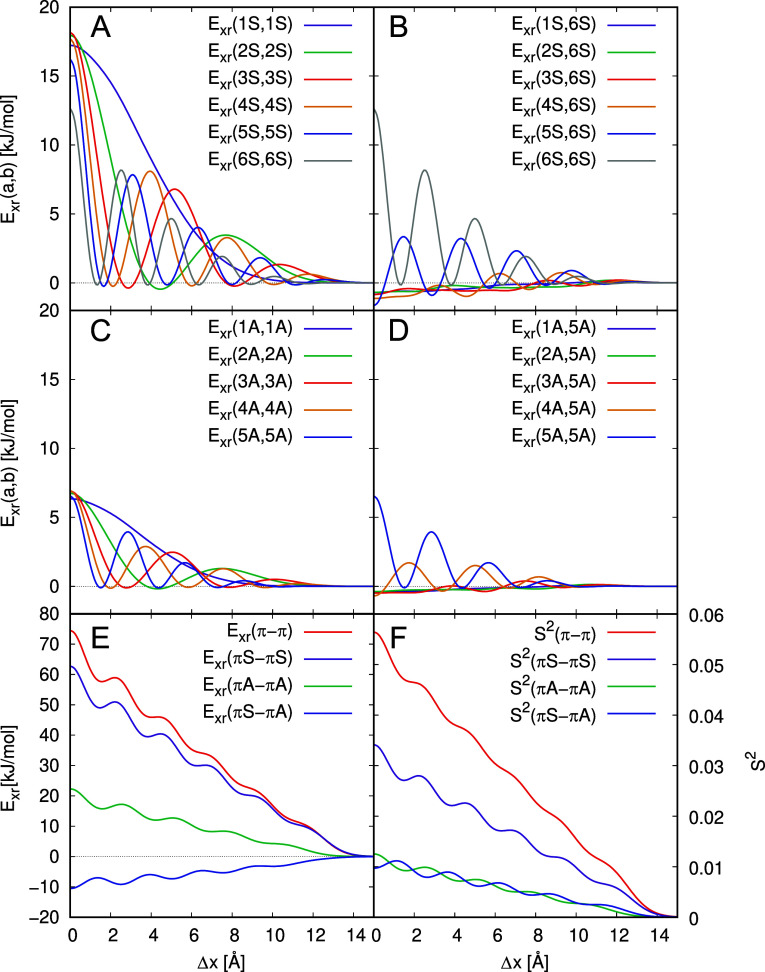
MOPCE terms of a pentacene dimer with Δ*y* = 1 Å as a function of Δ*x*. For the symmetric orbitals with respect to the σ*_xz_* plane the diagonal contributions (panel A) and those of the S6 orbital (panel B) are shown. For the antisymmetric orbitals the diagonal contributions (panel C) and those of orbital 5A (panel D) are presented. Panel E shows the *E*_xr_(π–π) as well as a partitioning into symmetric and antisymmetric parts of it. The respective summed squared orbital overlap matrix elements are given in panel F.

Again the oscillations follow features that are due to the MOPCE contributions of the orbitals with the highest number of nodes in the direction of the motion. In this case, however, different orbitals are responsible for the position of the minimum structure. While the MOPCE terms of all antisymmetric orbitals are small for Δ*y* in the range between about 1–2 Å, the most important diagonal MOPCE term *E*_xr_(6S,6S) becomes minimal for Δ*x* ≈ 1.5 Å. This is in line with the position of the minimum at Δ*x* = 1.27 Å, Δ*y* = 1.01 Å if we consider that attractive contributions to the interaction energy prefer the cofacial structure.

### Trends for Other Acenes

The results obtained for the pentacene dimer make it possible to rationalize the intermolecular potentials of the other acene dimers in the considered slip-stacked arrangements on the basis of the shape of the well-known π-type molecular orbitals of the monomers.^14,146,147^ For all acene dimers, the MOPCE contributions to the exchange repulsion energy between antisymmetric orbitals are particularly low if Δ*y* ≈ 1 Å. Furthermore, the combined MOPCE contributions of the symmetric orbitals cause a relatively low exchange repulsion interaction if the Δ*x*-value is about 0.5, 1.5, 2.5 or 3.5 times the extension of an aromatic ring in *x*-direction. In combination with much smoother attractive dispersion and electrostatic interactions these particularly low repulsive interactions explain the positions of the minima of *E*_int_ shown in Figure 4. We note that the location of these minima is fully in line with e.g., the graphite-like AB (Bernal-type)^148^ packing, with the demonstration of the importance of exchange repulsion for π-stacked systems by Herbert and Carter-Fenk,^87,88^ and with recent work of Bayse on pentacene dimers.^106^ The latter work explains π-stacked arrangements on the basis of bond orders between like π-orbitals and uses a more empirical, but otherwise similar concept as the present work. Similar considerations have been made in the works of Bayse and coworkers^98,104,105^ and of Zhao and Zhang.^107^ We believe that the model presented here should be a further step toward a proper understanding of π-stacked interactions.

## Summary and Conclusions

As recently shown by the present authors,^86,112^ the exchange repulsion energy can be partitioned into pair contributions, *E*_xr_(*a*,*b*), where *a* and *b* represent occupied molecular orbitals of the two interacting systems A and B. For the here considered π-stacked arrangements, these Molecular Orbital-Pair Contributions to the Exchange repulsion (MOPCE) terms are small if *a* and *b* are σ-orbitals. Contributions with one σ- and one π-orbital are roughly of similar magnitude as the much less numerous π–π contributions. The latter play a key role for the structure of the exchange repulsion energy. For motions of one acene molecule in its molecular plane and constant inter planar distance, the MOPCE terms turn out to be essentially proportional to the squared overlap, $S_{ab}^{2}$ , of the orbitals. As the orbital overlap is readily rationalized, this provides a vivid explanation for the anisotropy of the exchange repulsion. Furthermore, it can be reasonably approximated with a much simpler model of particle-in-a-box wave functions. These quantities map the nodal structure of the wave functions along the considered motions. The oscillatory structure of *E*_xr_ as a function of the longitudinal shift (Δ*x*) is amplified by attractive exchange integral contributions as well as the actual arrangements of facing atoms.

Our considerations explain the particularly unfavorable exchange repulsion energy for cofacial arrangements of like π-systems. Here, all lobes of the π-orbitals on one system are facing the respective lobes on the other system, resulting in maximum squared overlap. The oscillations in *E*_xr_ resemble the squared overlap of the π-orbitals with the highest number of nodes perpendicular to the shift direction. However, in the case of the acene dimers, nodes in longitudinal and transversal direction are both relevant. This explains the slip-stacked arrangement of the acene dimers and similar systems, which are consistent with available crystal structures. Our results are in line with qualitative investigations by Bayse et al.^98,104–106^ as well as Zhao and Zhang^107^ who proposed that orbital interactions play a major role for π–π interactions. We confirm this assumption and complement it with a rigorous partitioning of the well-established exchange repulsion interaction.

Speaking in models, we have shown that π-systems see each other not as flat as Lewis structures or the picture of diffuse π-electron clouds might suggest, nor is their shape determined by the atoms in the sense of a ball-and-stick model, which scrape against each other in the course of a slip-stacked motion. It is the nodal structure of the wave functions that causes oscillations in the intermolecular potential energy surface via the exchange repulsion. That means that the quantum-mechanical nature of the molecules must be taken into account to explain the preferred arrangements of π-stacked aggregates, which cannot be captured by purely classical models. Although exemplified on the case of acenes, our approach can be generalized for further systems, e.g., donor acceptor complexes or even halogen bonds.

The MOPCE approach rationalizes the importance and the properties of the exchange repulsion energy, which is otherwise an elusive quantity. However, several findings of the present work require further investigation. Among them is the importance of induction energy, which, albeit being relatively small, tends to enhance the shape of the potential energy surface systematically. Furthermore, similar investigations of the components of *E*_xr_ and a deeper analysis of their physical nature will be helpful to complete our picture about molecular interactions. For that purpose, other arrangements, including rotations of the monomers and changes of the inter planar distance as well as considerations of different systems should also be investigated. Finally, we believe that the present results will allow improving the so far limited reliability of force fields for slip-stacked π-systems.^149–151^ This seems possible, as even molecular orbitals of rather simple quality seem to be sufficient for a qualitatively correct representation of the contributions to the exchange repulsion energy.

## Supplementary Material





## Data Availability

The data underlying this study are available in the published article and its Supporting Information.

## Notes

Numerical values of the energy contributions and *xyz* coordinates of the acene monomers (ZIP)
Figures S1–S18; Tables S1–S8; and *xyz* coordinates of the acene monomers (PDF)

## References

[1] Burley S. K., Petsko G. A. (1985). Aromatic-Aromatic Interaction: A Mechanism of Protein
Structure Stabilization. Science.

[2] Meyer E. A., Castellano R. K., Diederich F. (2003). Interactions with Aromatic Rings
in Chemical and Biological Recognition. Angew.
Chem., Int. Ed..

[3] Martinez C. R., Iverson B. L. (2012). Rethinking the term “pi-stacking”. Chem. Sci..

[4] Ninković D. B., Filipović J. P. B., Hall M. B., Brothers E. N., Zarić S. D. (2020). What Is Special about Aromatic–Aromatic Interactions?
Significant Attraction at Large Horizontal Displacement. ACS Cent. Sci..

[5] Hobza P. (2008). Stacking interactions. Phys. Chem. Chem. Phys..

[6] Wang C., Dong H., Jiang L., Hu W. (2018). Organic semiconductor
crystals. Chem. Soc. Rev..

[7] Zhang J., Xu W., Sheng P., Zhao G., Zhu D. (2017). Organic Donor–Acceptor
Complexes as Novel Organic Semiconductors. Acc.
Chem. Res..

[8] Chakrabarti P., Bhattacharyya R. (2007). Geometry of nonbonded interactions involving planar
groups in proteins. Prog. Biophys. Mol. Biol..

[9] Živković J. M., Stanković I. M., Ninković D. B., Zarić S. D. (2021). Decisive
Influence of Environment on Aromatic/Aromatic Interaction Geometries
Comparison of Aromatic/Aromatic Interactions in Crystal Structures
of Small Molecules and in Protein Structures. Cryst. Growth Des..

[10] Carter-Fenk K., Liu M., Pujal L., Loipersberger M., Tsanai M., Vernon R. M., Forman-Kay J. D., Head-Gordon M., Heidar-Zadeh F., Head-Gordon T. (2023). The Energetic Origins of Pi–Pi Contacts in Proteins. J. Am. Chem. Soc..

[11] Anthony J. E. (2006). Functionalized
Acenes and Heteroacenes for Organic Electronics. Chem. Rev..

[12] Anthony J.
E. (2008). The Larger
Acenes: Versatile Organic Semiconductors. Angew.
Chem., Int. Ed..

[13] Bettinger H. F. (2010). Electronic
structure of higher acenes and polyacene: The perspective developed
by theoretical analyses. Pure Appl. Chem..

[14] Korytár R., Xenioti D., Schmitteckert P., Alouani M., Evers F. (2014). Signature
of the Dirac cone in the properties of linear oligoacenes. Nat. Commun..

[15] Bettinger H. F., Tönshoff C. (2015). The Longest
Acenes. Chem. Rec..

[16] Cardia R., Malloci G., Bosin A., Serra G., Cappellini G. (2016). Computational
investigation of the effects of perfluorination on the charge-transport
properties of polyaromatic hydrocarbons. Chem.
Phys..

[17] Yang Y., Davidson E. R., Yang W. (2016). Nature of ground and electronic excited
states of higher acenes. Proc. Natl. Acad. Sci.
U. S. A..

[18] Einholz R., Fang T., Berger R., Grüninger P., Früh A., Chassé T., Fink R. F., Bettinger H. F. (2017). Heptacene:
Characterization in Solution, in the Solid State, and in Films. J. Am. Chem. Soc..

[19] Tykwinski R. R. (2019). Synthesis
of Unsymmetrical Derivatives of Pentacene for Materials Applications. Acc. Chem. Res..

[20] Geiger T. (2020). Modulating the Electronic and Solid-State Structure
of Organic Semiconductors
by Site-Specific Substitution: The Case of Tetrafluoropentacenes. Chem. Eur. J..

[21] Hayashi H., Yamada H. (2025). Exploring the chemistry of higher acenes: from synthesis
to applications. Chem. Sci..

[22] Fishman V., Lesiuk M., Martin J. M. L., Daniel Boese A. (2025). Another Angle
on Benchmarking Noncovalent Interactions. J.
Chem. Theory Comput..

[23] Podeszwa R., Bukowski R., Szalewicz K. (2006). Potential
Energy Surface for the
Benzene Dimer and Perturbational Analysis of *π*—*π* Interactions. J. Phys. Chem. A.

[24] Sinnokrot M., Sherrill C. (2006). High-Accuracy Quantum Mechanical Studies of *π*-*π* Interactions in Benzene
Dimers. J. Phys. Chem. A.

[25] Schnell M., Erlekam U., Bunker P. R., von Helden G., Grabow J., Meijer G., van der
Avoird A. (2013). Structure
of the Benzene Dimer - Governed by Dynamics. Angew. Chem., Int. Ed..

[26] Karton A., Martin J. M. L. (2021). Prototypical π–π dimers re-examined
by means of high-level CCSDT­(Q) composite ab initio methods. J. Chem. Phys..

[27] Czernek J., Brus J. (2024). Revisiting the Most
Stable Structures of the Benzene Dimer. Int.
J. Mol. Sci..

[28] Lee E. C., Kim D., Jurecka P., Tarakeshwar P., Hobza P., Kim K. S. (2007). Understanding
of Assembly Phenomena by Aromatic–Aromatic Interactions: Benzene
Dimer and the Substituted Systems. J. Phys.
Chem. A.

[29] Sinnokrot M. O., Valeev E. F., Sherrill C. D. (2002). Estimates of the Ab Initio Limit
for *π*–*π* Interactions:
The Benzene Dimer. J. Am. Chem. Soc..

[30] Sinnokrot M. O., Sherrill C. D. (2004). Highly Accurate
Coupled Cluster Potential Energy Curves
for the Benzene Dimer: Sandwich, T-Shaped, and Parallel-Displaced
Configurations. J. Phys. Chem. A.

[31] Schwoerer, M. ; Wolf, H. C. Organic Molecular Solids; Wiley-VCH: Berlin, 2006.

[32] Curtis M.
D., Cao J., Kampf J. W. (2004). Solid-State Packing of Conjugated Oligomers: From *π*-Stacks to the Herringbone Structure. J. Am. Chem. Soc..

[33] Moon H., Zeis R., Borkent E.-J., Besnard C., Lovinger A. J., Siegrist T., Kloc C., Bao Z. (2004). Synthesis, Crystal
Structure, and Transistor Performance of Tetracene Derivatives. J. Am. Chem. Soc..

[34] Chi X., Li D., Zhang H., Chen Y., Garcia V., Garcia C., Siegrist T. (2008). 5,6,11,12-Tetrachlorotetracene, a tetracene derivative
with *π*–stacking structure: The synthesis,
crystal structure and transistor properties. Org. Electron..

[35] Kanazawa K., Bulgarevich K., Kawabata K., Takimiya K. (2023). Methylthiolation
of
Acenes: Change of Crystal Structure from Herringbone to Rubrene-like
Pitched *π*-Stacking Structure. Crys. Growth Des..

[36] Jurchescu O. D., Meetsma A., Palstra T. T. M. (2006). Low-temperature structure of rubrene
single crystals grown by vapor transport. Acta
Cryst. B.

[37] Huang L., Liao Q., Shi Q., Fu H., Ma J., Yao J. (2010). Rubrene micro-crystals from solution routes: their crystallography,
morphology and optical properties. J. Mater.
Chem..

[38] McGarry K.
A., Xie W., Sutton C., Risko C., Wu Y., Young V. G., Brédas J.-L., Frisbie C. D., Douglas C. J. (2013). Rubrene-Based Single-Crystal
Organic Semiconductors: Synthesis, Electronic Structure, and Charge-Transport
Properties. Chem. Mater..

[39] Mamada M., Katagiri H., Sakanoue T., Tokito S. (2015). Characterization of
New Rubrene Analogues with Heteroaryl Substituents. Crys. Growth Des..

[40] Moret M., Gavezzotti A. (2022). The crystalline state of rubrene materials: intermolecular
recognition, isomorphism, polymorphism, and periodic bond-chain analysis
of morphologies. New J. Chem..

[41] Chen Z., Müller P., Swager T. M. (2006). Syntheses of Soluble, *π*-Stacking
Tetracene Derivatives. Org. Lett..

[42] Kobayashi K., Masu H., Shuto A., Yamaguchi K. (2005). Control of
Face-to-Face *π*-*π* Stacked
Packing Arrangement of Anthracene Rings via Chalcogen-Chalcogen Interaction:
9,10-Bis­(methylchalcogeno)­anthracenes. Chem.
Mater..

[43] Kobayashi K., Shimaoka R., Kawahata M., Yamanaka M., Yamaguchi K. (2006). Synthesis
and Cofacial *π*-Stacked Packing Arrangement
of 6,13-Bis­(alkylthio)­pentacene. Org. Lett..

[44] Miao Q., Lefenfeld M., Nguyen T., Siegrist T., Kloc C., Nuckolls C. (2005). Self–Assembly
and Electronics of Dipolar Linear
Acenes. Adv. Mater..

[45] Anthony J. E., Brooks J. S., Eaton D. L., Parkin S. R. (2001). Functionalized Pentacene:
Improved Electronic Properties from Control of Solid-State Order. J. Am. Chem. Soc..

[46] Anthony J. E., Eaton D. L., Parkin S. R. (2002). A Road Map to Stable, Soluble, Easily
Crystallized Pentacene Derivatives. Org. Lett..

[47] Li Y., Wu Y., Liu P., Prostran Z., Gardner S., Ong B. S. (2007). Stable
Solution-Processed High-Mobility Substituted Pentacene Semiconductors. Chem. Mater..

[48] Swartz C. R., Parkin S. R., Bullock J. E., Anthony J. E., Mayer A. C., Malliaras G. G. (2005). Synthesis and Characterization of Electron-Deficient
Pentacenes. Org. Lett..

[49] Shu Y., Lim Y.-F., Li Z., Purushothaman B., Hallani R., Kim J. E., Parkin S. R., Malliaras G. G., Anthony J. E. (2011). A survey of electron-deficient pentacenes
as acceptors
in polymer bulk heterojunction solar cells. Chem. Sci..

[50] Combe C. M., James D. T., Wade J., White A. J., Kim J.-S., McCulloch I. (2013). Synthesis
and morphology of asymmetric, alkyne-functionalised
pentacene and 2-fluoroanthradithiophene. Tetrahedron
Lett..

[51] Miao Q., Chi X., Xiao S., Zeis R., Lefenfeld M., Siegrist T., Steigerwald M. L., Nuckolls C. (2006). Organization of Acenes
with a Cruciform Assembly Motif. J. Am. Chem.
Soc..

[52] Briseno A.
L., Miao Q., Ling M.-M., Reese C., Meng H., Bao Z., Wudl F. (2006). Hexathiapentacene: Structure, Molecular Packing, and
Thin-Film Transistors. J. Am. Chem. Soc..

[53] Shi X., Kueh W., Zheng B., Huang K., Chi C. (2015). Dipolar Quinoidal
Acene Analogues as Stable Isoelectronic Structures of Pentacene and
Nonacene. Angew. Chem., Int. Ed..

[54] Lijina M. P., Benny A., Ramakrishnan R., Nair N. G., Hariharan M. (2020). Exciton Isolation
in Cross-Pentacene Architecture. J. Am. Chem.
Soc..

[55] Sutton C., Risko C., Brédas J.-L. (2016). Noncovalent Intermolecular Interactions
in Organic Electronic Materials: Implications for the Molecular Packing
vs Electronic Properties of Acenes. Chem. Mater..

[56] Chen Y., Wang Y.-B., Zhang Y., Wang W. (2017). Accurate calculations
of the noncovalent systems with flat potential energy surfaces: Naphthalene
dimer and azulene dimer. Comput. Theor. Chem..

[57] Cabaleiro-Lago E. M., Rodríguez-Otero J. (2018). On the Nature
of *σ*-*σ*, *σ*-*π*, and *π*-*π* Stacking
in Extended Systems. ACS Omega.

[58] Lemmens A. K., Chopra P., Garg D., Steber A. L., Schnell M., Buma W. J., Rijs A. M. (2021). High-resolution
infrared spectroscopy
of naphthalene and acenaphthene dimers. Mol.
Phys..

[59] Hoche J., Flock M., Miao X., Philipp L. N., Wenzel M., Fischer I., Mitric R. (2021). Excimer formation
dynamics in the
isolated tetracene dimer. Chem. Sci..

[60] Valente C. A. D., do Casal M. T., Barbatti M., Niehaus T. A., Aquino A. J. A., Lischka H., Cardozo T. M. (2021). Excitonic
and charge transfer interactions
in tetracene stacked and T-shaped dimers. J.
Chem. Phys..

[61] Alessa A. H. (2025). Analyzing
the Energetics of the Four Aromatic Ring Interactions: Theoretical
Study. J. Phys. Chem. A.

[62] Bhattacharjee R., Lischka H., Kertesz M. (2025). Pancake Bonding
in the Stabilization
of Cationic Acene Dimers. ACS Mater. Au.

[63] Matsokin N. A., Kalinin M., Buchwald A., Werner H.-J., Fink R. F., Fink K., Sharapa D. I. (2026). Revisiting
Acene Dimers: A Comprehensive
Theoretical Study of a Less Explored Conformer. J. Chem. Phys.

[64] Sandwich arrangements of π-systems are, however, known for unsaturated (radical) systems,^31,62,65^ binary crystals with alternant molecules,^31,68–73^ or borazine.^66,67^ In these cases the interactions are either of covalent character or there are no more identical monomers with cofacially arranged molecules or the molecules are rotated such that nitrogen atoms come to lie above borons and vice versa.

[65] Kertesz M. (2019). Pancake Bonding:
An Unusual Pi–Stacking Interaction. Chem.
Eur. J..

[66] Kawahara S.-I., Tsuzuki S., Uchimaru T. (2003). Ab initio calculation of interaction
nature of borazine (B_3_N_3_H_6_) dimer. J. Chem. Phys..

[67] Bettinger H. F., Kar T., Sánchez-García E. (2009). Borazine and Benzene Homo- and Heterodimers. J. Phys. Chem. A.

[68] Wright, J. D. Molecular crystals, 2nd ed.; Cambridge University Press, 1995.

[69] Williams R. M., Wallwork S. C. (1966). Molecular complexes exhibiting polarization bonding.
VI. The crystal structure of the 2,4,6-tri­(dimethylamino)-1,3,5-triazine–s-trinitrobenzene
complex. Acta Crystallogr..

[70] Williams R. M., Wallwork S. C. (1968). Molecular complexes
exhibiting polarization bonding.
XI. The crystal and molecular structure of the 7,7,8,8-tetracyanoquinodimethane–anthracene
complex. Acta Crys. Sect. B.

[71] Aquino A. A. J., Borges I., Nieman R., Köhn A., Lischka H. (2014). Intermolecular interactions and charge
transfer transitions
in aromatic hydrocarbon–tetracyanoethylene complexes. Phys. Chem. Chem. Phys..

[72] Rather S. A., Saraswatula V. G., Sharada D., Saha B. K. (2019). Influence of molecular
width on the thermal expansion in solids. New
J. Chem..

[73] Mayoh B., Prout C. K. (1972). Molecular complexes.
Part 13.–Influence of charge
transfer interactions on the structures of *π*-*π** electron donor-acceptor molecular complexes. J. Chem. Soc., Faraday Trans. 2.

[74] Hunter C. A., Sanders J. K. M. (1990). The nature of *π*–*π* interactions. J. Am. Chem.
Soc..

[75] Wheeler S. E. (2025). Revisiting
the Hunter-Sanders Model for *π*–*π* Interactions. J. Am. Chem.
Soc..

[76] Schramm B., Gray M., Herbert J. M. (2025). Substituent and Heteroatom Effects
on *π*–*π* Interactions:
Evidence That Parallel-Displaced *π*-Stacking
is Not Driven by Quadrupolar Electrostatics. J. Am. Chem. Soc..

[77] Hunter C. A., Lawson K. R., Perkins J., Urch C. J. (2001). Aromatic interactions. J. Chem. Soc., Perkin Trans. 2.

[78] Williams D. (1999). Improved intermolecular
force field for crystalline hydrocarbons containing four- or three-coordinated
carbon. J. Mol. Struct..

[79] Fagnani, D. E. ; Sotuyo, A. ; Castellano, R. K. In Comprehensive supramolecular chemistry II, Atwood, J. L. ed.; Elsevier: Oxford, 2017 pp. 121–148.

[80] Fleming, I. Molecular Orbitals and Organic Chemical Reactions, The international series of monographs on chemistry; John Wiley and Sons: Chichester, 2010.

[81] Sinnokrot M. O., Sherrill C. D. (2004). Substituent Effects
in *π*–*π* Interactions:
Sandwich and T-Shaped Configurations. J. Am.
Chem. Soc..

[82] Hohenstein E. G., Sherrill C. D. (2009). Effects
of Heteroatoms on Aromatic *π*–*π* Interactions: Benzene– Pyridine
and Pyridine Dimer. J. Phys. Chem. A.

[83] Hohenstein E. G., Duan J., Sherrill C. D. (2011). Origin
of the Surprising Enhancement
of Electrostatic Energies by Electron-Donating Substituents in Substituted
Sandwich Benzene Dimers. J. Am. Chem. Soc..

[84] Sherrill C. D. (2013). Energy
Component Analysis of *π* Interactions. Acc. Chem. Res..

[85] Parrish R. M., Sherrill C. D. (2014). Quantum-Mechanical Evaluation of *π*–*π* versus Substituent– *π* Interactions in *π* Stacking:
Direct Evidence for the Wheeler–Houk Picture. J. Am. Chem. Soc..

[86] Henrichsmeyer J., Thelen M., Bröckel M., Fadel M., Behnle S., Sekkal-Rahal M., Fink R. F. (2023). Rationalizing Aggregate Structures
with Orbital Contributions to the Exchange-Repulsion Energy. ChemPhysChem.

[87] Carter-Fenk K., Herbert J. M. (2020). Electrostatics does not dictate the slip-stacked arrangement
of aromatic *π*–*π* interactions. Chem. Sci..

[88] Carter-Fenk K., Herbert J. M. (2020). Reinterpreting *π*-stacking. Phys. Chem. Chem.
Phys..

[89] Carter-Fenk K., Lao K. U., Herbert J. M. (2021). Predicting and Understanding Non-Covalent
Interactions Using Novel Forms of Symmetry-Adapted Perturbation Theory. Acc. Chem. Res..

[90] Herbert J. M. (2021). Neat, Simple,
and Wrong: Debunking Electrostatic Fallacies Regarding Noncovalent
Interactions. J. Phys. Chem. A.

[91] Ryno S. M., Risko C., Brédas J.-L. (2016). Noncovalent
Interactions and Impact
of Charge Penetration Effects in Linear Oligoacene Dimers and Single
Crystals. Chem. Mater..

[92] Jeziorski B., Moszynski R., Szalewicz K. (1994). Perturbation Theory Approach to Intermolecular
Potential Energy Surfaces of van der Waals Complexes. Chem. Rev..

[93] Parker T. M., Burns L. A., Parrish R. M., Ryno A. G., Sherrill C. D. (2014). Levels
of symmetry adapted perturbation theory (SAPT). I. Efficiency and
performance for interaction energies. J. Chem.
Phys..

[94] Stone, A. J. The Theory of Intermolecular Forces, The international series of monographs on chemistry; Clarendon Press: Oxford, 2002; Vol. 31.

[95] Wang B., Truhlar D. G. (2010). Including Charge
Penetration Effects in Molecular Modeling. J.
Chem. Theory Comput..

[96] Tafipolsky M., Engels B. (2011). Accurate Intermolecular Potentials with Physically
Grounded Electrostatics. J. Chem Theor. Comput..

[97] Cabaleiro-Lago E. M., Rodríguez-Otero J., Vázquez S. A. (2022). Electrostatic
penetration effects stand at the heart of aromatic *π* interactions. Phys. Chem. Chem. Phys..

[98] Lutz P. B., Bayse C. A. (2013). Orbital-based insights
into parallel-displaced and
twisted conformations in *π*—*π* interactions. Phys. Chem. Chem. Phys..

[99] Murrell J., Teixeira-Dias J. (1970). The dependence
of exchange energy on orbital overlap. Mol.
Phys..

[100] Gresh N., Claverie P., Pullman A. (1986). Intermolecular interactions:
Elaboration on an additive procedure including an explicit charge-transfer
contribution. Int. J. Quantum Chem..

[101] Gresh N., Piquemal J.-P., Krauss M. (2005). Representation
of Zn­(II)
complexes in polarizable molecular mechanics. Further refinements
of the electrostatic and short-range contributions. Comparisons with
parallel ab initio computations. J. Comput.
Chem..

[102] Söderhjelm P., Karlström G., Ryde U. (2006). Comparison of overlap-based
models for approximating the exchange-repulsion energy. J. Chem. Phys..

[103] Rackers J. A., Ponder J. W. (2019). Classical Pauli
repulsion: An anisotropic,
atomic multipole model. J. Chem. Phys..

[104] Lutz P. B., Bayse C. A. (2018). Interpreting geometric preferences
in *π*-stacking interactions through molecular
orbital analysis. Int. J. Quantum Chem..

[105] Bayse C. A. (2023). Stack bonding in polyaromatic hydrocarbons. Phys. Chem. Chem. Phys..

[106] Bayse C. A. (2025). Stack bonding
in pentacene and its derivatives. Phys. Chem.
Chem. Phys..

[107] Zhao R., Zhang R.-Q. (2016). A new insight into *π*–*π* stacking involving remarkable orbital
interactions. Phys. Chem. Chem. Phys..

[108] Kim Y. S., Kim S. K., Lee W. D. (1981). Dependence
of the
closed-shell repulsive interaction on the overlap of the electron
densities. Chem. Phys. Lett..

[109] Wheatley R. J., Price S. L. (1990). A systematic intermolecular
potential
method applied to chlorine. Mol. Phys..

[110] Patkowski K. (2020). Recent developments in symmetry-adapted
perturbation
theory. WIREs Comput. Mol. Sci..

[111] Szalewicz K., Jeziorski B. (2022). Physical mechanisms of intermolecular
interactions from symmetry-adapted perturbation theory. J. Mol. Model..

[112] Henrichsmeyer J., Thelen M., Fink R. F. (2025). What is
the Exchange
Repulsion Energy? Insight by Partitioning into Physically Meaningful
Contributions. ChemPhysChem.

[113] Balasubramani S. G., Chen G. P., Coriani S., Diedenhofen M., Frank M. S., Franzke Y. J., Furche F., Grotjahn R., Harding M. E., Hättig C. (2020). TURBOMOLE: Modular program
suite for ab initio quantum-chemical and condensed-matter simulations. J. Chem. Phys..

[114] Klebe G., Graser F., Hädicke E., Berndt J. (1989). Crystallochromy as a solid-state effect: correlation
of molecular conformation, crystal packing and colour in perylene-3,4:
9,10-bis­(dicarboximide) pigments. Acta Cryst.
Sect. B.

[115] Fink R. F., Seibt J., Engel V., Renz M., Kaupp M., Lochbrunner S., Zhao H.-M., Pfister J., Würthner F., Engels B. (2008). Exciton Trapping in *π*-Conjugated
Materials: A Quantum-Chemistry-Based Protocol Applied
to Perylene Bisimide Dye Aggregates. J. Am.
Chem. Soc..

[116] Zhao H.-M., Pfister J., Settels V., Renz M., Kaupp M., Dehm V. C., Würthner F., Fink R. F., Engels B. (2009). Understanding Ground- and Excited-State
Properties of Perylene Tetracarboxylic Acid Bisimide Crystals by Means
of Quantum Chemical Computations. J. Am. Chem.
Soc..

[117] Vura-Weis J., Ratner M. A., Wasielewski M. R. (2010). Geometry
and Electronic Coupling in Perylenediimide Stacks: Mapping Structure-Charge
Transport Relationships. J. Am. Chem. Soc..

[118] Hohenstein E. G., Sherrill C. D. (2010). Density fitting
of intramonomer correlation
effects in symmetry-adapted perturbation theory. J. Chem. Phys..

[119] Kodrycka M., Patkowski K. (2019). Platinum,
gold, and silver standards
of intermolecular interaction energy calculations. J. Chem. Phys..

[120] Korona, T. ; Hapka, M. ; Pernal, K. ; Patkowski, K. How to make symmetry-adapted perturbation theory more accurate?, Advances in quantum chemistry, Musiał, M. ; Grabowski, I. Eds.; Academic Press: Cambridge, MA, 2023; Vol. 87, pp. 37–72

[121] Jensen J. H., Gordon M. S. (1996). An approximate formula
for the intermolecular
Pauli repulsion between closed shell molecules. Mol. Phys..

[122] Schäffer R., Jansen G. (2013). Single-determinant-based symmetry-adapted
perturbation theory without single-exchange approximation. Mol. Phys..

[123] Su P., Li H. (2009). Energy decomposition analysis of
covalent bonds and
intermolecular interactions. J. Chem. Phys..

[124] Ziegler T., Rauk A. (1977). On the calculation of bonding energies
by the Hartree Fock Slater method. Theoret.
Chim. Acta.

[125] Bagus P. S., Illas F. (1992). Decomposition of the chemisorption
bond by constrained variations: Order of the variations and construction
of the variational spaces. J. Chem. Phys..

[126] Boys S., Bernardi F. (1970). The calculation of
small molecular
interactions by the differences of separate total energies. Some procedures
with reduced errors. Mol. Phys..

[127] van Duijneveldt-van de Rijdt J. G., van Duijneveldt F. B. (1972). Double-exchange
contributions to the first-order interaction energy between closed-shell
molecules. Chem. Phys. Lett..

[128] Slater, J. C. Quantum Theory of Atomic Structure; McGraw-Hill Inc.: New York, 1960.

[129] Salem L. (1961). The forces between polyatomic molecules. II. Short-range
repulsive
forces. Proc. R. Soc. London, Ser. A.

[130] Naseem-Khan S., Lagardére L., Narth C., Cisneros G. A., Ren P., Gresh N., Piquemal J.-P. (2022). Development of the Quantum-Inspired
SIBFA Many-Body Polarizable Force Field: Enabling Condensed-Phase
Molecular Dynamics Simulations. J. Chem. Theory
Comput..

[131] Papajak E., Truhlar D. G. (2011). Convergent Partially Augmented Basis
Sets for Post-Hartree-Fock Calculations of Molecular Properties and
Reaction Barrier Heights. J. Chem. Theory Comput..

[132] Smith D. G. A., Burns L. A., Simmonett A. C., Parrish R. M., Schieber M. C., Galvelis R., Kraus P., Kruse H., Di Remigio R., Alenaizan A. (2020). PSI4 1.4: Open-source software for high-throughput quantum chemistry. J. Chem. Phys..

[133] Behnle S., Richter R., Völkl L., Idzko P., Förstner A., Bozkaya U., Fink R. F. (2022). Accurate
Property Prediction by Second Order Perturbation Theory: The REMP
and OO-REMP Hybrids. J. Chem. Phys..

[134] MATLAB. Designed for the way you think and the work you do; https://www.mathworks.com/products/matlab.html, accessed 23.03.2025.

[135] McKinney, W. Data Structures for Statistical Computing in Python Proceedings Of The 9th Python In Science Conference SCIPY 2010 56–61

[136] Reback, J. ; McKinney, W. ; Van Den Bossche, J. ; Augspurger, T. ; Cloud, P. ; Klein, A. ; Hawkins, S. ; Roeschke, M. ; Tratner, J. ; She, C. pandas-dev/pandas: Pandas 1.1.5; Zenodo, 2020.

[137] Harris C. R. (2020). Array programming with
NumPy. Nature.

[138] Hunter J. D. (2007). Matplotlib: A 2D Graphics Environment. Comput. Sci. Eng..

[139] Caswell, T. A. ; Droettboom, M. ; Lee, A. ; de Andrade, E. S. ; Hunter, J. ; Firing, E. ; Hoffmann, T. ; Klymak, J. ; Stansby, D. ; Varoquaux, N. matplotlib/matplotlib: REL: v3.3.4; Zenodo, 2021.

[140] gnuplot. gnuplot homepage; https://gnuplot.sourceforge.net/, accessed 23.03.2025.

[141] CorelDraw. CorelDraw homepage; https://www.coreldraw.com/, accessed 26.11.2025.

[142] MOLDEN. MOLDEN a pre- and post processing program of molecular and electronic structure; https://www.theochem.ru.nl/molden/, accessed 21.03.2025. 10.1023/a:1008193805436 10721501

[143] Grimme S. (2008). Do Special Noncovalent *π*-*π* Stacking Interactions Really Exist?. Angew. Chem., Int. Ed..

[144] Mulliken R. S. (1949). Report on molecular orbital theory. J. Chim. Phys..

[145] For the exchange integral MOPCE contribution we have *E* _xi_(*a*,*b*)=-2(*ab*|*ba*) and the Mulliken approximation for the latter integral reads $\left(ab|ba\right)\approx \frac{1}{4}S_{ab}^{2}\left[\left(aa|aa\right)+2\left(aa|bb\right)+\left(bb|bb\right)\right]$ .

[146] Coulson C. A. (1948). Excited
Electronic Levels in Conjugated Molecules:
I. Long Wavelength Ultra-Violet Absorption of Naphthalene, Anthracene
and Homologs. Proc. Phys. Soc..

[147] Salem L., Longuet-Higgins H. C. (1960). The alternation
of bond lengths in
long conjugated molecules. - II. The polyacenes. Proc. R. Soc. London, Ser. A.

[148] Bernal J. D. (1924). The structure of graphite. Proc.
R. Soc. London A.

[149] Ponder J. W., Wu C., Ren P., Pande V. S., Chodera J. D., Schnieders M. J., Haque I., Mobley D. L., Lambrecht D. S., DiStasio R. A., Head-Gordon M., Clark G. N. I., Johnson M. E., Head-Gordon T. (2010). Current Status
of the AMOEBA Polarizable Force Field. J. Phys.
Chem. B.

[150] Strutyński K., Gomes J. A. N. F., Melle-Franco M. (2014). Accuracy of
Dispersion Interactions in Semiempirical and Molecular Mechanics Models:
The Benzene Dimer Case. J. Phys. Chem. A.

[151] Van Vleet M. J., Misquitta A. J., Schmidt J. R. (2018). New Angles on Standard
Force Fields: Toward a General Approach for Treating Atomic-Level
Anisotropy. J. Chem. Theory Comput..

