# Supplementary material for: The Nodal Structure of π‑Orbitals Is Mapped in the Interaction Energy of π‑Stacked Acene Dimers
Source: J Am Chem Soc. 2026 Apr 9;148(15):16151–69. doi: 10.1021/jacs.6c00921 (PMC13107468; doi:10.1021/jacs.6c00921)
Supplement: Supplementary file 2 [file ja6c00921_si_002.pdf]

Supporting Information

The nodal structure of π-orbitals is mapped in the interaction energy of π-stacked acene dimers

Michael Thelen, Johannes F. Henrichsmeyer, Andrea Buchwald, and Reinhold F. Fink*

Institute of Physical and Theoretical Chemistry, Auf der Morgenstelle 18, Universität Tübingen, D-72076 Tübingen, Germany

E-mail: Reinhold.Fink@uni-tuebingen.de

The figures and tables referred to in the manuscript can be found in this document below. The underlying numerical values are collected in the zip-file `data_si.zip`, which contains ASCII files in the csv (comma-separated value) format. The file names are of the type `{molecule}_{method}_{shift}.csv` where `{molecule}` refers to the acene (`benzene`, `naphthalene`, `anthracene`, `tetracene`, or `pentacene`). `{method}` is either `sapt` for the SAPT0/jun-cc-pVDT results or `mopce` for MOPCE data, which provide the exchange repulsion energy as well as its individual terms according to eq. (4) and the π-π, π-σ, and σ-σ contributions. `shift` can be `xshift`, `yshift`, `y1_xshift` for x-shift with Δy=1 Å, or `2D` for a two dimensional surface. Furthermore, `pentacene_mopce_xshift_orbitalcontributions.csv` contains MOPCE energies for each of the individual π-π contributions.

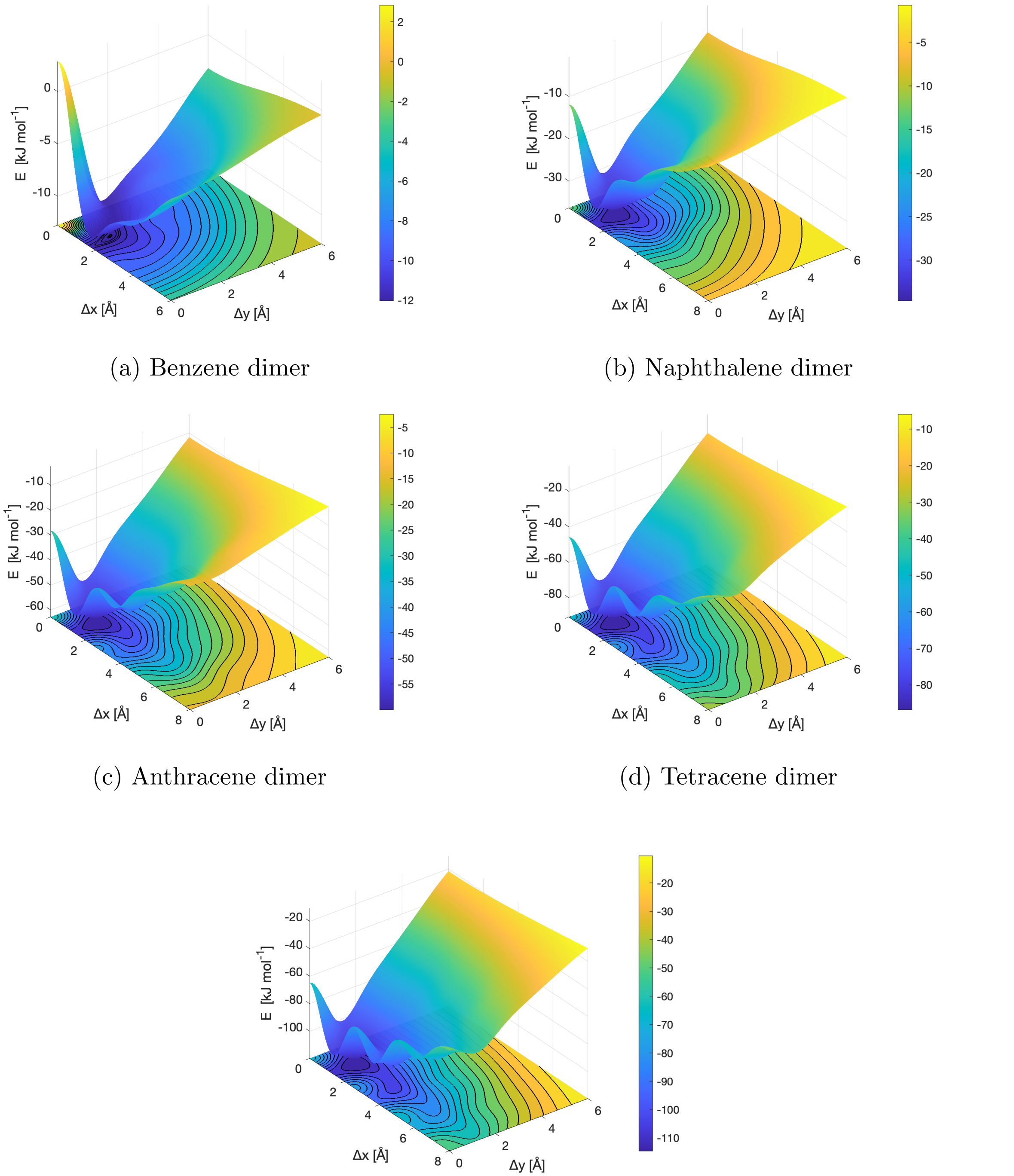

(a) Benzene dimer

(b) Naphthalene dimer

(c) Anthracene dimer

(d) Tetracene dimer

(e) Pentacene dimer

Figure S1: Interaction energy E_{int} of parallel displaced acenes (as indicated below each figure) for $\Delta z = 3.4\,\text{Å}$. The results were obtained at the SAPT0/jun-cc-pVDZ level of theory.

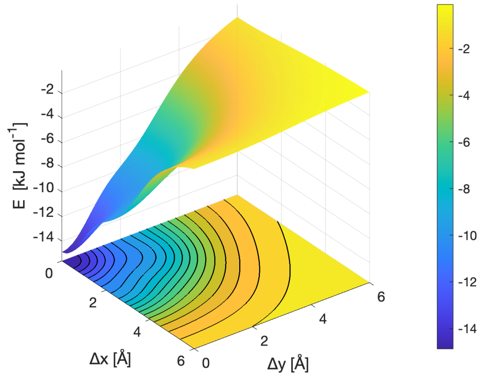

(a) Benzene dimer

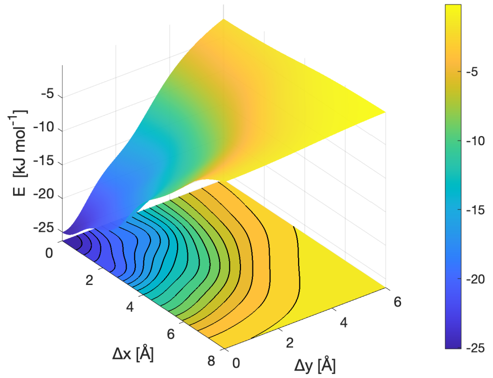

(b) Naphthalene dimer

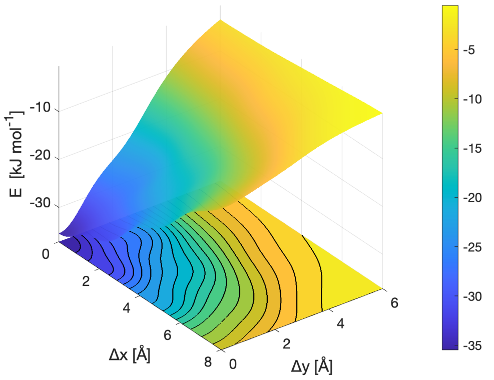

(c) Anthracene dimer

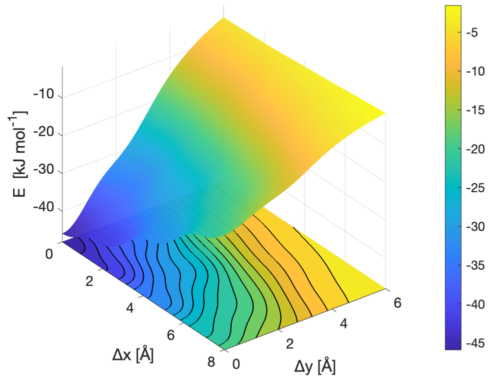

(d) Tetracene dimer

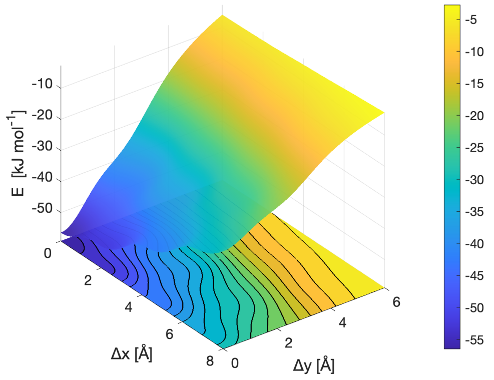

(e) Pentacene dimer

Figure S2: Electrostatic energy contribution E_{el} to the total interaction energy E_{int} of parallel displaced acenes (as indicated below each figure) for $\Delta z = 3.4\,\text{Å}$. The results were obtained at the SAPT0/jun-cc-pVDZ level of theory.

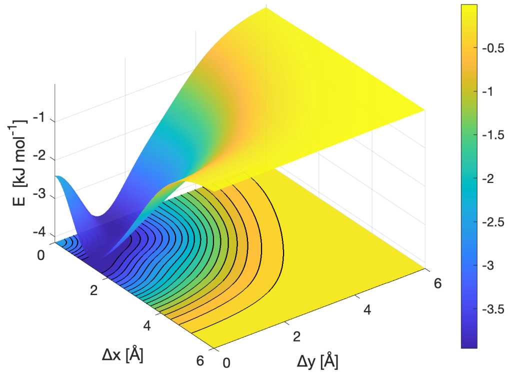

(a) Benzene dimer

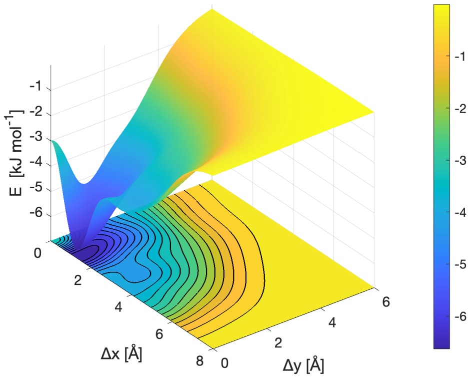

(b) Naphthalene dimer

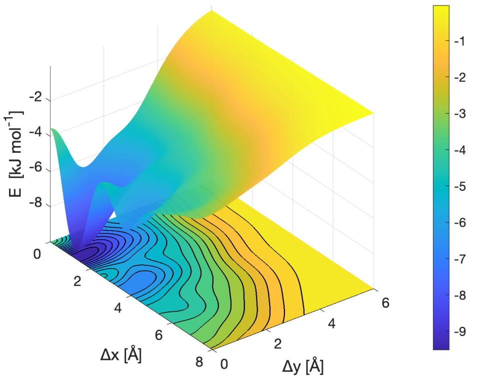

(c) Anthracene dimer

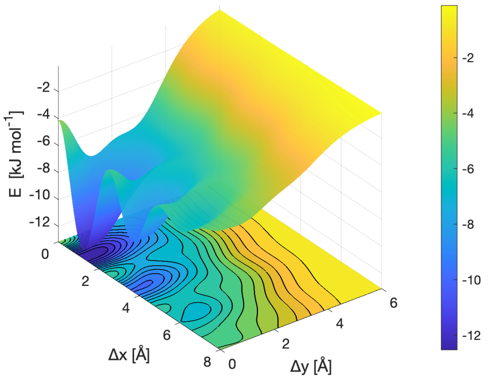

(d) Tetracene dimer

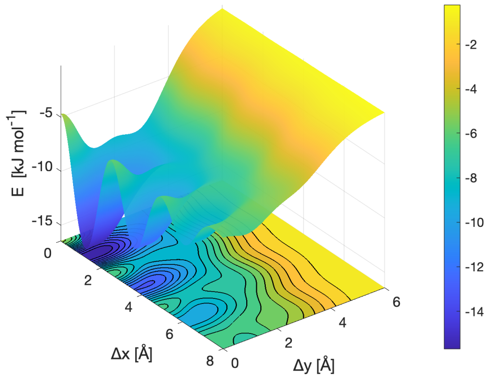

(e) Pentacene dimer

Figure S3: Induction energy contribution E_{ind} to the total interaction energy E_{int} of parallel displaced acenes (as indicated below each figure) for $\Delta z = 3.4$ Å. The results were obtained at the SAPT0/jun-cc-pVDZ level of theory.

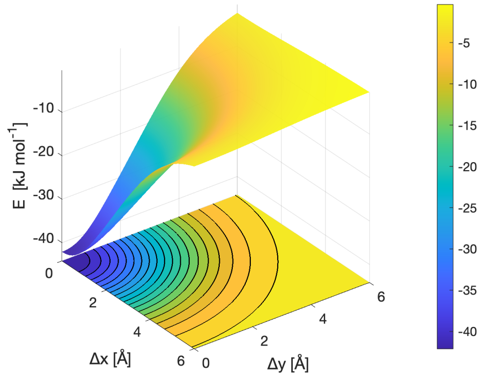

(a) Benzene dimer

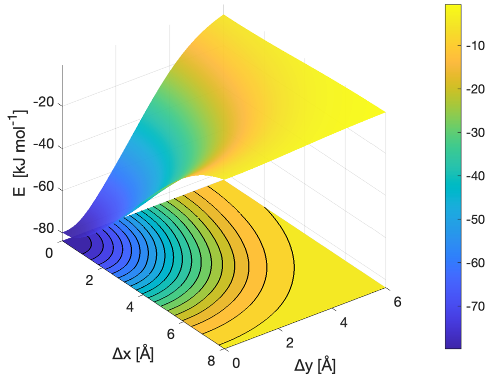

(b) Naphthalene dimer

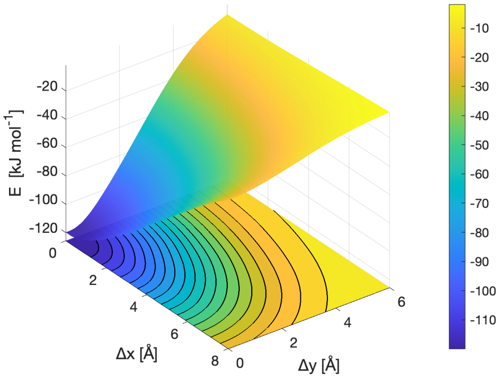

(c) Anthracene dimer

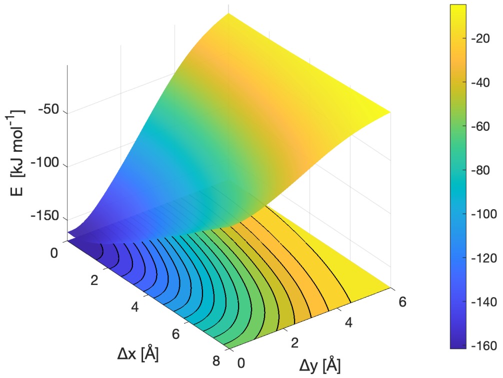

(d) Tetracene dimer

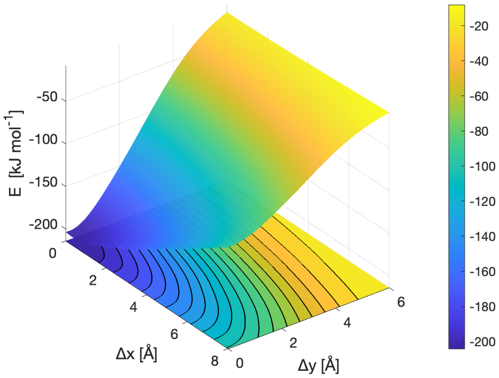

(e) Pentacene dimer

Figure S4: Dispersion energy contribution E_{dsp} to the total interaction energy E_{int} of parallel displaced acenes (as indicated below each figure) for $\Delta z = 3.4\,\text{Å}$. The results were obtained at the SAPT0/jun-cc-pVDZ level of theory.

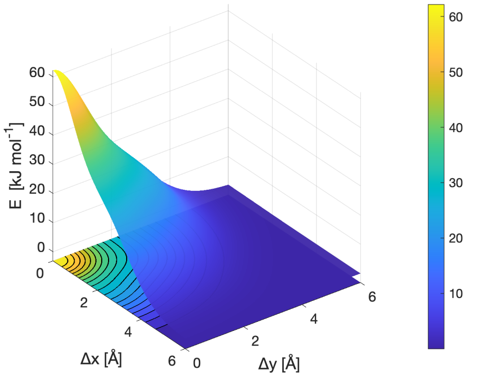

(a) Benzene dimer

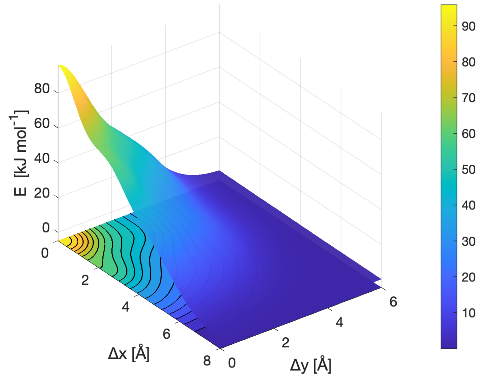

(b) Naphthalene dimer

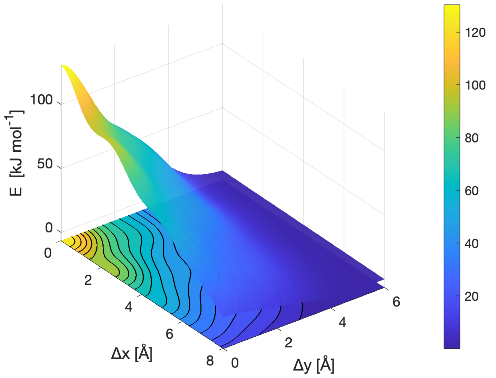

(c) Anthracene dimer

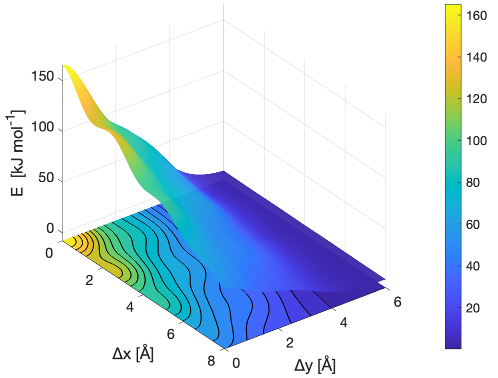

(d) Tetracene dimer

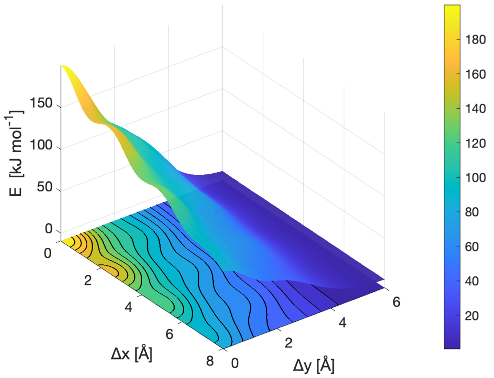

(e) Pentacene dimer

Figure S5: Exchange repulsion energy contribution E_{exch} to the total interaction energy E_{int} of parallel displaced acenes (as indicated below each figure) for $\Delta z = 3.4\,\text{Å}$. The results were obtained at the SAPT0/jun-cc-pVDZ level of theory.

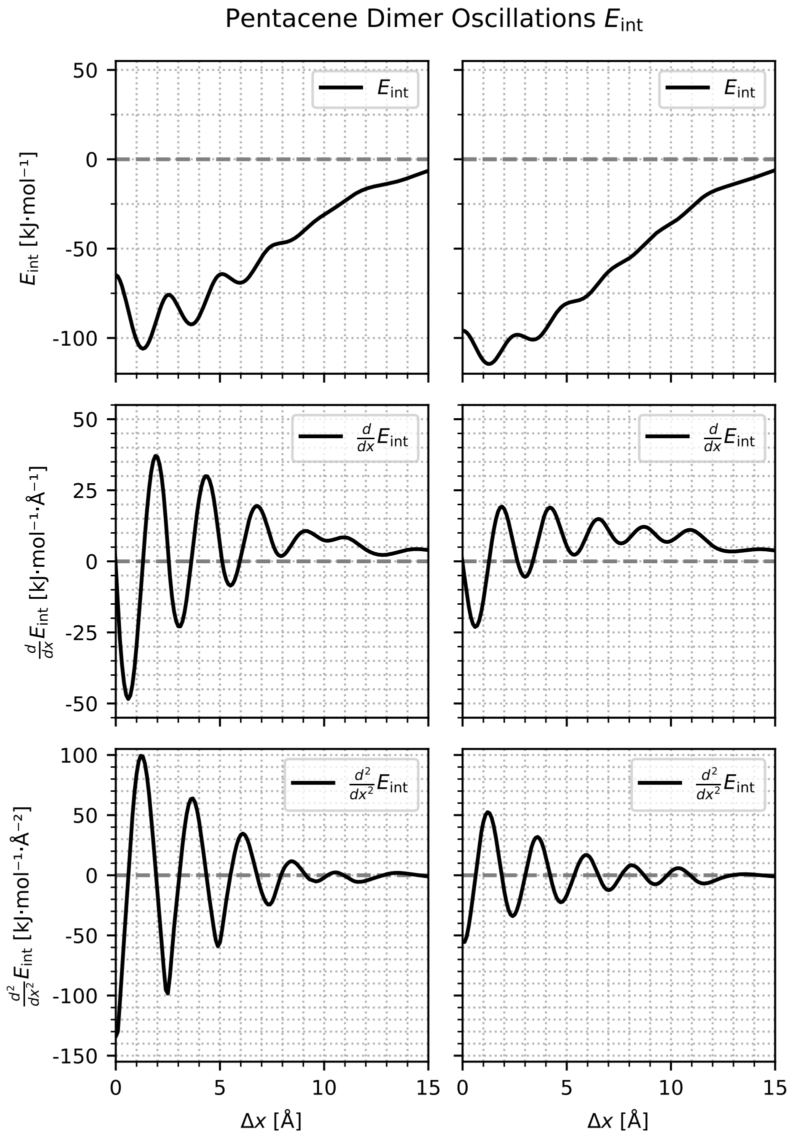

Figure S6: The total interaction energy, E_{int}, for an x-shift at $\Delta y = 0$ (left column) and at $\Delta y = 1$Å (right), as well as its first and second derivative (second and third row).

Figs. S6 and S7 show the SAPT0 interaction energy and the exchange repulsion energy, respectively, of the Pen_2-system as a function of the x-shift for Δy=0 and 1 Å. E_{exch} shown in Fig. S7 decays strongly with increasing Δx, which makes it difficult to identify an oscillating structure. Such a structure is easier to identify in Fig. S6 which shows the interaction

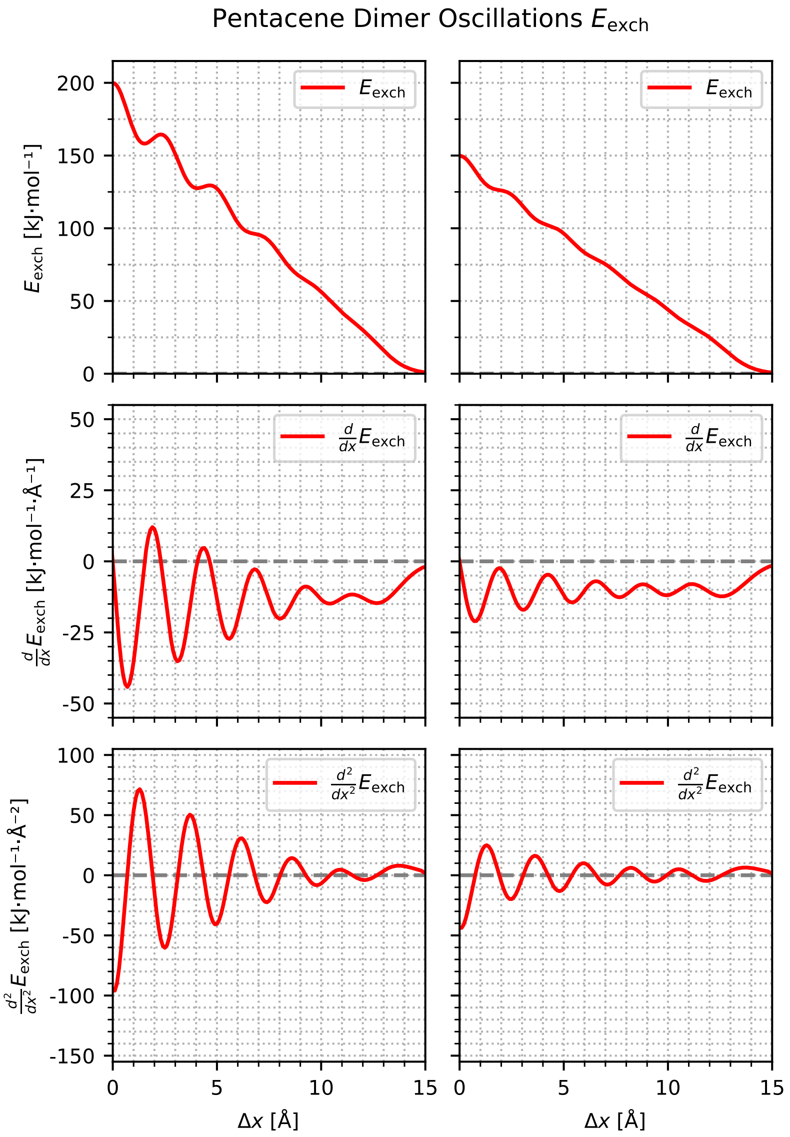

Figure S7: The SAPT exchange repulsion energy, E_{exch}, for an x-shift at $\Delta y = 0$ (left column) and at $\Delta y = 1$Å (right), as well as its first and second derivative (second and third row).

energies as a function of Δx. The first and second derivatives of these energy curves which are presented in the second and third row of these figures show significant similarities. This demonstrates that the structure of the interaction energy is actually caused by oscillations

in the exchange repulsion energy.

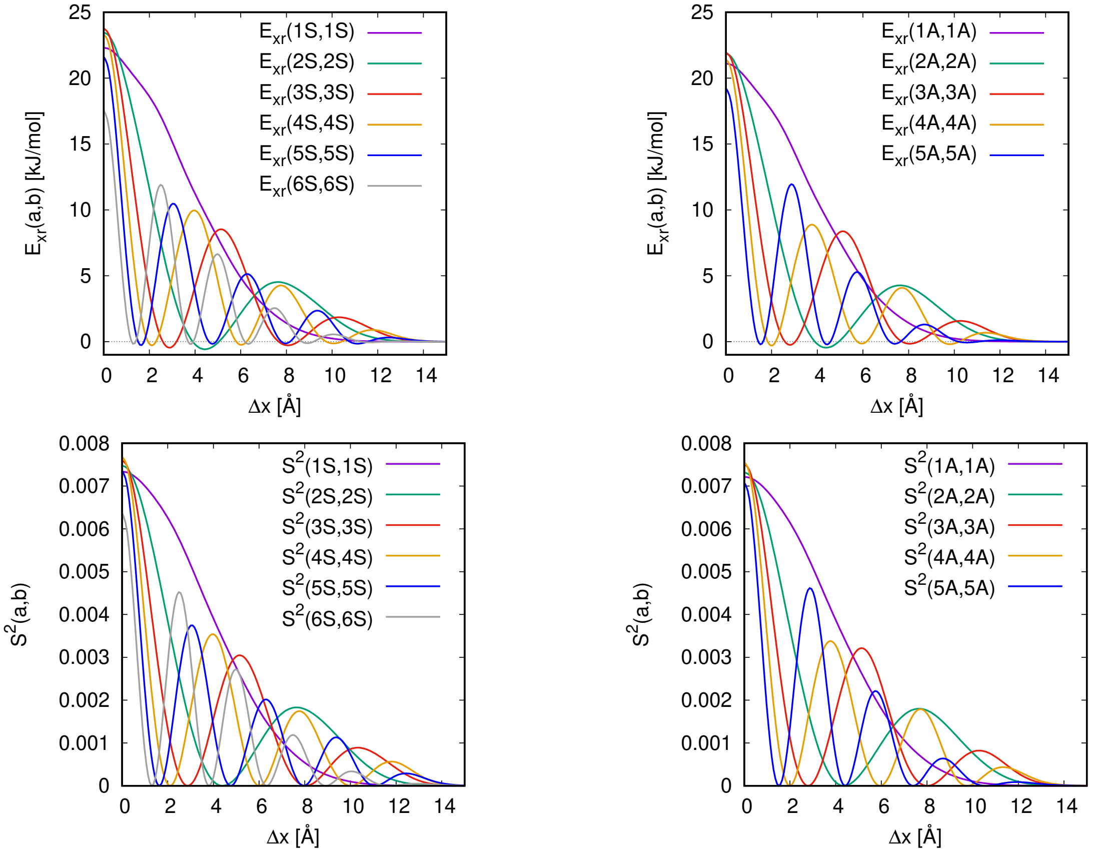

Figure S8: Diagonal orbital-pair contributions $E_{\mathrm{xr}}(m\mathrm{S}, n\mathrm{S})$ and $E_{\mathrm{xr}}(m\mathrm{A}, n\mathrm{A})$ to $E_{\mathrm{xr}}(\pi\text{-}\pi)$ of the Pen$_2$-system as a function of the x-shift for $n = m$ representing the π-orbitals that are symmetric (S) and antisymmetric (A) to the σ_{xz} plane, respectively. The corresponding squared overlap integrals are also shown.

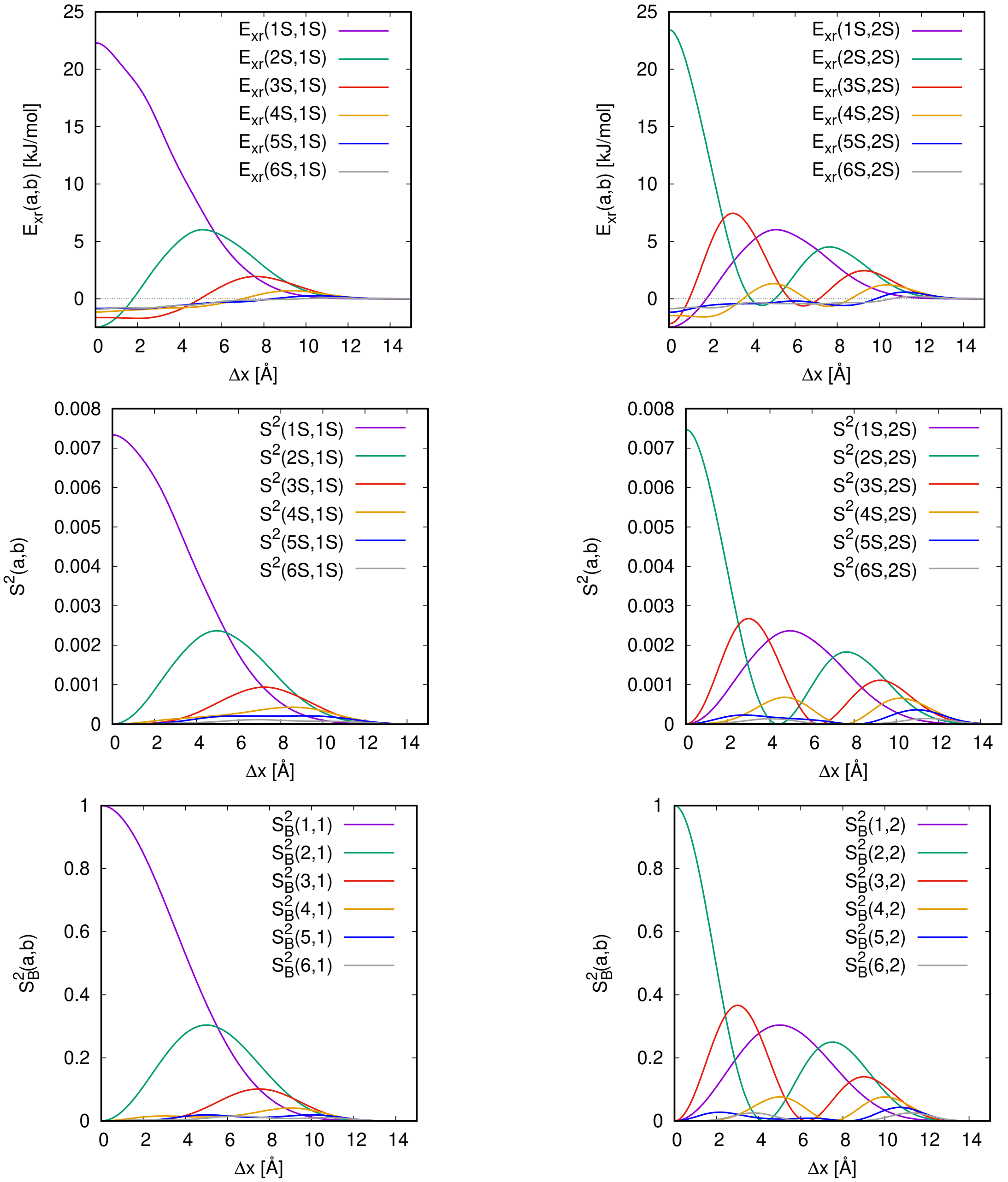

Figure S9: Orbital-pair contributions $E_{\rm xr}(m{\rm S}, n{\rm S})$ to $E_{\rm xr}(\pi\text{-}\pi)$ of the Pen$_2$-system as a function of the x-shift for $n = 1$ and 2 and all m representing the π-orbitals that are symmetric with respect to the σ_{xz} plane. The corresponding squared overlap integrals and the squared overlap integrals of the particle-in-a-box model are also shown.

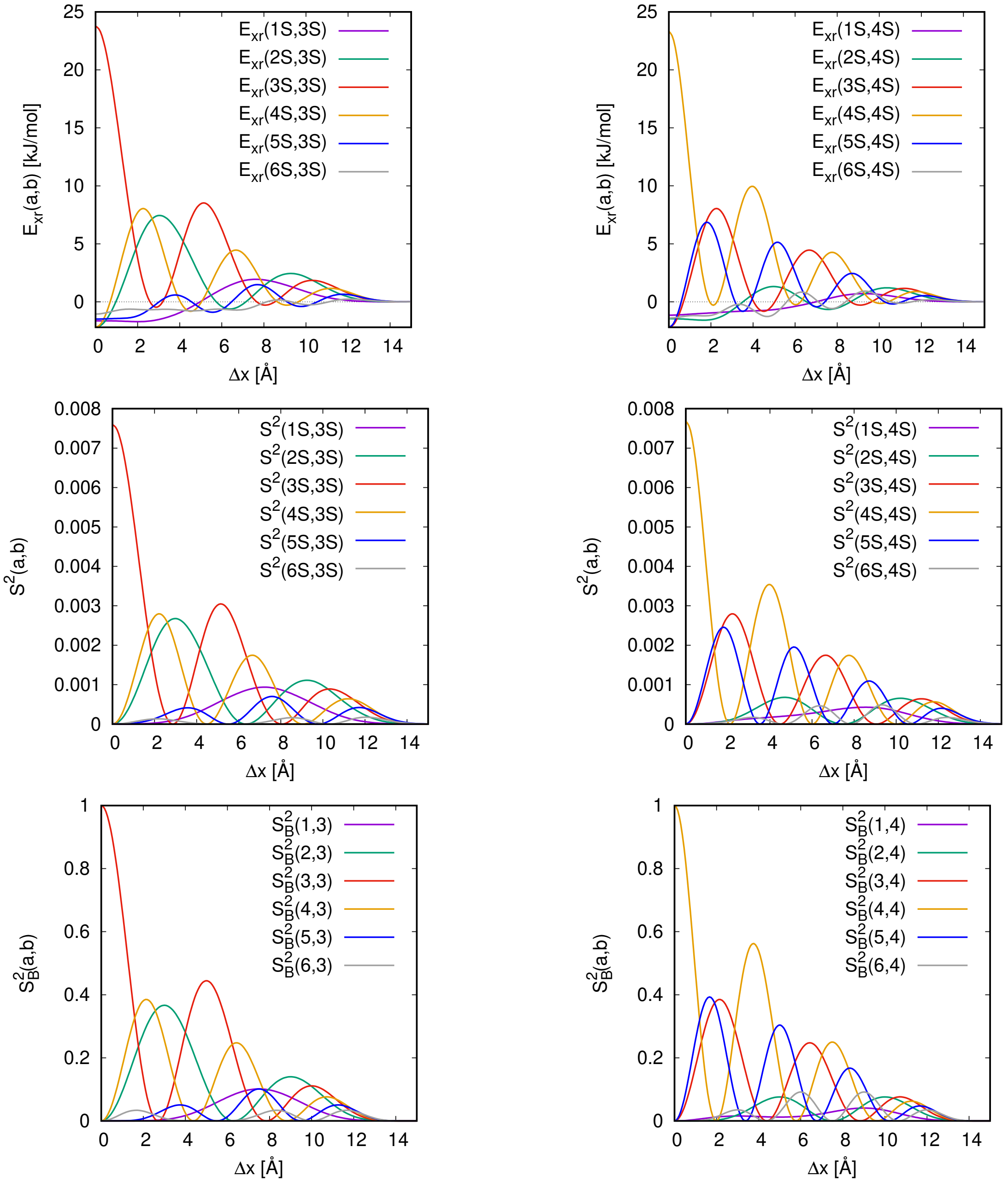

Figure S10: Orbital-pair contributions $E_{\mathrm{xr}}(m\mathrm{S}, n\mathrm{S})$ to $E_{\mathrm{xr}}(\pi\text{-}\pi)$ of the Pen_2-system as a function of the x-shift for $n = 3$ and 4 and all m representing the π-orbitals that are symmetric with respect to the σ_{xz} plane. The corresponding squared overlap integrals and the squared overlap integrals of the particle-in-a-box model are also shown.

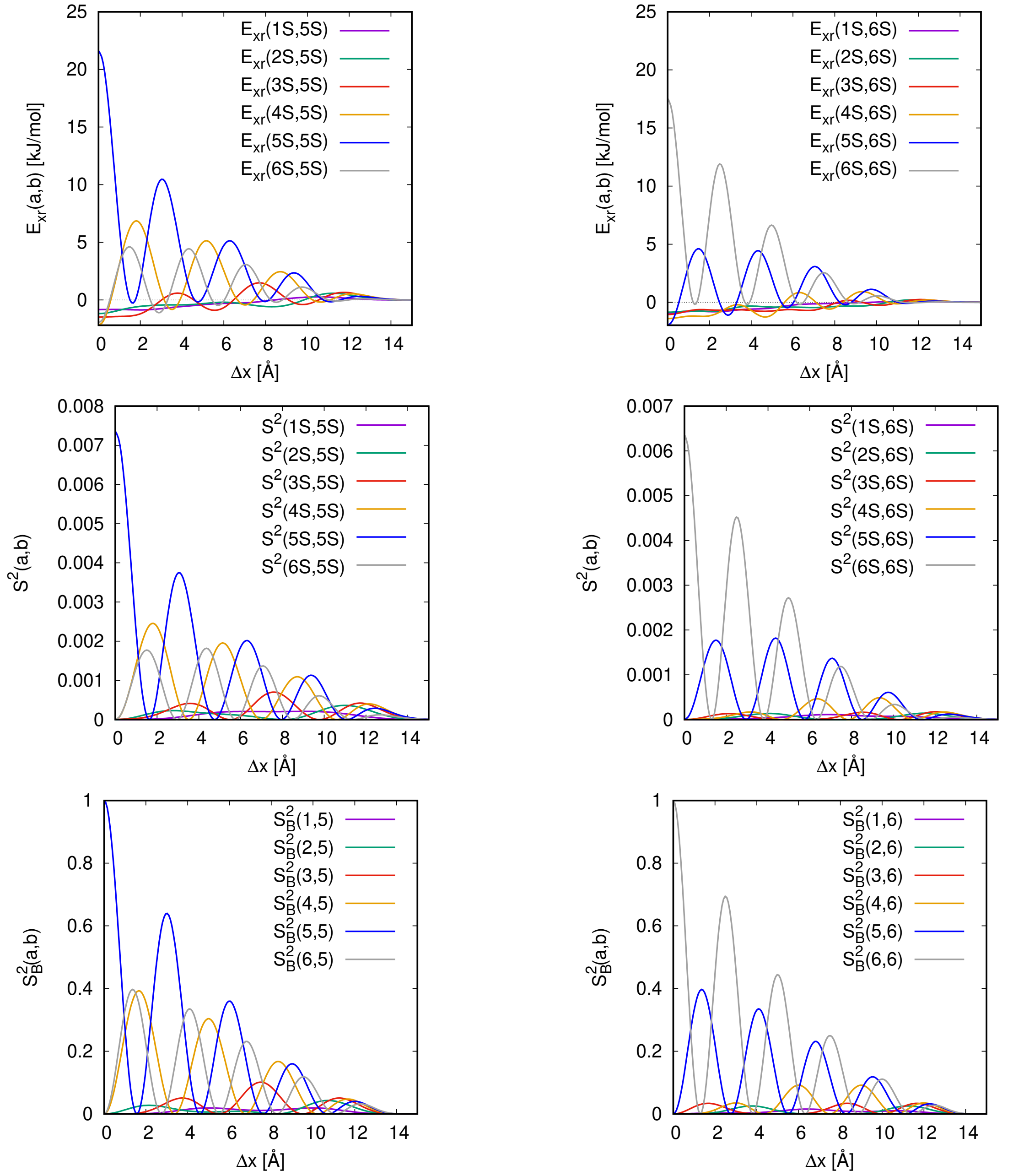

Figure S11: Orbital-pair contributions $E_{\mathrm{xr}}(m\mathrm{S}, n\mathrm{S})$ to $E_{\mathrm{xr}}(\pi\text{-}\pi)$ of the Pen_2-system as a function of the x-shift for $n = 5$ and 6 and all m representing the π-orbitals that are symmetric with respect to the σ_{xz} plane. The corresponding squared overlap integrals and the squared overlap integrals of the particle-in-a-box model are also shown.

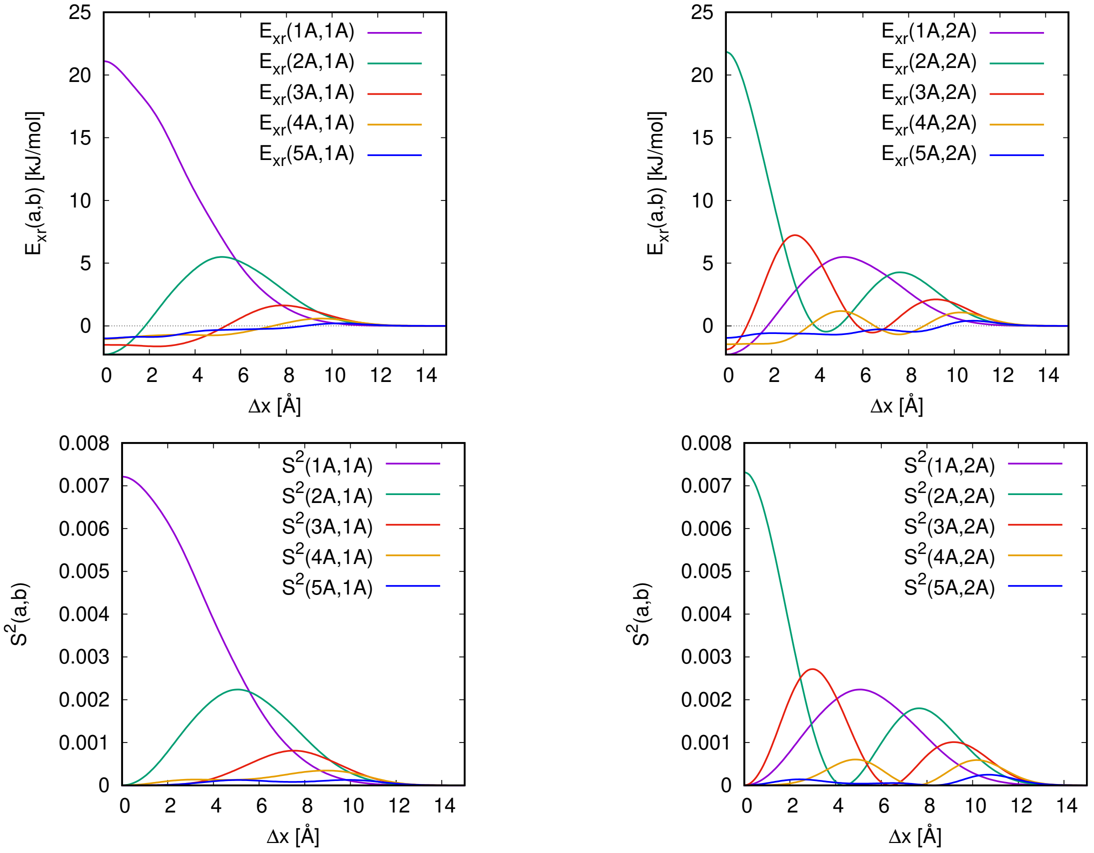

Figure S12: Orbital-pair contributions $E_{\text{xr}}(m\text{A}, n\text{A})$ to $E_{\text{xr}}(\pi\text{-}\pi)$ of the Pen_2-system as a function of the x-shift for $n = 1$ and 2 and all m representing the π-orbitals that are antisymmetric with respect to the σ_{xz} plane. The corresponding squared overlap integrals are also shown.

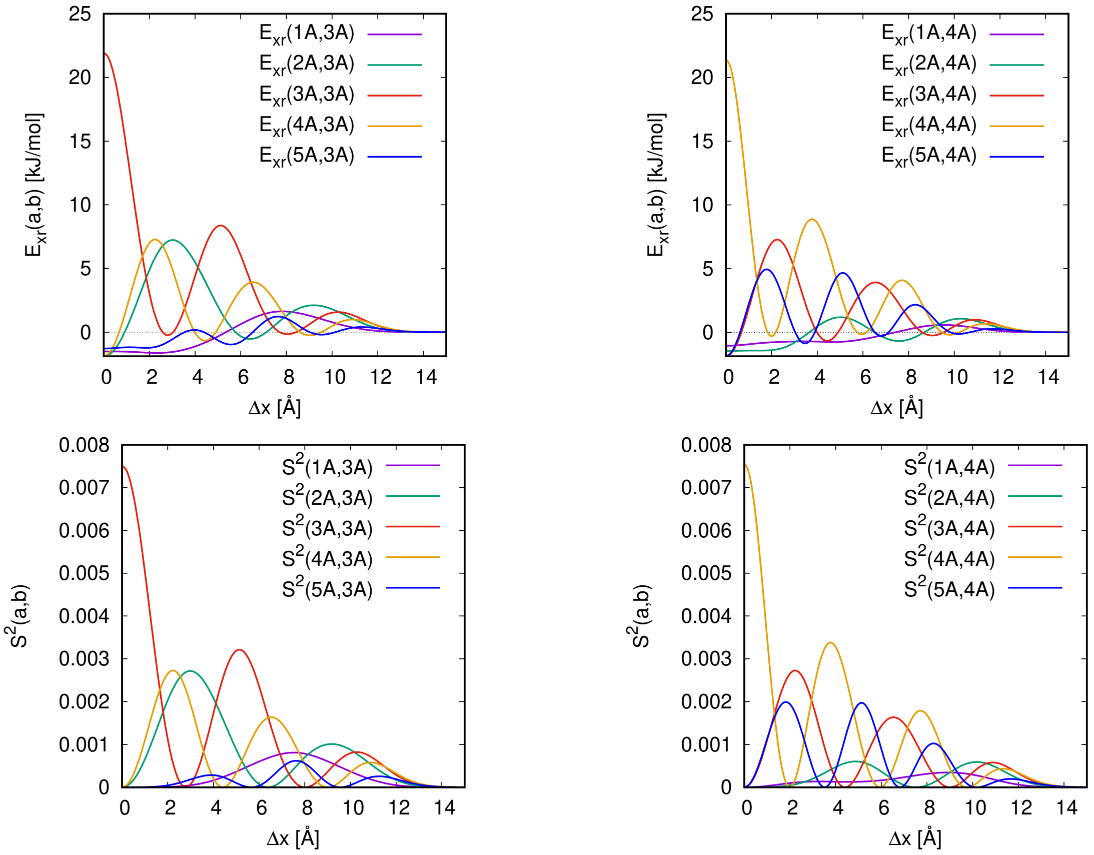

Figure S13: Orbital-pair contributions $E_{\mathrm{xr}}(m\mathrm{A}, n\mathrm{A})$ to $E_{\mathrm{xr}}(\pi\text{-}\pi)$ of the Pen_2-system as a function of the x-shift for $n = 3$ and 4 and all m representing the π-orbitals that are antisymmetric with respect to the σ_{xz} plane. The corresponding squared overlap integrals are also shown.

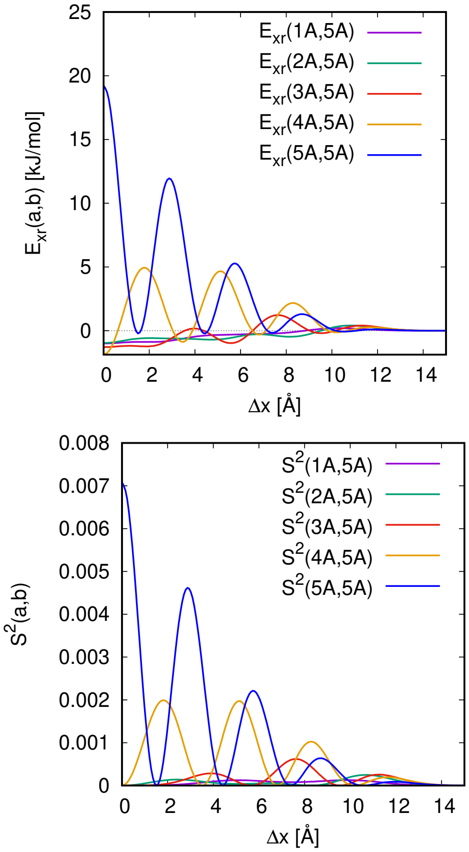

Figure S14: Orbital-pair contributions $E_{\rm xr}(m{\rm A}, n{\rm A})$ to $E_{\rm xr}(\pi\text{-}\pi)$ of the Pen$_2$-system as a function of the x-shift for $n = 5$ and all m representing the π-orbitals that are antisymmetric with respect to the σ_{xz} plane. The corresponding squared overlap integrals are also shown.

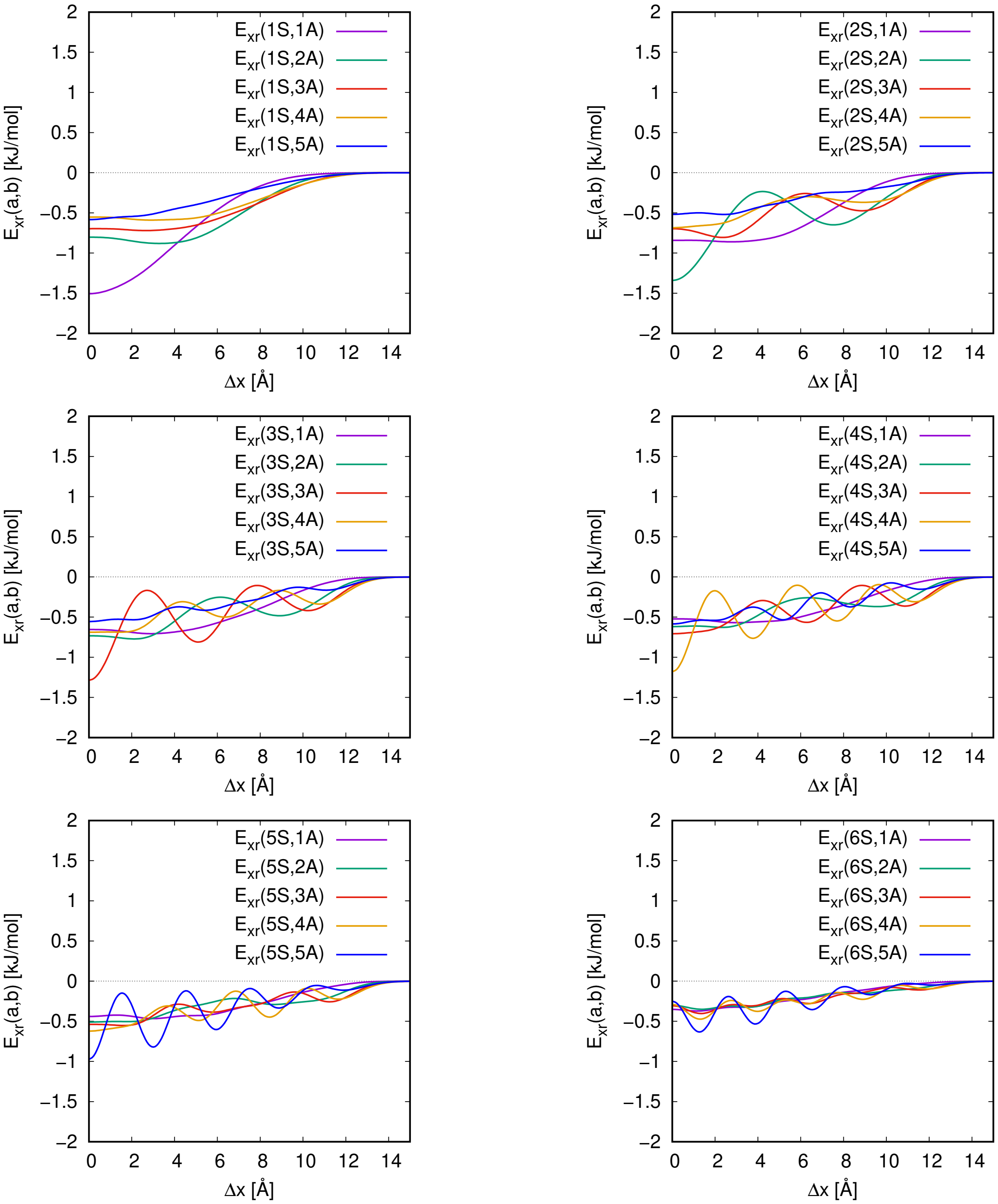

Figure S15: Orbital-pair contributions $E_{\text{xr}}(m\text{S}, n\text{A})$ to $E_{\text{xr}}(\pi\text{-}\pi)$ of the Pen_2-system as a function of the x-shift for all n (m) representing the π-orbitals that are symmetric (anti-symmetric) with respect to the σ_{xz} plane. The corresponding overlap integrals are zero and, thus, not shown.

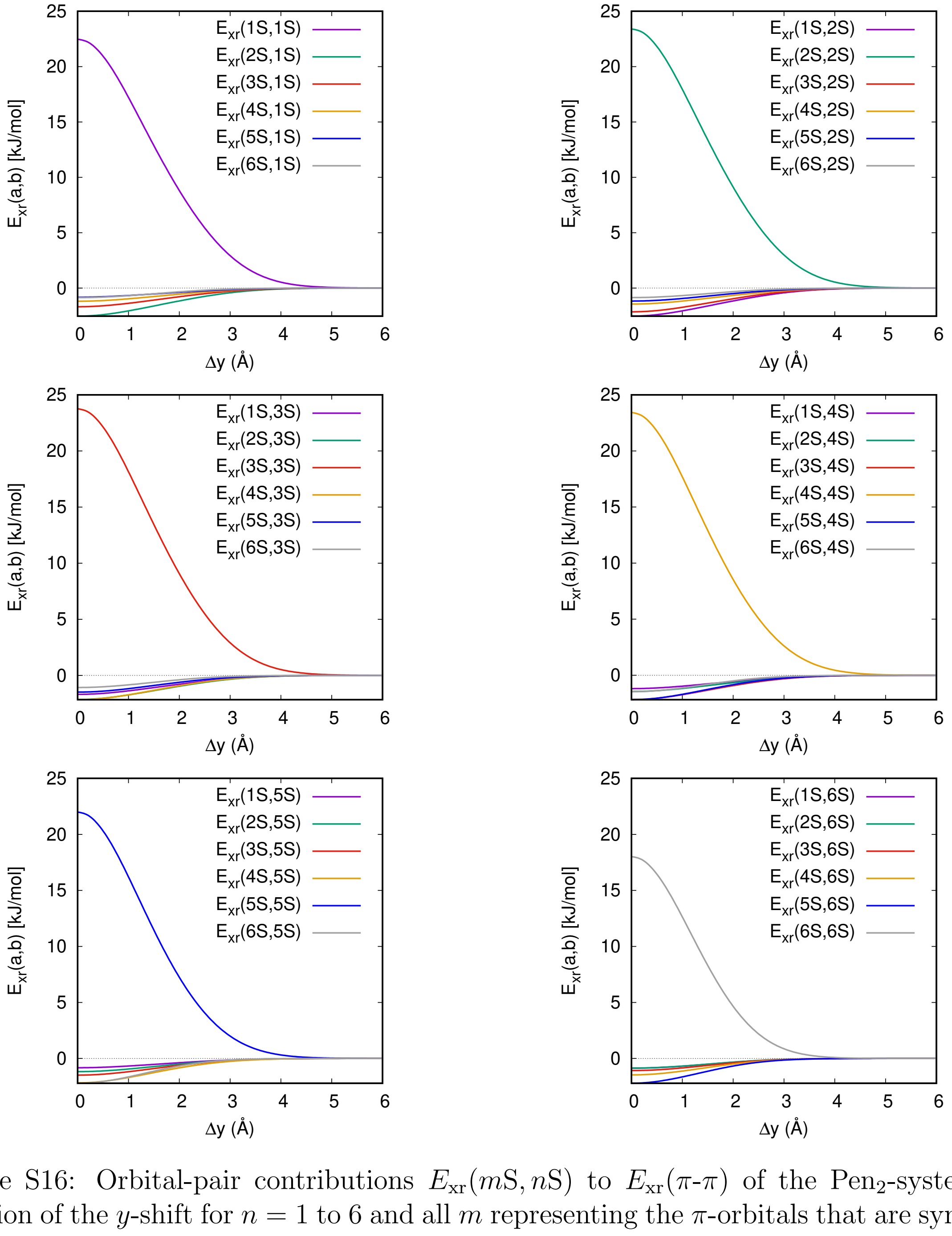

Figure S16: Orbital-pair contributions $E_{\mathrm{xr}}(m\mathrm{S}, n\mathrm{S})$ to $E_{\mathrm{xr}}(\pi\text{-}\pi)$ of the Pen_2-system as a function of the y-shift for $n = 1$ to 6 and all m representing the π-orbitals that are symmetric with respect to the σ_{xz} plane.

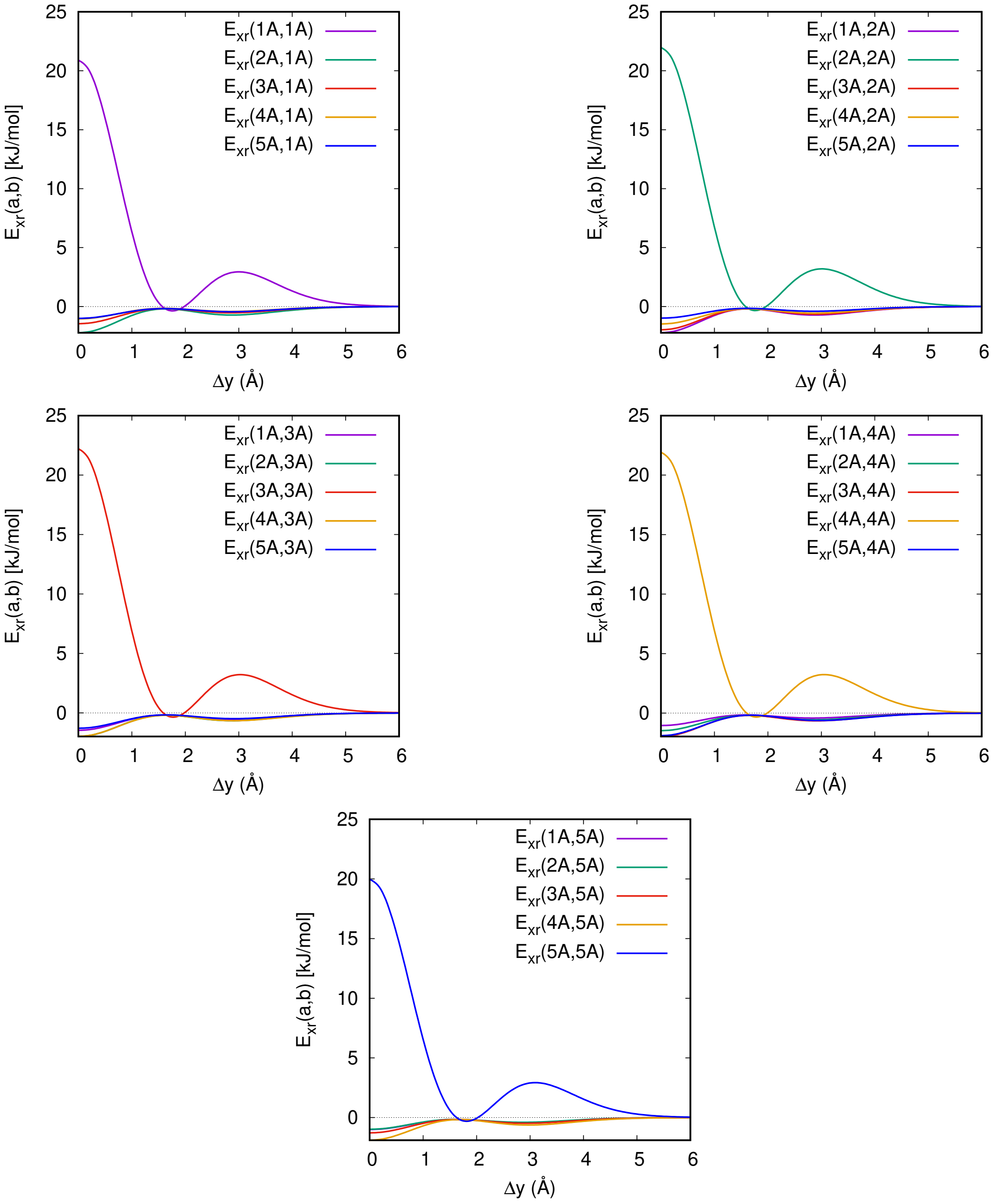

Figure S17: Orbital-pair contributions $E_{\mathrm{xr}}(m\mathrm{A}, n\mathrm{A})$ to $E_{\mathrm{xr}}(\pi\text{-}\pi)$ of the Pen_2-system as a function of the y-shift for $n = 1$ to 5 and all m representing the π-orbitals that are antisymmetric with respect to the σ_{xz} plane.

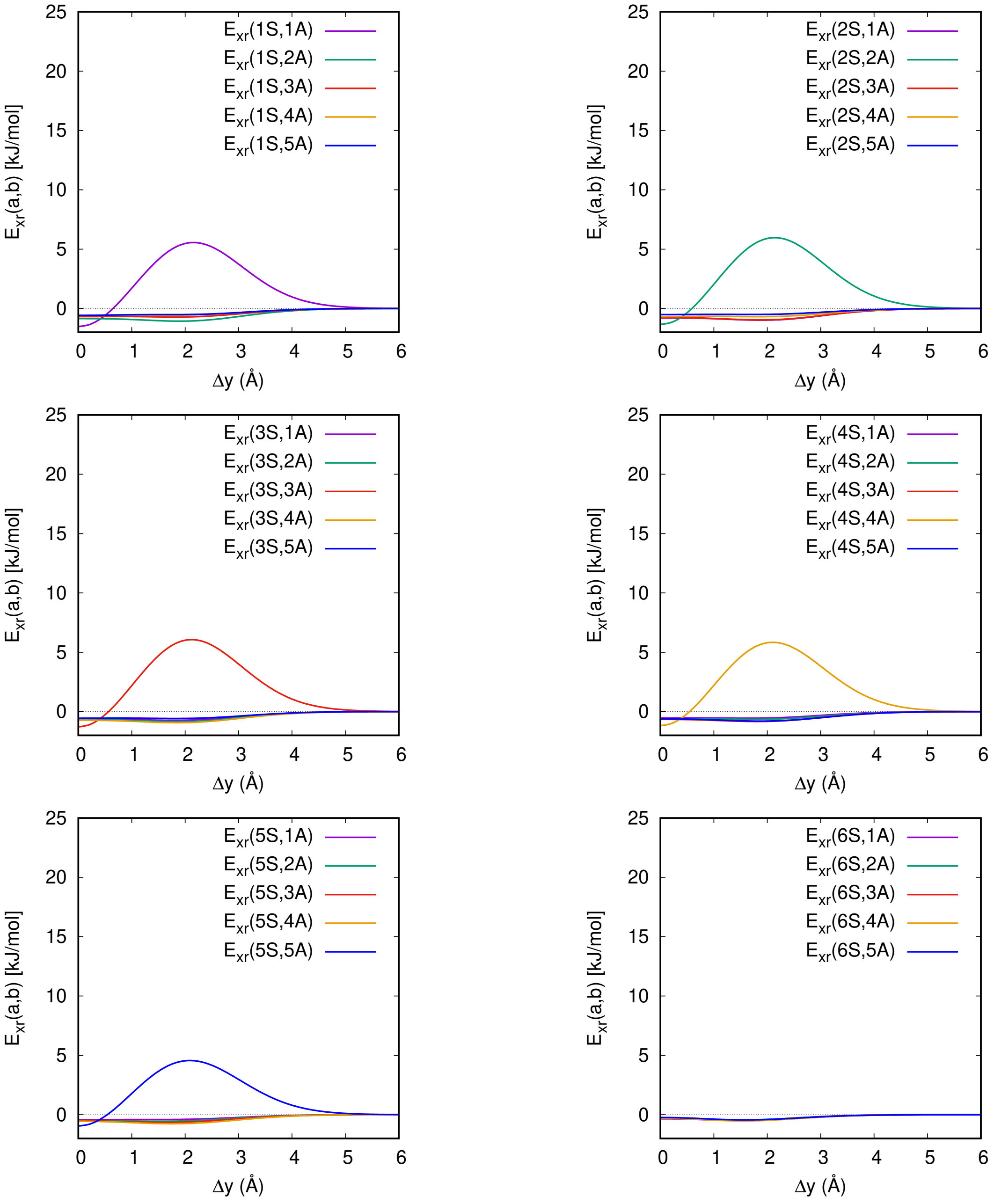

Figure S18: Orbital-pair contributions $E_{\mathrm{xr}}(m\mathrm{S}, n\mathrm{A})$ to $E_{\mathrm{xr}}(\pi\text{-}\pi)$ of the Pen$_2$-system as a function of the y-shift for $m = 1$ to 6 symmetric (S) and $n = 1$ to 5 antisymmetric (A) π-orbitals with respect to the σ_{xz} plane.

Monomer Geometries

The molecular structures of the monomers are collected below and additionally available as xyz-files in the data-si.zip file in the ESI.

Table S1: Benzene monomer geometry obtained with RI-MP2/aug-cc-pVTZ level of theory using D_{6h} symmetry.

Element	x [Å]	y [Å]	z [Å]
C	-0.6971	1.2074	0.0000
C	0.6971	1.2074	0.0000
C	-1.3942	-0.0000	0.0000
C	1.3942	-0.0000	0.0000
C	-0.6971	-1.2074	0.0000
C	0.6971	-1.2074	0.0000
H	-1.2382	2.1446	0.0000
H	1.2382	2.1446	0.0000
H	-2.4764	-0.0000	0.0000
H	2.4764	-0.0000	0.0000
H	-1.2382	-2.1446	0.0000
H	1.2382	-2.1446	0.0000

Table S2: Naphthalene monomer geometry obtained with RI-MP2/aug-cc-pVTZ level of theory using D_{2h} symmetry.

Element	x [Å]	y [Å]	z [Å]
C	2.4168	0.7030	0.0000
C	1.2317	1.3946	0.0000
C	0.0000	0.7118	0.0000
C	-1.2317	1.3946	0.0000
C	2.4168	-0.7030	0.0000
H	3.3547	1.2377	0.0000
H	1.2304	2.4757	0.0000
H	-1.2304	2.4757	0.0000
H	3.3547	-1.2377	0.0000
C	0.0000	-0.7118	0.0000
C	-2.4168	0.7030	0.0000
C	1.2317	-1.3946	0.0000
C	-1.2317	-1.3946	0.0000
C	-2.4168	-0.7030	0.0000
H	-3.3547	1.2377	0.0000
H	1.2304	-2.4757	0.0000
H	-1.2304	-2.4757	0.0000
H	-3.3547	-1.2377	0.0000

Table S3: Anthracene monomer geometry obtained with RI-MP2/aug-cc-pVTZ level of theory using D_{2h} symmetry.

Element	x [Å]	y [Å]	z [Å]
C	3.6336	0.7066	0.0000
C	2.4541	1.3988	0.0000
C	1.2149	0.7170	0.0000
C	1.2149	-0.7170	0.0000
C	2.4541	-1.3988	0.0000
C	3.6336	-0.7066	0.0000
C	0.0000	1.3969	0.0000
C	0.0000	-1.3969	0.0000
C	-1.2149	-0.7170	0.0000
C	-1.2149	0.7170	0.0000
C	-2.4541	1.3988	0.0000
H	-2.4540	2.4804	0.0000
C	-3.6336	0.7066	0.0000
C	-3.6336	-0.7066	0.0000
C	-2.4541	-1.3988	0.0000
H	0.0000	2.4805	0.0000
H	4.5728	1.2396	0.0000
H	2.4540	2.4804	0.0000
H	2.4540	-2.4804	0.0000
H	4.5728	-1.2396	0.0000
H	0.0000	-2.4805	0.0000
H	-4.5728	1.2396	0.0000
H	-4.5728	-1.2396	0.0000
H	-2.4540	-2.4804	0.0000

Table S4: Tetracene monomer geometry obtained with RI-MP2/aug-cc-pVTZ level of theory using D_{2h} symmetry.

Element	x [Å]	y [Å]	z [Å]
C	0.0000	0.7221	0.0000
C	0.0000	-0.7221	0.0000
C	1.2226	1.4002	0.0000
C	-1.2226	1.4002	0.0000
C	1.2226	-1.4002	0.0000
C	-1.2226	-1.4002	0.0000
C	2.4324	0.7187	0.0000
C	-2.4324	0.7187	0.0000
C	2.4324	-0.7187	0.0000
C	-2.4324	-0.7187	0.0000
C	3.6745	1.3999	0.0000
C	-3.6745	1.3999	0.0000
C	3.6745	-1.3999	0.0000
C	-3.6745	-1.3999	0.0000
C	4.8523	0.7080	0.0000
C	-4.8523	0.7080	0.0000
C	4.8523	-0.7080	0.0000
C	-4.8523	-0.7080	0.0000
H	1.2247	2.4842	0.0000
H	-1.2247	2.4842	0.0000
H	1.2247	-2.4842	0.0000
H	-1.2247	-2.4842	0.0000
H	3.6749	2.4816	0.0000
H	-3.6749	2.4816	0.0000
H	3.6749	-2.4816	0.0000
H	-3.6749	-2.4816	0.0000
H	5.7919	1.2406	0.0000
H	-5.7919	1.2406	0.0000
H	5.7919	-1.2406	0.0000
H	-5.7919	-1.2406	0.0000

Table S5: Pentacene monomer geometry obtained with RI-MP2/aug-cc-pVTZ level of theory using D_{2h} symmetry.

Element	x [Å]	y [Å]	z [Å]
C	4.8947	1.4001	0.0000
C	3.6522	0.7187	0.0000
C	3.6522	-0.7187	0.0000
C	4.8947	-1.4001	0.0000
C	6.0724	-0.7083	0.0000
C	6.0724	0.7083	0.0000
C	2.4429	1.4010	0.0000
C	1.2182	0.7240	0.0000
C	1.2182	-0.7240	0.0000
C	2.4429	-1.4010	0.0000
C	-0.0000	1.4040	0.0000
C	-1.2182	0.7240	0.0000
C	-1.2182	-0.7240	0.0000
C	0.0000	-1.4040	0.0000
C	-2.4429	1.4010	0.0000
C	-3.6522	0.7187	0.0000
C	-3.6522	-0.7187	0.0000
C	-2.4429	-1.4010	0.0000
C	-4.8947	1.4001	0.0000
C	-6.0724	0.7083	0.0000
C	-6.0724	-0.7083	0.0000
C	-4.8947	-1.4001	0.0000
H	-7.0118	-1.2412	0.0000
H	-4.8953	-2.4817	0.0000
H	-7.0118	1.2412	0.0000
H	-4.8953	2.4817	0.0000
H	-2.4465	-2.4849	0.0000
H	-2.4465	2.4849	0.0000
H	0.0000	-2.4880	0.0000
H	-0.0000	2.4880	0.0000
H	2.4465	-2.4849	0.0000
H	2.4465	2.4849	0.0000
H	4.8953	-2.4817	0.0000
H	7.0118	-1.2412	0.0000
H	7.0118	1.2412	0.0000
H	4.8953	2.4817	0.0000

Table S6: Interaction energy and its energy contributions at the cofacial arrangements, the x- and y-stacked minima, as well as the slip-stacked minima of the acene dimers for fixed intermolecular distance of $\Delta z = 3.4$ Å. The values of Δx and Δy are determined with SAPT0/jun-cc-pVDZ (see Tab. 1 in the main text), the energies are recalculated with sSAPT0/jun-cc-pVDZ and are given in kJ mol^{-1}.

System	Δx [Å]	Δy [Å]	E_{int}	E_{el}	E_{ind}	E_{dsp}	E_{exch}	Position on PES
Ben_2	0.00	0.00	3.27	−14.86	−2.10	−41.98	62.21	cofacial
	1.57	0.90	−11.55	−10.37	−3.56	−31.07	33.44	slip-stacked
	1.74	0.00	−11.79	−10.27	−3.46	−31.73	33.67	x-stacked
	0.00	1.81	−11.55	−10.37	−3.56	−31.07	33.44	y-stacked
Nap_2	0.00	0.00	−11.62	−25.07	−2.74	−79.72	95.90	cofacial
	1.35	0.99	−34.49	−19.53	−5.92	−66.67	57.62	slip-stacked
	1.46	0.00	−32.23	−21.53	−6.33	−71.39	67.02	x-stacked
	0.00	1.53	−31.04	−18.56	−4.99	−65.08	57.58	y-stacked
Ant_2	0.00	0.00	−28.09	−35.43	−3.37	−119.71	130.42	cofacial
	1.31	1.00	−59.88	−28.78	−8.64	−104.08	81.62	slip-stacked
	1.37	0.00	−55.55	−32.06	−9.26	−112.49	98.26	x-stacked
	0.00	1.46	−53.14	−27.02	−6.33	−100.50	80.71	y-stacked
Tet_2	0.00	0.00	−45.89	−45.89	−4.00	−161.12	165.11	cofacial
	1.28	1.00	−86.76	−38.07	−11.63	−142.94	105.88	slip-stacked
	1.33	0.00	−80.34	−42.40	−12.34	−154.61	129.00	x-stacked
	0.00	1.42	−76.73	−35.59	−7.65	−137.21	103.73	y-stacked
Pen_2	0.00	0.00	−64.63	−56.44	−4.64	−203.54	199.98	cofacial
	1.27	1.01	−114.63	−47.30	−14.81	−182.23	129.71	slip-stacked
	1.31	0.00	−106.05	−52.71	−15.52	−197.49	159.67	x-stacked
	0.00	1.40	−101.36	−44.21	−9.04	−174.57	126.44	y-stacked

Table S7: Interaction energy and its energy contributions at the cofacial arrangements, the x- and y-stacked minima, as well as the slip-stacked minima of the acene dimers for fixed intermolecular distance of $\Delta z = 3.4$ Å. The values of Δx and Δy are determined with SAPT0/jun-cc-pVDZ (see Tab. 1 in the main text), the energies are recalculated with SAPT2+/aug-cc-pVDZ and are given in kJ mol^{-1}.

System	Δx [Å]	Δy [Å]	E_{int}	E_{el}	E_{ind}	E_{dsp}	E_{exch}	Position on PES
Ben_2	0.00	0.00	0.54	−16.60	−2.54	−42.62	62.30	cofacial
	1.57	0.90	−12.05	−11.20	−3.82	−31.74	34.71	slip-stacked
	1.74	0.00	−12.19	−11.18	−3.71	−32.34	35.04	x-stacked
	0.00	1.81	−12.05	−11.19	−3.82	−31.74	34.71	y-stacked
Nap_2	0.00	0.00	−13.90	−28.12	−3.12	−79.17	96.52	cofacial
	1.35	0.99	−32.73	−21.13	−6.16	−66.08	60.64	slip-stacked
	1.46	0.00	−30.61	−23.35	−6.57	−70.61	69.92	x-stacked
	0.00	1.53	−30.26	−20.45	−5.26	−64.79	60.25	y-stacked
Ant_2	0.00	0.00	−30.65	−39.72	−3.71	−118.50	131.28	cofacial
	1.31	1.00	−56.21	−30.94	−8.88	−102.51	86.13	slip-stacked
	1.37	0.00	−52.23	−34.73	−9.46	−110.54	102.50	x-stacked
	0.00	1.46	−51.21	−29.64	−6.64	−99.37	84.44	y-stacked
Tet_2	0.00	0.00	−48.98	−51.36	−4.29	−159.40	166.06	cofacial
	1.28	1.00	−81.32	−40.76	−11.86	−140.53	111.83	slip-stacked
	1.33	0.00	−75.53	−45.91	−12.46	−151.69	134.54	x-stacked
	0.00	1.42	−73.77	−38.86	−7.98	−135.36	108.43	y-stacked
Pen_2	0.00	0.00	−68.41	−63.02	−4.87	−201.44	200.91	cofacial
	1.27	1.01						slip-stacked
	1.31	0.00						x-stacked
	0.00	1.40						y-stacked

Table S8: Interaction energy and its energy contributions at the cofacial arrangements, the x- and y-stacked minima, as well as the slip-stacked minima of the acene dimers for fixed intermolecular distance of $\Delta z = 3.4\,\text{Å}$. The values of Δx and Δy are determined with SAPT0/jun-cc-pVDZ (see Tab. 1 in the main text), the energies are recalculated with SAPT2+(3)δMP2/aug-cc-pVTZ and are given in kJ mol^{-1}.

System	Δx [Å]	Δy [Å]	E_{int}	E_{el}	E_{ind}	E_{dsp}	E_{exch}	Position on PES
Ben_2	0.00	0.00	2.88	−13.30	−0.41	−42.32	58.91	cofacial
	1.57	0.90	−10.45	−8.92	−2.69	−31.59	32.76	slip-stacked
	1.74	0.00	−10.55	−8.86	−2.53	−32.21	33.05	x-stacked
	0.00	1.81	−10.45	−8.92	−2.69	−31.59	32.76	y-stacked
Nap_2	0.00	0.00	−9.37	−22.45	0.05	−78.18	91.21	cofacial
	1.35	0.99	−29.63	−16.90	−4.56	−65.35	57.18	slip-stacked
	1.46	0.00	−27.33	−18.68	−4.81	−69.81	65.97	x-stacked
	0.00	1.53	−27.02	−16.11	−3.59	−64.11	56.80	y-stacked
Ant_2	0.00	0.00	−23.98	−31.69	0.49	−116.90	124.11	cofacial
	1.31	1.00						slip-stacked
	1.37	0.00						x-stacked
	0.00	1.46						y-stacked
Tet_2	0.00	0.00						cofacial
	1.28	1.00						slip-stacked
	1.33	0.00						x-stacked
	0.00	1.42						y-stacked
Pen_2	0.00	0.00						cofacial
	1.27	1.01						slip-stacked
	1.31	0.00						x-stacked
	0.00	1.40						y-stacked
